# Supplementary material for: Evaluation of the Desire to Avoid Pregnancy Scale in the UK: a psychometric analysis including predictive validity
Source: BMJ Open. 2022 Jul 25;12(7):e060287. doi: 10.1136/bmjopen-2021-060287 (PMC9328097; doi:10.1136/bmjopen-2021-060287)
Supplement: Supplementary data [file bmjopen-2021-060287supp001.pdf]

Desire to Avoid Pregnancy Scale – wording of UK version

We would like to ask you about your current thoughts and feelings about the idea of becoming pregnant in the next three months and having a baby in the next year.

We know that women have very different thoughts and feelings about pregnancy, and having a baby, that can change over time.

Please remember that there are no right or wrong answers. For each question, choose one answer that seems right at this point in your life.

The following questions ask about your thoughts and feelings about the idea of becoming PREGNANT IN THE NEXT THREE MONTHS.

Even if you do not think you can become pregnant for partner or physical reasons, please imagine how you feel about BECOMING PREGNANT in the next 3 months.

(5 point Likert scale from Strongly agree to Strongly disagree)

I wouldn't mind if I became pregnant in the next 3 months. (Item 1)

It would be a good thing for me if I became pregnant in the next 3 months. (Item 2)

Thinking about becoming pregnant in the next 3 months makes me feel unhappy. (Item 3)

Thinking about becoming pregnant in the next 3 months makes me feel excited. (Item 4)

The next questions ask you to think about your main partner. By main partner, we mean the romantic partner that is the most serious to you.

If you don't have a romantic partner, please think about the last person with whom you were physically intimate (anything from kissing and cuddling to sex) or think of a person you know who you would consider being physically intimate with.

Becoming pregnant in the next 3 months would bring me closer to my main partner. (Item 5)

The following questions ask about your thoughts and feelings about the idea of having a BABY IN THE NEXT YEAR.

Even if you do not think you can have a baby for partner or physical reasons, please imagine how you feel about HAVING A BABY in the next year.

(5 point Likert scale from Strongly agree to Strongly disagree)

I want to have a baby within the next year. (Item 6)

If I had a baby in the next year, it would be bad for my life. (Item 7)

It would be a positive addition to my life to have a baby in the next year. (Item 8)

It would be the end of the world for me to have a baby in the next year. (Item 9)

Thinking about having a baby within the next year makes me smile. (Item 10)

Thinking about having a baby within the next year makes me feel stressed out. (Item 11)

I would feel a loss of freedom if I had a baby in the next year. (Item 12)

If I had a baby in the next year, it would be hard for me to manage raising the child. (Item 13)

I would worry that having a baby in the next year would make it harder for me to achieve other things in my life. (Item 14)

Supplementary file 2

Table of responses to DAP questions showing endorsement and missing data

Item	Wording	Strongly agree	Agree	Neither agree nor disagree	Disagree	Strongly disagree	Missing
1	I wouldn't mind if I became pregnant in the next 3 months	11.3	12.5	6	23.4	46.8	0
2	It would be a good thing for me if I became pregnant in the next 3 months	8.6	5.7	9.2	23.8	52.6	0.1
3	Thinking about becoming pregnant in the next 3 months makes me feel unhappy	12.8	17.4	19.3	23.6	26.8	0.1
4	Thinking about becoming pregnant in the next 3 months makes me feel excited	9.8	12.1	13.3	26.5	38.3	0.1
5	Becoming pregnant in the next 3 months would bring me closer to my main partner	7.4	20.2	26.4	26.4	19.4	0.2
6	I want to have a baby within the next year	11.3	8.4	8.7	26.4	45.3	0.1
7	If I had a baby in the next year, it would be bad for my life	11	21.3	17.8	23	26.8	0.1
8	It would be a positive addition to my life to have a baby in the next year	10.2	18.8	20.4	26.1	24.5	0
9	It would be the end of the world for me to have a baby in the next year	31.7	28.8	15.3	11	13.2	0.1
10	Thinking about having a baby within the next year makes me smile	13.3	18.8	13.5	31.7	22.7	0
11	Thinking about having a baby within the next year makes me feel stressed out	5	12.4	12	40.8	29.7	0.1
12	I would feel a loss of freedom if I had a baby in the next year	7.3	18.7	10.1	31.6	32.2	0.1
13	If I had a baby in the next year, it would be hard for me to manage raising the child	6.3	23.2	12.1	30.3	28.1	0
14	I would worry that having a baby in the next year would make it harder for me to achieve other things in my life	3.8	13.2	8.1	32.6	42.2	0.1

```
                                              Generalised-Item Thresholds
--------------------------------------------------------------------------------------
  6                                           |                                      |
                            XXXXXXXXXXXXXXXXXX|                                      |
                                              |                                      |
  5                                           |                                      |
                                   XXXXXXXXXXX|                                      |
                                              |                                      |
  4                                           |                                      |
                            XXXXXXXXXXXXXXXXXX|                                      |
                                              |                                      |
                           XXXXXXXXXXXXXXXXXXX|                                      |
  3                                          X|9.4                                   |
                              XXXXXXXXXXXXXXXX|                                      |
                             XXXXXXXXXXXXXXXXX|5.4                                   |
                              XXXXXXXXXXXXXXXX|10.4                                  |
  2                      XXXXXXXXXXXXXXXXXXXXX|3.4 7.4 8.4 9.3                       |
                              XXXXXXXXXXXXXXXX|11.4 13.4                             |
                  XXXXXXXXXXXXXXXXXXXXXXXXXXXX|12.4                                  |
                  XXXXXXXXXXXXXXXXXXXXXXXXXXXX|4.4 9.2                               |
                   XXXXXXXXXXXXXXXXXXXXXXXXXXX|14.4                                  |
  1              XXXXXXXXXXXXXXXXXXXXXXXXXXXXX|1.4 5.3 6.4                           |
               XXXXXXXXXXXXXXXXXXXXXXXXXXXXXXX|3.3 7.3 8.3                           |
      XXXXXXXXXXXXXXXXXXXXXXXXXXXXXXXXXXXXXXXX|2.4 10.3 13.3                         |
              XXXXXXXXXXXXXXXXXXXXXXXXXXXXXXXX|                                      |
  0           XXXXXXXXXXXXXXXXXXXXXXXXXXXXXXXX|4.3 7.2 10.2 12.3                      |
                    XXXXXXXXXXXXXXXXXXXXXXXXXX|1.3 3.2 6.3 8.2 9.1 11.3 13.2         |
                         XXXXXXXXXXXXXXXXXXXXX|1.2 5.2 12.2 14.3                     |
                              XXXXXXXXXXXXXXXX|2.3 4.2 6.2                           |
 -1                          XXXXXXXXXXXXXXXXX|11.2 14.2                             |
                                 XXXXXXXXXXXXX|2.2                                   |
                                  XXXXXXXXXXXX|1.1 3.1 6.1 10.1                      |
                                   XXXXXXXXXXX|2.1 4.1 7.1                           |
                               XXXXXXXXXXXXXXX|8.1                                   |
 -2                                         XX|                                      |
                                          XXXX|5.1 12.1                              |
                                           XXX|11.1 13.1                             |
                                           XXX|                                      |
 -3                                           |14.1                                  |
                                             X|                                      |
                                              |                                      |
                                            XX|                                      |
                                              |                                      |
 -4                                           |                                      |
                                           XXX|                                      |
                                              |                                      |
 -5                                           |                                      |
                                         XXXXX|                                      |
 -6                                           |                                      |
======================================================================================
Each 'X' represents 1.8 cases
The labels for thresholds show the levels of item, and category, respectively
======================================================================================
```

Supplementary file S4

DAP items by domain with item fit and location estimate

			Item fit	Item location	
**Item**	**Cognitive Desires and Preferences**	**Wording**	**Weighted MNSQ**	**UK Estimate**	**USA Estimate**
1	I wouldn't mind if I became pregnant in the next 3 months	positive	0.63	-0.304	-0.60
2	It would be a good thing for me if I became pregnant in the next 3 months	positive	0.7	-0.679	-0.92
6	I want to have a baby within the next year	positive	0.71	-0.349	-0.61
7	If I had a baby in the next year, it would be bad for my life	negative	0.76	0.269	0.69
8	It would be a positive addition to my life to have a baby in the next year	positive	0.65	0.238	0
9	It would be the end of the world for me to have a baby in the next year	negative	1.07	**1.593**	**0.93**
	**Affective Feelings and Attitudes**				
3	Thinking about becoming pregnant in the next 3 months makes me feel unhappy	negative	0.89	0.303	0.28
4	Thinking about becoming pregnant in the next 3 months makes me feel excited	positive	0.82	-0.219	-0.36
10	Thinking about having a baby within the next year makes me smile	positive	0.68	0.377	0.24
11	Thinking about having a baby within the next year makes me feel stressed out	negative	1.11	-0.453	-0.16
	**Anticipated Practical Consequences**				
5	Becoming pregnant in the next 3 months would bring me closer to my main partner	positive	2.21	0.261	0
12	I would feel a loss of freedom if I had a baby in the next year	negative	1.48	-0.177	0.42
13	If I had a baby in the next year, it would be hard for me to manage raising the child	negative	1.28	-0.071	0.33
14	I would worry that having a baby in the next year would make it harder for me to achieve other things in my life	negative	1.47	-**0.792***	-0.23
