# Supplementary material for: Evaluation of the Desire to Avoid Pregnancy Scale in the UK: a psychometric analysis including predictive validity
Source: BMJ Open. 2022 Jul 25;12(7):e060287. doi: 10.1136/bmjopen-2021-060287 (PMC9328097; doi:10.1136/bmjopen-2021-060287)
Supplement: Author's manuscript [file bmjopen-2021-060287.draft_revisions.pdf]

BMJ Open is committed to open peer review. As part of this commitment we make the peer review history of every article we publish publicly available.

When an article is published we post the peer reviewers' comments and the authors' responses online. We also post the versions of the paper that were used during peer review. These are the versions that the peer review comments apply to.

The versions of the paper that follow are the versions that were submitted during the peer review process. They are not the versions of record or the final published versions. They should not be cited or distributed as the published version of this manuscript.

BMJ Open is an open access journal and the full, final, typeset and author-corrected version of record of the manuscript is available on our site with no access controls, subscription charges or pay-per-view fees (http://bmjopen.bmj.com).

If you have any questions on BMJ Open's open peer review process please email info.bmjopen@bmj.com

BMJ Open

Evaluation of the Desire to Avoid Pregnancy Scale in the United Kingdom: a psychometric analysis including predictive validity

Journal:	*BMJ Open*
Manuscript ID	bmjopen-2021-060287
Article Type:	Original research
Date Submitted by the Author:	16-Dec-2021

Complete List of Authors:	Hall, Jennifer; University College London, Reproductive Health Research<br>Barrett, Geraldine; University College London, Reproductive Health Research<br>Rocca, Corinne; University of California San Francisco, Advancing New Standards in Reproductive Health

Keywords:	EPIDEMIOLOGY, PUBLIC HEALTH, Reproductive medicine < GYNAECOLOGY

SCHOLARONE™
Manuscripts

I, the Submitting Author has the right to grant and does grant on behalf of all authors of the Work (as defined in the below author licence), an exclusive licence and/or a non-exclusive licence for contributions from authors who are: i) UK Crown employees; ii) where BMJ has agreed a CC-BY licence shall apply, and/or iii) in accordance with the terms applicable for US Federal Government officers or employees acting as part of their official duties; on a worldwide, perpetual, irrevocable, royalty-free basis to BMJ Publishing Group Ltd ("BMJ") its licensees and where the relevant Journal is co-owned by BMJ to the co-owners of the Journal, to publish the Work in this journal and any other BMJ products and to exploit all rights, as set out in our licence.

The Submitting Author accepts and understands that any supply made under these terms is made by BMJ to the Submitting Author unless you are acting as an employee on behalf of your employer or a postgraduate student of an affiliated institution which is paying any applicable article publishing charge ("APC") for Open Access articles. Where the Submitting Author wishes to make the Work available on an Open Access basis (and intends to pay the relevant APC), the terms of reuse of such Open Access shall be governed by a Creative Commons licence – details of these licences and which Creative Commons licence will apply to this Work are set out in our licence referred to above.

Other than as permitted in any relevant BMJ Author's Self Archiving Policies, I confirm this Work has not been accepted for publication elsewhere, is not being considered for publication elsewhere and does not duplicate material already published. I confirm all authors consent to publication of this Work and authorise the granting of this licence.

For peer review only

Title: Evaluation of the Desire to Avoid Pregnancy Scale in the United Kingdom: a psychometric analysis including predictive validity

Authors: Jennifer Anne Hall [1], Geraldine Barrett[1], Corinne Rocca[2].

1 – Reproductive Health Research Department, UCL Elizabeth Garrett Anderson Institute for Women's Health, London, UK

2 – Advancing New Standards in Reproductive Health (ANSIRH), Department of Obstetrics, Gynecology and Reproductive Sciences, University of California, San Francisco (UCSF) School of Medicine, Oakland, CA 94612

Corresponding author: Jennifer Anne Hall, UCL Elizabeth Garrett Anderson Institute for Women's Health, Reproductive Health Research Department Room 237B, 74 Huntley Street, London, WC1E 6AU. Jennifer.hall@ucl.ac.uk

Word count: 4516

For peer review only

Abstract:

Objectives

To evaluate the psychometric performance, including predictive validity, of a UK version of the Desire to Avoid Pregnancy (DAP) scale.

Design

Prospective cohort study for psychometric evaluation

Setting

United Kingdom

Participants

Women in the United Kingdom aged 15 to menopause, who were not pregnant at the time of recruitment in October 2018 were eligible. 994 women completed the baseline survey and 90.2% of women eligible for the 12month survey participated.

Primary and Secondary outcome measures

The DAP scale was assessed according to key measurement properties of validity (construct (structural and hypothesis testing) and criterion (predictive)), reliability (internal consistency using Cronbach's alpha and test-retest using intraclass correlation coefficients) and differential item functioning. Item response and classical test theory methods were used.

Results

The full range of the UK DAP was captured and the scale was acceptable, understandable and showed good targeting. Construct validity was demonstrated on hypothesis testing where women with the highest DAP score were three times more likely to be using contraception. Eighty percent of women with the lowest DAP score became pregnant within 12 months, compared to <1% of those with the highest DAP score. Reliability, both in terms of internal consistency (Cronbach's α 0.96) and test-rest (ICC 0.95), was excellent. Some aspects of structural validity, in relation to model fit with the item-response model, were not met and investigations suggest further exploration of the factor structure of the DAP is required in other samples. Item 5, regarding relationship with a partner showed differential item functioning by age, number of children and relationship group.

Conclusions

For peer review only

The UK DAP is a valid and reliable measure of women's desire to avoid pregnancy and is highly predictive of pregnancy within the next 12 months. Further evaluations should continue the assessment of the factor structure and the utility of item 5.

Article summary

Strengths and Limitations of this study

- The study was conducted on a large, community-based sample.
- The analysis used both item-response and classical test theory methods.
- This is the first evaluation of the DAP to look at its predictive validity in terms of pregnancy occurring within 12 months.
- The women in the sample were relatively highly educated, possibly due to recruitment methods using educational establishments and social media.
- Women were self-defined; further work should explore the DAP with people of other genders.

Key words: public health, reproductive medicine, epidemiology

For peer review only

INTRODUCTION

Despite increasing recognition of the complexity of the construct of pregnancy preferences and ongoing calls for improved measurement[1-6] there has not, until recently, been a psychometrically validated measure to prospectively assess people's preferences about a potential future pregnancy.[7] This absence has hampered researchers' ability to investigate the underlying causes of unintended pregnancy, including how pregnancy preferences affect contraceptive use, and the consequences of unintended pregnancy for people's health and lives. Practically, it also means that there has not been a reliable way to identify who would benefit from contraceptive care and/or preconception advice. This has limited our ability to help individuals prevent unintended pregnancies; consequently the proportion of unplanned pregnancies in the UK remains around 45%.[8]

The Desire to Avoid Pregnancy Scale (DAP) was created and evaluated in the USA.[7] Developed using construct modelling and item response theory (IRT) methods, the DAP is a 14-item tool covering three conceptual domains (1: cognitive preferences, 2: affective feelings and 3: practical consequences) of the construct of desire to avoid pregnancy. Each item has a five-point Likert-scale response from strongly agree to strongly disagree, and averaged scores range from zero (no desire to avoid pregnancy) to four (high desire to avoid pregnancy). Benefits of assessing desire to avoid pregnancy using this measure include that the scale does not assume that people want to or do plan pregnancies, nor that people hold fully formed intentions as it allows the expression of ambiguity and uncertainty.

The rigor of the development and evaluation of the DAP, and the fact that the tool is designed for prospective use (i.e. to identify women who do or do not want a pregnancy in the near future) gives hope that the DAP could be a useful tool to support women to avoid the pregnancies that they do not desire and to plan and prepare for those that they do. To use this tool in the UK, it is first necessary to evaluate it in this setting, which is the aim of this study. In addition, we aimed to calculate the predictive validity of the DAP over one year.

METHODS

Study Setting

The Pregnancy Planning Preparation and Prevention (P3 Study) was set up in late-2018 to collect data from non-pregnant women to evaluate the DAP and other questions about pregnancy

For peer review only - http://bmjopen.bmj.com/site/about/guidelines.xhtml

preferences. Self-identifying women aged 15 and over, living in the UK, who had not gone through the menopause and who were willing to complete surveys over a one-year period were eligible to take part. After consenting, women completed a baseline survey online and were invited by email to complete the survey again every three months for one year. Data collection ceased in December 2019.

Survey Development

In addition to the DAP scale, the questionnaire included obstetric history, contraception use, reproductive autonomy[9] and socio-demographics. Wherever possible we used existing measures or questions, such as socio-demographic questions recommended by the Office for National Statistics' Harmonisation Strategy[10] and fertility questions from Natsal.[11] The same questionnaire was repeated every three months, prefaced with questions asking if the woman was currently pregnant or had been pregnant since the last follow up.

We conducted cognitive interviews to check women's understanding of the DAP questions and of the whole survey. For this, women were recruited using posters in one central London university (for staff and students), one north London academy (for students aged 15-18) and through word of mouth. Women provided written informed consent to take part in the interviews, some of which were face to face (in the university, at the participant's house or in a public meeting space) and some via online video platforms. Interviews were conducted by JH or GB, were audio-recorded and these, plus electronic field notes, were stored in UCL's Data Safe Haven (DSH). The UCL DSH meets relevant information security standards for the secure storage of personal data. A log was kept of the changes made to the survey; all changes to the DAP were discussed and agreed with CR. We made changes iteratively and re-tested until we were satisfied that the whole survey was well understood, and no further changes were needed. We conducted 28 cognitive interviews in April-May 2018 with women aged 16-44 from a range of backgrounds and education levels.

The survey was designed and implemented by JH using the RedCAP platform,[12, 13] hosted on the UCL Data Safe Haven.

Sample size

A recent systematic review highlighted the lack of guidance for sample size calculations for psychometric studies.[14] There are two main methods either a minimum number of subjects a subject to item ratio, with recommendations from 2 to 20 subjects per item, but again with a minimum of 100. The sample does not have to be representative for a valid psychometric analysis.[15] We aimed for 1000 participants (considered excellent on either method) as sufficient to

For peer review only - http://bmjopen.bmj.com/site/about/guidelines.xhtml

produce stable and accurate model parameters for polytomous IRT models under most data conditions.[16]

We expected that 70% of the cohort would complete follow-up at 12 months[17] and that 10% of them would have a pregnancy in that time[8] giving us 70 pregnancies for our analysis of the predictive validity of the DAP.

A cohort of this size was felt to be feasible to recruit, would meet either criterion for the sample size calculation and would comfortably allow the various tests of reliability and validity, including predictive validity.

Recruitment

In October 2018 posters and leaflets were distributed in one central London university (for staff and students), one north London academy (for students aged 15-18), one outreach sexual health service in south London and one termination service in London. We began recruitment on social media (Facebook and Instagram) through a mixture of sharing through networks and paid for advertising on 15th October 2018. Recruitment was paused on 18th October 2018 and further adverts aimed at women aged under 20, non-White, non-Asian, without university level education and in non-professional occupations went out on 22nd October. Recruitment closed on 23rd October 2018 when the target of 1000 participants was met. Two weeks later email invites were sent out to a random subset of women asking them to re-complete the DAP; this survey was set to close when 150 responses had been received. Women received a £5 electronic voucher for every completed survey.

Data cleaning and validation

A small number of anomalies in the data, e.g. unfeasible pregnancy histories or impossible changes in sociodemographic variables, suggested some fabricated data in addition to some genuine errors. These were reviewed on a case-by-case basis and a set of cleaning rules were agreed by the project steering committee. Management of records with multiple inconsistencies was agreed by the committee.

Patient and Public Involvement

We had public involvement in the development of an overall programme of research on pregnancy planning, now known as the P3 Study, of which this study is one aspect. A patient and public involvement group has recently been established and will be involved in the discussion of how these results are taken forward.

For peer review only

Analysis

As the DAP was developed using IRT[7] we sought to conduct the validation using the same methodology. In addition, we carried out some analyses using classical test theory and factor analysis, which may allow future validations to use these methods rather than the less common methods of IRT. We conducted these analyses on the raw average score (0-4), rather than the model-calculated score, in recognition of how the DAP is likely to be used by most researchers in the field.

Acceptability, endorsement and targeting

We assessed the acceptability of the DAP through the cognitive interviews and by assessing rates of missing data: >95% completion was considered acceptable. We looked at the overall score distribution characteristics and examined the responses across each item to check none had an endorsement of >80%.

We examined the Wright map[1] of the DAP scale to see how items fell relative to participant pregnancy preferences levels. We reviewed the pattern of responses per item to check the targeting of the scale.

Construct validity (structural validity)

All items were fitted to a partial credit item response model (PCM) and tested for item fit using a weighted mean-squared fit t-statistic of 0.75-1.33 as a guide for good fit.[18] We checked for monotonicity, i.e., that for each item, women endorsing higher response categories also had higher scores, on average, on the scale overall. We plotted item characteristic curves to ensure that the responses to each item's categories were ordered.

We also used factor analysis to assess construct validity. Given our findings on PCM item fit, which exhibited some evidence of multidimensionality, we split the data in half randomly and conducted exploratory factor analysis on half the sample, followed by confirmatory factor analysis on the other half, as it considered best practice.[19] In both samples we checked the data were suitable for factor analysis with a polychoric matrix, Bartlett test of sphericity and Kaiser-Meyer-Olkin Measure of

[1] A Wright map, or item-person map, displays the item difficulties and person abilities on the same logit scale and it thus a way of visualising the targeting of the test to the sample and the targeting of individual items to persons

For peer review only

Sampling Adequacy.[20] Exploratory item factor analysis using principal factor extraction was conducted on the correlation matrix. Unrotated and oblique rotations were performed, looking for factors with an Eigenvalue greater than one and items with loading of >0.2. Confirmatory factor analysis (CFA) with a generalised structural equation model (gSEM) was carried out to compare the one factor model, a two-factor model of positive vs negative items and a three-factor model based on the underlying domains of the DAP scale. As estimates of model fit are not available in STATA for gSEM models, we compared model fit with the AIC and BIC where lower AIC/BIC values indicate better fit.[21]

Construct validity (hypothesis testing)

To test construct validity by hypothesis testing, we hypothesised that women with a higher DAP score would be more likely to use contraception. We created a logistic regression model with self-reported use of any contraception in the last 30 days as a binary outcome variable (among women who reported sex with a male partner in the last 30 days) and DAP score as the exposure variable.

Criterion validity

We assessed the criterion validity of the DAP via its predictive validity with regards to pregnancy occurring within one year of completion of the measure. We generated a binary yes/no variable for whether a woman had had any pregnancies during the 12month follow up period, regardless of pregnancy outcome or timing. This was used as the binary outcome and DAP score as the predictor in a logistic regression model.

Reliability (internal consistency)

Internal consistency reliability was assessed with the separation reliability coefficient,[18] the Cronbach's α (>0.7) and by calculating item-rest correlations (to check they were all positive and >0.2).[22, 23]

Reliability (test-retest)

We assessed test-retest reliability with a two-way mixed effects intraclass correlation coefficient on women who completed a two-week retest looking for an ICC >0.8.[24]

For peer review only

Differential item functioning

We looked for differential item functioning (DIF) by age, ethnicity, relationship group, and number of children by introducing item-by-trait terms into the IRT measurement model and examining the estimates to see how much the location differs for people with the trait. We used a total logit difference of 0.6 logits as a meaningful difference.[25] We hypothesised that there would be DIF on item 5 (Becoming pregnant in the next 3 months would bring me closer to my main partner) by age, and number of children, but not for ethnicity, and for there to be no DIF on any other items.

Exploratory analysis of factors associated with DAP score

We developed explanatory IRT models for ethnicity (white/non-white), relationship status (no relationship/in a relationship not married/married), age (15-24/25-34/35+) and number of children (0/1/2/3+). We expected no difference in DAP scores for ethnicity and for it to be easier to endorse items (i.e. have a higher desire to avoid pregnancy) if women were not in a relationship, were under 24 or had no children. We checked this by fitting bivariable and multivariable linear regression models.

Analyses were conducted in ACER ConQuest version 5.9.0 and Stata version 17.

Ethical approval and funding

We received ethical approval from the UCL Research Ethics Committee for both the development and testing of the survey (ref 3974.002) and for the online survey with 12month follow up (ref 3974.003). The study was funded by an NIHR Advanced Fellowship held by JH (PDF-2017-10-021).

RESULTS

Participant characteristics

Figure 1 shows how the cohort of 994 was reached from a total of 1337 records that were created in the online survey software.

<<<<<<<<<<< Figure 1 around here >>>>>>>>>>>>>>>>>>

Women were aged 15-50 (median 31, IQR 23-36, mean 29.7), 83.9% were white, 81.6% described themselves as heterosexual and most were married (48.2%) or in a relationship (34%) (Table 1). A

For peer review only

quarter of the women in the cohort had completed school education (i.e. to age 18), 39.1% were educated to undergraduate level and 31% had postgraduate or professional qualifications. More than half (56.7%) had at least one child. The success of the social media recruitment is shown in the distribution of women recruited from across the UK.

		Freq.	Percent			Freq.	Percent
Age group	15-19	139	14	UK region	Channel Islands	1	0.1
	20-24	143	14.4		East England	8	0.8
	25-29	139	14		East Midlands	80	8.05
	30-34	224	22.5		East of England	78	7.85
	35-39	209	21		Greater London	254	25.55
	40-44	78	7.9		North East	66	6.64
	45+	23	2.3		North West	81	8.15
	Missing	39	3.9		Northern Ireland	16	1.61
Relationship	Not in a relationship	152	15.3		Scotland	83	8.35
	In a relationship, but not married	338	34.0		South East	123	12.37
	Married	479	48.2		South West	94	9.46
	Other	1	0.1		Wales	35	3.52
	Missing	24	2.4		West Midlands	60	6.04
Race/ Ethnicity	White	834	83.9		Missing	15	1.51
	Mixed	26	2.6	Number of children	0	430	43.3
	Asian	69	6.9		1	208	20.9
	Black	24	2.4		2	233	23.4
	Other	9	0.9		3+	78	7.9
	Prefer not to say/ Missing	32	3.2		Missing	45	4.5
Completed level of education	School	247	24.9	Sexuality	Heterosexual	811	81.6
	Undergraduate	389	39.1		Not heterosexual	146	14.7
	Postgraduate	309	31.1		Prefer not to say/ Missing	37	3.7

For peer review only

	Other	18	1.8
	Missing	31	3.1

Table 1 Sociodemographic details of the sample

The women who completed the re-test (n=152) were not significantly different by age, relationship status, ethnicity or number of children than those who were not randomly selected into the re-test sample (data not shown).

Cognitive Interviews

The cognitive interviews demonstrated that the DAP was easily understood by women in England. We made minor changes to the syntax that made the flow better when read by women in the UK. The main changes we made were to the wording of the definition of a ‘partner’. First, we added a sentence to say ‘The next question asks about your partner’, we tested several iterations of wording for describing what could be considered as a ‘partner’ and added wording to help women without a current partner to decide how to answer this question. We rephrased ‘sexual relations’, which women understood to be penetrative sex only, as ‘physically intimate’. The wording of the UK DAP is in supplementary file S1.

Acceptability, endorsement and targeting

The full range of DAP scores (0-4) was captured; there was a negative skew, as shown in Figure 2, with more women having higher levels of desire to avoid pregnancy. The mean score was 2.53, standard deviation 1.05, median 2.71 and inter-quartile range 1.86-3.36. Although all questions were optional in our online survey, there was over 95% completion for every DAP item and very little missing data. No item category had >80% endorsement (Supplementary file S2).

<<<<<<<<<<< Figure 2 around here >>>>>>>>>>>>>>>>>>

The Wright map (Supplementary File S3) showed the full range of participant locations with item thresholds falling at all levels, demonstrating good targeting and that the items are capturing the full range of the construct.

Construct validity (structural validity)

Item fit to model

For peer review only

Most items fit the partial credit model, based on the weighted mean square statistic being between 0.76-1.33, but five items did not (Supplementary file S4). It was noted that the two items with misfit indicating greater deviance than expected due to higher discrimination than the model predicted (items with a weighted mean fit of <0.75) were both positively worded items in domain one, cognitive preferences. Conversely, the three items with poorer fit due to lower discrimination (items with a weighted mean fit of >1.33) were in domain three; anticipated practical consequences, and two of them were negatively worded. Item 5, about the partner, had the poorest fit to model, consistent with the original development.[7] Repeating the item-fit analysis with either a two factor (positive v negative items) or three factor (domains) model reduced the item misfit. This suggests that the DAP items may be capturing more than one dimension in this population.

Monotonicity was confirmed i.e. that, as you go up the categories on any given item the average score on other items goes up suggesting excellent internal structure. Item characteristic curves confirmed that the responses to each item's categories were ordered.

Factor Analysis

Tests confirmed the split datasets (n=497 in each) were suitable for factor analysis (Bartlett test of sphericity (p<0.001) and Kaiser-Meyer-Olkin Measure of Sampling Adequacy (0.960)). EFA using principal factor extraction with no rotation conducted on the polychoric factor matrix suggested a one factor model with an Eigenvalue of 9.84 (LR test <0.001) and where this factor explained 91% of the variability in responses. All items strongly loaded on to this factor (loadings >0.7 for all except item 5 which was 0.51). Oblique rotation did not improve model fit.

Confirmatory factor analysis was conducted the one factor model proposed by the EFA and the two and three factor models suggested by the IRT analysis. There was very little difference in the AIC or BIC between the models; the two-factor (positive v negative) was the best fit and three-factor model (domains) was almost identical, followed by the one factor model, however, the differences were small.

Construct validity (hypothesis testing)

As per our hypothesis, among women who had had sex with a male partner in the last 30 days, those with higher DAP scores (greater desire to avoid pregnancy) were significantly more likely to report contraception use in the last 30 days (OR 3.20 95%CI 2.58, 3.98). A predicted 29.5% of women with a

For peer review only

DAP score of zero were using contraception (95%CI 20.6%, 38.5%), compared to 98% (95%CI 96.6%, 98.9%) for women with a DAP score of four.

Criterion validity

Over 90% of women eligible for follow up at 12months completed the survey and 139 women experienced at least one pregnancy during the year of the cohort. For every one-point increase in DAP score there were 0.22 the odds of becoming pregnant within 12months (95%CI 0.17, 0.28). As shown in Figure 3, 80% (95%CI 72.1%, 86.7%) of women with a DAP score of zero became pregnant within a year, compared to 0.89% (95%CI 0.36%, 1.44%) with a score of four.

<<<<<<<<<<<< Figure 3 around here >>>>>>>>>>>>>>>>>>

Reliability (Internal consistency)

The Person Separation Reliability coefficient was 0.91 and Cronbach's α was 0.96, both extremely high. All item-totals were positive and all inter-item correlations were positive and greater than 0.2. Item 5, "Becoming pregnant in the next 3 months would bring me closer to my (main) partner" had the lowest item-test and item-rest correlations (0.506 and 0.441 respectively) but the scale's internal consistency was not reduced (i.e., α remained 0.96) when excluding it.

Reliability (test-retest)

The intraclass correlation coefficient was 0.95 (95%CI 0.92, 0.97) indicating excellent stability over the two-week test-retest period.

Differential Item Functioning

As expected, we found evidence of DIF on item 5 (Becoming pregnant in the next 3 months would bring me closer to my main partner) by age, number of children, and relationship group. For example, compared to participants aged 25-34 with otherwise similar DAP scores, those aged 15-24 were more likely to agree to the statement. There was also evidence of DIF on item 13 (If I had a baby in the next year, it would be hard for me to manage raising the child) by age, number of children and relationship group, with younger women more likely to agree to the statement than older women with otherwise similar DAP scores.

Exploratory analysis of factors associated with DAP score

Both the eIRM and linear regressions showed that there was no significant difference in overall DAP score by ethnicity (β 0.18 95%CI -0.016, 0.38) but there were differences by age, relationship status,

For peer review only

number of children and education level. A multivariable linear regression model was created; ethnicity remained insignificant and all other variables remained statistically significant, as shown in Table 2.

Variable		Coefficient	95% CI		p-value
Relationship status	baseline - married				<0.001
	In a relationship, not married	0.48	0.30	0.65	
	Not in a relationship	0.77	0.53	1.00	
Age	Baseline 15-24				<0.001
	25	-0.63	-0.84	-0.42	
	35	-0.50	-0.75	-0.26	
Number of children	Baseline - 0				<0.001
	1	-0.22	-0.42	-0.01	
	2	0.63	0.40	0.85	
	3	0.81	0.53	1.09	
Level of education	Baseline - School				0.043
	Undergraduate	-0.17	-0.35	0.00	
	Postgraduate	-0.04	-0.23	0.16	
	Other	0.32	-0.13	0.77	

Table 2 Multivariable linear regression of characteristics associated with DAP score

DISCUSSION

The DAP scale generally performed well in the UK population. Cognitive interviews and the high completion rates showed good understanding and acceptability to women. The full range of the score was captured and the scale showed good targeting and acceptable levels of endorsement across item response options. In terms of construct (structural) validity, mean scores increased by each item's increasing categories and the item categories' peaks were ordered on the item characteristic curves. Our construct validity hypothesis test confirmed that for every one-point increase in DAP score women were three times as likely to be using contraception. In addition, the DAP score was highly predictive of pregnancy, with 80% of those with a DAP score of zero becoming pregnant within a year, compared to <1% of those with a DAP score of four. Reliability, both in terms of internal consistency and test-rest, was excellent.

However, there were a few areas where our expectations were not met. Based on the item-fit estimates to the PCM, there was some suggestion that the DAP scale may not be unidimensional.

For peer review only

While EFA suggested a single factor model, CFA found that a two-factor model (i.e. treating positively and negatively worded items as separate factors) fitted marginally better than a three-factor model (i.e. treating each of the DAP's conceptual domains as separate factors), which fitted marginally better than one factor. From a psychometric perspective this suggests that there may be different dimensions within the DAP score, rather than it being unidimensional. Nevertheless, the differences in AIC/BIC were very small and we are limited by the lack of other goodness of fit statistics available in STATA, so we cannot be confident as to which is the best structure. Notably, when fitting the DAP items to a multidimensional PCM treating positively and negatively worded items as different dimensions, all items other than item 5 met criteria for item fit. The DAP is a new measure; this is the first evaluation outside the USA to be published though we are aware of other studies underway. We suggest that these studies conduct further analysis of the factor structure and dimensionality of the DAP based on our findings. Regardless the DAP appears to be functioning well as a measure, predicting pregnancy and passing the usual CTT-based tests.

There were similarities between the UK and USA in that the item that was hardest to endorse was the same in the both settings (Item 9: "It would be the end of the world for me to have a baby in the next year")[7]. In the UK sample however, women found it easier to agree with the items in the conceptual domain of 'anticipated practical consequences' (items 5, 12, 13 and 14) meaning they felt that having a baby in the next year would have a greater impact on their lives, in terms of practical consequences, than the women in the US sample. For example, on item 12 (I would feel a loss of freedom if I had a baby in the next year) 35% of women in the US sample agreed or strongly agreed, compared to 64% in the UK sample. Comparing these items between the two samples suggests a real difference in how different groups of women think about the effects that childbearing would have on their lives under their current circumstances. This may be explained in part by important differences between the populations studied in the UK and USA. The US sample was recruited from reproductive and primary health facilities and targeted women 'at risk' of pregnancy. Women in the US study were younger (41% <25 compared to 28%), less likely to be white (16% v 84%), married (26% vs 48%), or to have completed college-level education (10% vs 70%) and were slightly more likely to already have a child (60.8% vs 57.3%).[7] These are all factors which are known to influence pregnancy preferences and translated to a different distribution of the DAP score, with women in the UK sample expressing a higher desire to avoid pregnancy (averaged raw scores: USA mean 2.2, SD 1.1 vs UK mean 2.53, SD 1.05).

Our analyses pointed to relatively poor psychometric performance of item 5 (pregnancy would bring me closer to my main partner) in several ways, consistent with the USA study.[7] In our cognitive interviews, respondents without a partner found the item difficult to respond to; some opted for the

For peer review only

neutral ‘neither agree nor disagree’ response while others selected ‘strongly disagree’ as they had no partner to become closer to. Item 5 was the item we made the most changes to in terms of the wording, much of which was aimed at how women without a partner should think about this question. Psychometrically, the item did not fit the PCM, and illustrated notable DIF by several sociodemographic variables. Although partnership factors are a key component shaping an individual’s pregnancy preferences, it is likely that people’s perceptions of how a pregnancy would affect a relationship could be reflected in their responses to other DAP items. Removal of this item should be considered, and as our analysis showed, would not reduce the high internal consistency of the scale.

Multivariable analysis has shown that women’s desire to avoid pregnancy is associated with her age, relationship status and number of children, even when controlled for each other, which is consistent with the wider literature on the determinants of pregnancy intention[8, 26-31]. Future analyses could consider how DAP changes over time and as external factors, such as relationship status and education level, change.

Strengths and Limitations

This is a large study with data collected from women across the UK in the community, rather than from healthcare settings. We captured the full spectrum of desire to avoid pregnancy and are the first to assess the properties of the DAP scale using IRT and CTT methods outside the USA. We conducted a test-retest analysis and, uniquely, examined the predicative validity of the DAP by looking at pregnancy over the subsequent year.

Our recruitment strategy may have contributed to the sample being more educated than average, as we used a college and university as recruitment sites. Furthermore, social media recruitment can result in an over-representation of young white women and disadvantages those without internet access.[32, 33] While the proportion of women from a black or ethnic minority background was commensurate with the general population, in absolute terms this was only 160 women, just 15 of whom experienced a pregnancy during this study. Further work should be conducted in non-white women. In this study women self-defined as such; as family formation changes it will be important to understand how the DAP works for people of all genders.

CONCLUSION

While not all pre-set criteria were met for the IRT tests, the criteria were met when considering the possibility of multidimensionality. CTT tests showed that the measure was highly valid and reliable

For peer review only

by those standards. Therefore, we conclude that the UK DAP is a valid and reliable measure of desire to avoid pregnancy and a useful tool for research. Future evaluations of the DAP should consider removal of item 5 and examine different factor structures in light of our findings. We are the first to show that the DAP score is highly predictive of pregnancy. This is of particular use to those attempting to recruit a preconception cohort or, conversely, women who are unlikely to become pregnant, for example for a pharmacological study. The DAP may also have a potential clinical use, to guide preconception or contraception care. In order to be practical, consideration should be given to a shortened version to make it more amenable for use in busy, time-constrained clinical settings.

For peer review only

Acknowledgements

We would like to thank Dr. Mark Wilson for his support around item response theory-based analyses and Ms Anasztazia Gubijev who supported the follow up of the cohort. We would also like to thank the project steering committee (Prof Judith Stephenson, Prof Anna David, Dr Sue Mann, Dr Sarah Crozier, Dr Natalie Edelman, Dr Anita Coutinho and Mr James Harris) for their input on the data cleaning decisions and overall guidance.

Funding statement

The study was funded by an NIHR Advanced Fellowship held by JH (PDF-2017-10-021).

Competing interests

The authors declare that they have no conflicts of interest.

Author contributions

Jennifer Hall: Conceptualisation, Funding acquisition, Methodology, Investigation, Data Curation, Formal analysis, Writing – Original Draft, Writing – Review and Editing, Project administration.
Geraldine Barrett: Conceptualisation, Methodology, Investigation, Writing – Review and Editing.
Corinne Rocca: Conceptualisation, Methodology, Formal analysis, Writing – Review and Editing.

Data sharing agreement

The dataset will be made available in the UCL Discovery depository, linked to the publication record.

For peer review only

References

1. Aiken, A.R., et al., *Rethinking the Pregnancy Planning Paradigm: Unintended Conceptions or Unrepresentative Concepts?* Perspect Sex Reprod Health, 2016. **48**(3): p. 147-51.
2. Bachrach CA and Newcomer S, *Intended pregnancies and unintended pregnancies: distinct categories or opposite ends of a continuum?* Fam Plann Perspect, 1999. **31**(5): p. 251-2.
3. Barrett G and Wellings K, *Understanding Pregnancy Intentions: A Problem in Evidence Everywhere.* Family Planning Perspectives, 2000. **32**(4): p. 194.
4. Mumford, S.L., et al., *Pregnancy intentions-a complex construct and call for new measures.* Fertil Steril, 2016. **106**(6): p. 1453-1462.
5. Santelli, J., et al., *The measurement and meaning of unintended pregnancy.* Perspect Sex Reprod Health, 2003. **35**(2): p. 94-101.
6. Santelli, J.S., et al., *Toward a multidimensional measure of pregnancy intentions: evidence from the United States.* Stud Fam Plann, 2009. **40**(2): p. 87-100.
7. Rocca, C.H., et al., *Psychometric Evaluation of an Instrument to Measure Prospective Pregnancy Preferences: The Desire to Avoid Pregnancy Scale.* Med Care, 2019. **57**(2): p. 152-158.
8. Wellings, K., et al., *The prevalence of unplanned pregnancy and associated factors in Britain: findings from the third National Survey of Sexual Attitudes and Lifestyles (Natsal-3).* Lancet, 2013. **382**(9907): p. 1807-16.
9. Upadhyay, U.D., et al., *Development and validation of a reproductive autonomy scale.* Stud Fam Plann, 2014. **45**(1): p. 19-41.
10. Government Statistical Service, *Harmonisation Strategy*, O.f.N. Statistics, Editor.: UK.
11. Natsal, *Natsal 3 Questionnaire*. 2012.
12. Harris, P.A., et al., *The REDCap consortium: Building an international community of software platform partners.* J Biomed Inform, 2019. **95**: p. 103208.
13. Harris, P.A., et al., *Research electronic data capture (REDCap)--a metadata-driven methodology and workflow process for providing translational research informatics support.* J Biomed Inform, 2009. **42**(2): p. 377-81.
14. Anthoine, E., et al., *Sample size used to validate a scale: a review of publications on newly-developed patient reported outcomes measures.* Health Qual Life Outcomes, 2014. **12**: p. 176.
15. Kline PA, *Handbook of Test Construction: Introduction to Psychometric Design*. 1986, London, UK: Methuen.
16. Kutscher, T., M. Eid, and C. Crayen, *Sample Size Requirements for Applying Mixed Polytomous Item Response Models: Results of a Monte Carlo Simulation Study.* Front Psychol, 2019. **10**: p. 2494.
17. Hall, J.A., et al., *Reassessing pregnancy intention and its relation to maternal, perinatal and neonatal outcomes in a low-income setting: A cohort study.* PLoS One, 2018. **13**(10): p. e0205487.
18. Wright BD and Masters GN, *Rating Scale Analysis (Rasch Measurement Series)*. 1981, Chicago, IL: MESA Press.
19. Smith, W., et al., *Exploring the factor structure of the Health of the Nation Outcomes Scale (HoNOS) in a sample of patients with schizophrenia, schizotypal and delusional disorders.* Health Qual Life Outcomes, 2017. **15**(1): p. 141.
20. Dziuban CD, *When is a correlation matrix appropriate for factor analysis? Some decision rules.* Psychological Bulletin, 1974. **81**(6): p. 358-361.
21. Raftery AE, *Bayesian model selection in social research.* Sociological Methodology, 1995. **25**: p. 111-163.
22. Cronbach LJ, *Coefficient alpha and the internal structure of tests.* Psychometrika 1951. **16**: p. 297-334.

For peer review only

23. Scientific Advisory Committee of the Medical Outcomes Trust, *Assessing health status and quality-of-life instruments: attributes and review criteria.* Qual Life Res, 2002(11): p. 193-205.
24. Qin, S., et al., *Assessing test-retest reliability of patient-reported outcome measures using intraclass correlation coefficients: recommendations for selecting and documenting the analytical formula.* Qual Life Res, 2019. **28**(4): p. 1029-1033.
25. Paek I, *Investigations of Differential Item Functioning: Comparisons Among Approaches, and Extension to a Multidimensional Context*. 2002, Berkeley: University of California.
26. Hall, J.A., et al., *Prevalence and Determinants of Unintended Pregnancy in Mchinji District, Malawi; Using a Conceptual Hierarchy to Inform Analysis.* PLoS One, 2016. **11**(10): p. e0165621.
27. Hayford, S.R. and K.B. Guzzo, *Age, relationship status, and the planning status of births.* Demographic Research, 2010. **23**: p. 365-398.
28. Huynh, S.T., et al., *Prevalence of and factors associated with unplanned pregnancy among women in Koshu, Japan: cross-sectional evidence from Project Koshu, 2011-2016.* BMC Pregnancy Childbirth, 2020. **20**(1): p. 397.
29. Kost, K. and L. Lindberg, *Pregnancy Intentions, Maternal Behaviors, and Infant Health: Investigating Relationships With New Measures and Propensity Score Analysis.* Demography, 2015. **52**(1): p. 83-111.
30. Lang, A.Y., et al., *Opportunities for enhancing pregnancy planning and preconception health behaviours of Australian women.* Women Birth, 2021. **34**(2): p. e153-e161.
31. Lindberg, L., et al., *Pregnancy Intentions and Maternal and Child Health: An Analysis of Longitudinal Data in Oklahoma.* Maternal and Child Health Journal, 2015. **19**(5): p. 1087-1096.
32. Benedict, C., et al., *Recruitment via social media: advantages and potential biases.* Digit Health, 2019. **5**: p. 2055207619867223.
33. Whitaker, C., S. Stevelink, and N. Fear, *The Use of Facebook in Recruiting Participants for Health Research Purposes: A Systematic Review.* J Med Internet Res, 2017. **19**(8): p. e290.

For peer review only

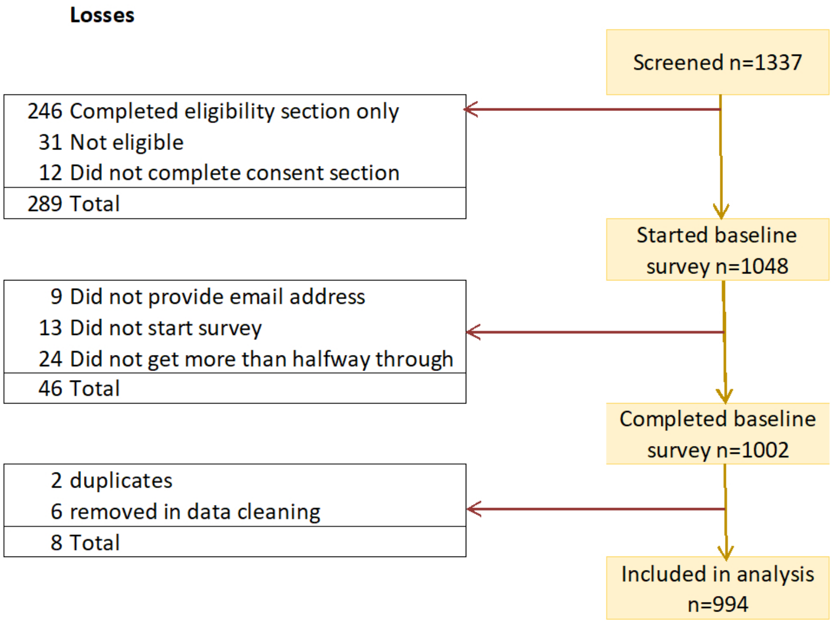

Flow chart of recruitment and inclusion of participants

203x126mm (144 x 144 DPI)

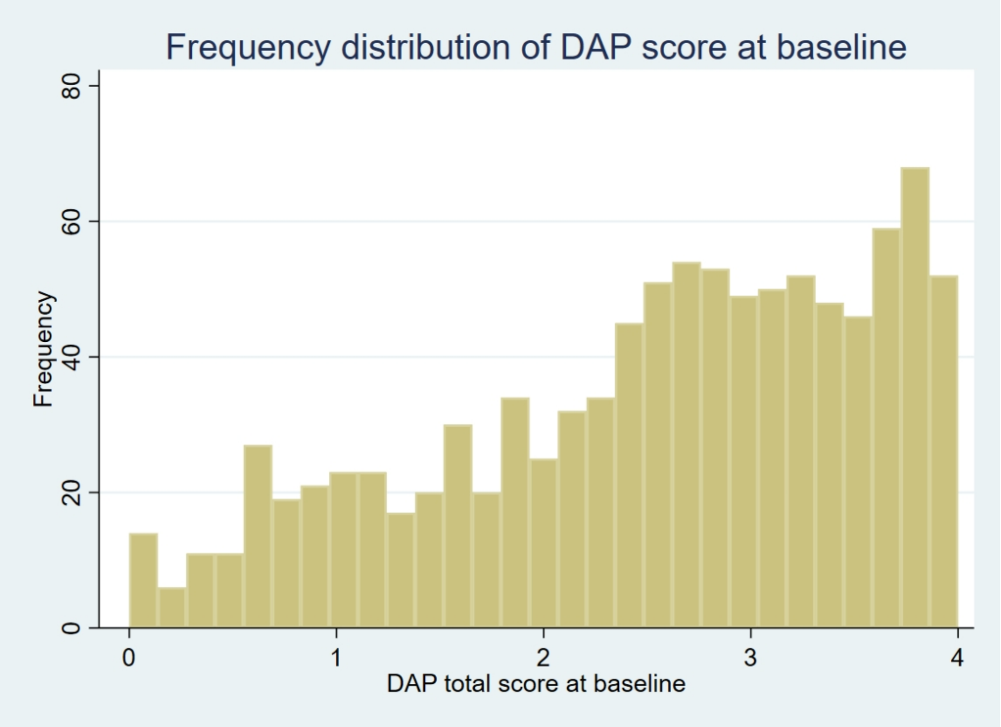

Figure 2 Frequency distribution of DAP score at baseline

411x299mm (72 x 72 DPI)

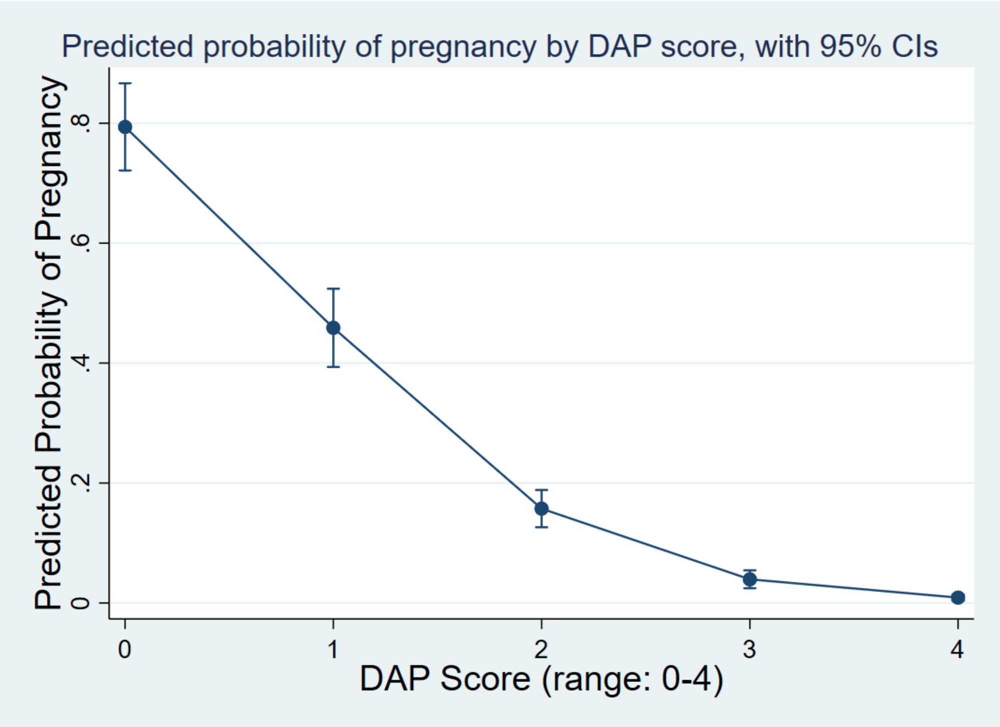

Probability of pregnancy within one year as predicted by DAP score

403x293mm (72 x 72 DPI)

Desire to Avoid Pregnancy Scale – wording of UK version

We would like to ask you about your current thoughts and feelings about the idea of becoming pregnant in the next three months and having a baby in the next year.

We know that women have very different thoughts and feelings about pregnancy, and having a baby, that can change over time.

Please remember that there are no right or wrong answers. For each question, choose one answer that seems right at this point in your life.

The following questions ask about your thoughts and feelings about the idea of becoming PREGNANT IN THE NEXT THREE MONTHS.

Even if you do not think you can become pregnant for partner or physical reasons, please imagine how you feel about BECOMING PREGNANT in the next 3 months.

(5 point Likert scale from Strongly agree to Strongly disagree)

I wouldn't mind if I became pregnant in the next 3 months.

It would be a good thing for me if I became pregnant in the next 3 months.

Thinking about becoming pregnant in the next 3 months makes me feel unhappy.

Thinking about becoming pregnant in the next 3 months makes me feel excited.

The next questions ask you to think about your main partner. By main partner, we mean the romantic partner that is the most serious to you.

If you don't have a romantic partner, please think about the last person with whom you were physically intimate (anything from kissing and cuddling to sex) or think of a person you know who you would consider being physically intimate with.

Becoming pregnant in the next 3 months would bring me closer to my main partner.

The following questions ask about your thoughts and feelings about the idea of having a BABY IN THE NEXT YEAR.

Even if you do not think you can have a baby for partner or physical reasons, please imagine how you feel about HAVING A BABY in the next year.

(5 point Likert scale from Strongly agree to Strongly disagree)

I want to have a baby within the next year.

If I had a baby in the next year, it would be bad for my life.

It would be a positive addition to my life to have a baby in the next year.

It would be the end of the world for me to have a baby in the next year.

Thinking about having a baby within the next year makes me smile.

Thinking about having a baby within the next year makes me feel stressed out.

I would feel a loss of freedom if I had a baby in the next year.

For peer review only

If I had a baby in the next year, it would be hard for me to manage raising the child.

I would worry that having a baby in the next year would make it harder for me to achieve other things in my life.

For peer review only

Supplementary file 2

Table of responses to DAP questions showing endorsement and missing data

Item	Wording	Strongly agree	Agree	Neither agree nor disagree	Disagree	Strongly disagree	Missing
1	I wouldn't mind if I became pregnant in the next 3 months	11.3	12.5	6	23.4	46.8	0
2	It would be a good thing for me if I became pregnant in the next 3 months	8.6	5.7	9.2	23.8	52.6	0.1
3	Thinking about becoming pregnant in the next 3 months makes me feel unhappy	12.8	17.4	19.3	23.6	26.8	0.1
4	Thinking about becoming pregnant in the next 3 months makes me feel excited	9.8	12.1	13.3	26.5	38.3	0.1
5	Becoming pregnant in the next 3 months would bring me closer to my main partner	7.4	20.2	26.4	26.4	19.4	0.2
6	I want to have a baby within the next year	11.3	8.4	8.7	26.4	45.3	0.1
7	If I had a baby in the next year, it would be bad for my life	11	21.3	17.8	23	26.8	0.1
8	It would be a positive addition to my life to have a baby in the next year	10.2	18.8	20.4	26.1	24.5	0
9	It would be the end of the world for me to have a baby in the next year	31.7	28.8	15.3	11	13.2	0.1
10	Thinking about having a baby within the next year makes me smile	13.3	18.8	13.5	31.7	22.7	0
11	Thinking about having a baby within the next year makes me feel stressed out	5	12.4	12	40.8	29.7	0.1
12	I would feel a loss of freedom if I had a baby in the next year	7.3	18.7	10.1	31.6	32.2	0.1
13	If I had a baby in the next year, it would be hard for me to manage raising the child	6.3	23.2	12.1	30.3	28.1	0
14	I would worry that having a baby in the next year would make it harder for me to achieve other things in my life	3.8	13.2	8.1	32.6	42.2	0.1

For peer review only

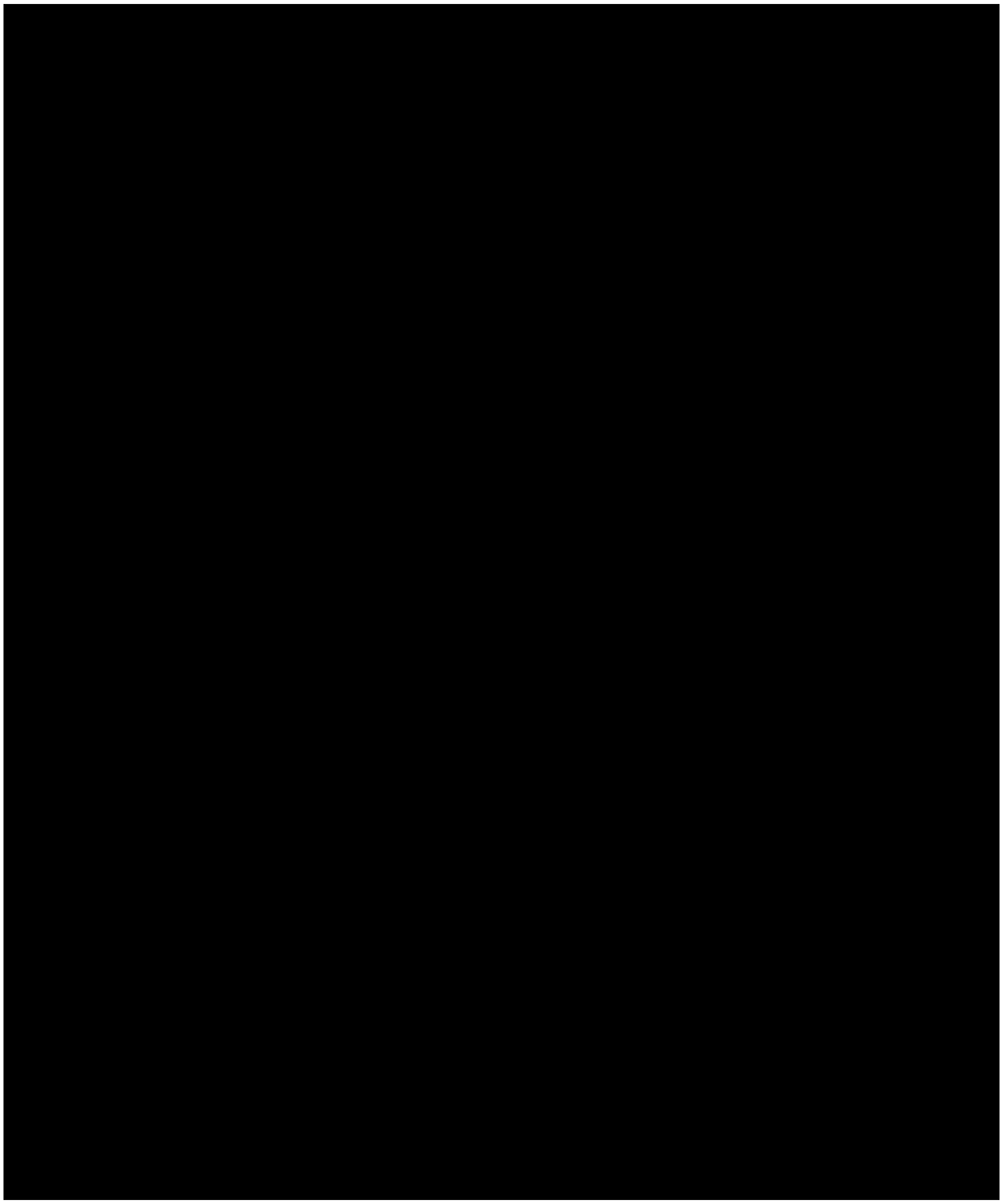

159x191mm (144 x 144 DPI)

Supplementary file S4

DAP items by domain with item fit and location estimate

			Item fit	Item location	
**Item**	**Cognitive Desires and Preferences**	**Wording**	**Weighted MNSQ**	**UK Estimate**	**USA Estimate**
1	I wouldn't mind if I became pregnant in the next 3 months	positive	0.63	-0.304	-0.60
2	It would be a good thing for me if I became pregnant in the next 3 months	positive	0.7	-0.679	-0.92
6	I want to have a baby within the next year	positive	0.71	-0.349	-0.61
7	If I had a baby in the next year, it would be bad for my life	negative	0.76	0.269	0.69
8	It would be a positive addition to my life to have a baby in the next year	positive	0.65	0.238	0
9	It would be the end of the world for me to have a baby in the next year	negative	1.07	**1.593**	**0.93**
	**Affective Feelings and Attitudes**				
3	Thinking about becoming pregnant in the next 3 months makes me feel unhappy	negative	0.89	0.303	0.28
4	Thinking about becoming pregnant in the next 3 months makes me feel excited	positive	0.82	-0.219	-0.36
10	Thinking about having a baby within the next year makes me smile	positive	0.68	0.377	0.24
11	Thinking about having a baby within the next year makes me feel stressed out	negative	1.11	-0.453	-0.16
	**Anticipated Practical Consequences**				
5	Becoming pregnant in the next 3 months would bring me closer to my main partner	positive	2.21	0.261	0
12	I would feel a loss of freedom if I had a baby in the next year	negative	1.48	-0.177	0.42
13	If I had a baby in the next year, it would be hard for me to manage raising the child	negative	1.28	-0.071	0.33
14	I would worry that having a baby in the next year would make it harder for me to achieve other things in my life	negative	1.47	**-0.792***	-0.23

BMJ Open

Evaluation of the Desire to Avoid Pregnancy Scale in the United Kingdom: a psychometric analysis including predictive validity

Journal:	*BMJ Open*
Manuscript ID	bmjopen-2021-060287.R1
Article Type:	Original research
Date Submitted by the Author:	30-Jun-2022
Complete List of Authors:	Hall, Jennifer; University College London, Reproductive Health Research<br>Barrett, Geraldine; University College London, Reproductive Health Research<br>Rocca, Corinne; University of California San Francisco, Advancing New Standards in Reproductive Health
**Primary Subject Heading**:	Reproductive medicine
Secondary Subject Heading:	Public health, Epidemiology, Obstetrics and gynaecology
Keywords:	EPIDEMIOLOGY, PUBLIC HEALTH, Reproductive medicine < GYNAECOLOGY

SCHOLARONE™
Manuscripts

I, the Submitting Author has the right to grant and does grant on behalf of all authors of the Work (as defined in the below author licence), an exclusive licence and/or a non-exclusive licence for contributions from authors who are: i) UK Crown employees; ii) where BMJ has agreed a CC-BY licence shall apply, and/or iii) in accordance with the terms applicable for US Federal Government officers or employees acting as part of their official duties; on a worldwide, perpetual, irrevocable, royalty-free basis to BMJ Publishing Group Ltd ("BMJ") its licensees and where the relevant Journal is co-owned by BMJ to the co-owners of the Journal, to publish the Work in this journal and any other BMJ products and to exploit all rights, as set out in our licence.

The Submitting Author accepts and understands that any supply made under these terms is made by BMJ to the Submitting Author unless you are acting as an employee on behalf of your employer or a postgraduate student of an affiliated institution which is paying any applicable article publishing charge ("APC") for Open Access articles. Where the Submitting Author wishes to make the Work available on an Open Access basis (and intends to pay the relevant APC), the terms of reuse of such Open Access shall be governed by a Creative Commons licence – details of these licences and which Creative Commons licence will apply to this Work are set out in our licence referred to above.

Other than as permitted in any relevant BMJ Author's Self Archiving Policies, I confirm this Work has not been accepted for publication elsewhere, is not being considered for publication elsewhere and does not duplicate material already published. I confirm all authors consent to publication of this Work and authorise the granting of this licence.

For peer review only

Title: Evaluation of the Desire to Avoid Pregnancy Scale in the United Kingdom: a psychometric analysis including predictive validity

Authors: Jennifer Anne Hall [1], Geraldine Barrett[1], Corinne H. Rocca[2].

1 – Reproductive Health Research Department, UCL Elizabeth Garrett Anderson Institute for Women's Health, London, UK

2 – Advancing New Standards in Reproductive Health (ANSIRH), Department of Obstetrics, Gynecology and Reproductive Sciences, University of California, San Francisco (UCSF) School of Medicine, Oakland, CA 94612, USA

Corresponding author: Jennifer Anne Hall, UCL Elizabeth Garrett Anderson Institute for Women's Health, Reproductive Health Research Department Room 237B, 74 Huntley Street, London, WC1E 6AU. Jennifer.hall@ucl.ac.uk

Word count: 4631

For peer review only

For peer review only - http://bmjopen.bmj.com/site/about/guidelines.xhtml

Abstract:

Objectives

To evaluate the psychometric performance, including predictive validity, of a UK version of the Desire to Avoid Pregnancy (DAP) scale.

Design

Prospective cohort study for psychometric evaluation

Setting

United Kingdom

Participants

Women in the United Kingdom aged 15 years to menopause, who were not pregnant at the time of recruitment in October 2018, were eligible. 994 women completed the baseline survey and 90.2% of women eligible for the 12-month survey participated.

Primary and Secondary outcome measures

The DAP scale was assessed according to key measurement properties of validity (construct (structural and hypothesis testing) and criterion (predictive)), reliability (internal consistency using Cronbach's alpha and test-retest using intraclass correlation coefficients) and differential item functioning. Item response and classical test theory methods were used.

Results

The scale was acceptable, understandable and showed good targeting with the full range of scores captured. Construct validity was demonstrated on hypothesis testing, with odds of contraceptive use increasing threefold with each increasing DAP point (range: 0-4). Eighty percent of women with the lowest DAP score became pregnant within 12 months, compared to <1% of those with the highest DAP score. Reliability, both in terms of internal consistency (Cronbach's α 0.96) and test-retest (ICC 0.95), was excellent. Some tests of structural validity, in relation to model fit with the item-response model, were not met, and investigations suggest further exploration of the factor structure of the DAP is required in other samples. Item 5, regarding relationship with a partner, showed differential item functioning by age, number of children and relationship group.

Conclusions

For peer review only

The UK DAP is a valid and reliable measure of women's desire to avoid pregnancy and is highly predictive of pregnancy within the next 12 months. Further evaluations should continue the assessment of the factor structure and the performance of item 5.

Article summary

Strengths and Limitations of this study

- The study was conducted on a large, community-based sample.
- The analysis used both item-response and classical test theory methods.
- This is the first evaluation of the DAP to look at its predictive validity in terms of pregnancy occurring within 12 months.
- The women in the sample were relatively highly educated, possibly due to recruitment methods using educational establishments and social media.
- We did not ask about gender; further work could explore the DAP in relation to gender.

Key words: public health, reproductive medicine, epidemiology

For peer review only - http://bmjopen.bmj.com/site/about/guidelines.xhtml

INTRODUCTION

Despite increasing recognition of the complexity of the construct of pregnancy preferences and ongoing calls for improved measurement[1-6], there has not, until recently, been a psychometrically validated measure to prospectively assess people's preferences about a potential future pregnancy.[7] This absence has hampered researchers' ability to investigate the underlying causes of unintended pregnancy, including how pregnancy preferences affect contraceptive use, and the consequences of unintended pregnancy for people's health and lives. Practically, it has also meant that there has not been a reliable way to identify who would benefit from contraceptive care and/or preconception advice. This gap has limited our ability to help individuals prevent unintended pregnancies; consequently the proportion of unplanned pregnancies in the UK remains around 45%.[8]

The Desire to Avoid Pregnancy Scale (DAP) was created and evaluated in the USA.[7] Developed using construct modelling and item response theory (IRT) methods, the DAP is a 14-item tool covering three conceptual domains (1: cognitive preferences, 2: affective feelings and 3: practical consequences) of the construct of desire to avoid pregnancy. Each item has a five-point Likert-scale response from strongly agree to strongly disagree, and averaged scores range from zero (no desire to avoid pregnancy) to four (high desire to avoid pregnancy). Benefits of assessing desire to avoid pregnancy using this measure include that the scale does not assume that people want to or do plan pregnancies, nor that people hold fully formed intentions as it allows the expression of ambiguity and uncertainty.

The rigor of the development and evaluation of the DAP, and the fact that the tool is designed for prospective use (i.e. to identify women who do or do not want a pregnancy in the near future) gives hope that the DAP could be a useful tool to support women to avoid the pregnancies that they do not desire and to plan and prepare for those that they do. To use this tool in the UK, it is first necessary to evaluate it in this setting, which is the aim of this study. In addition, we aimed to calculate the predictive validity of the DAP in relation to pregnancy over one year.

METHODS

Study Setting

The Pregnancy Planning Preparation and Prevention (P3 Study) was set up in late-2018 to collect data from non-pregnant women to evaluate the DAP and other questions about pregnancy

For peer review only - http://bmjopen.bmj.com/site/about/guidelines.xhtml

preferences. Self-reporting females aged 15 and over, living in the UK, who had not gone through the menopause or been sterilised, and who were willing to complete surveys over a one-year period were eligible to take part. After consenting, women completed a baseline survey online, and they were all invited by email to complete the survey again every three months for one year unless they had an ongoing pregnancy at two consecutive time points. Data collection ceased in December 2019.

Survey Development

In addition to the DAP scale, the questionnaire included obstetric history, contraception use, reproductive autonomy[9] and socio-demographics. Wherever possible we used existing measures or questions, such as socio-demographic questions recommended by the Office for National Statistics' Harmonisation Strategy[10] and fertility questions from Natsal.[11] The same questionnaire was repeated every three months, prefaced with questions asking if the woman was currently pregnant or had been pregnant since the last follow up.

We conducted cognitive interviews to check women's understanding of the DAP questions and of the whole survey. For this, women were recruited using posters in one central London university (for staff and students), one north London academy (for students aged 15-18 years) and through word of mouth. Women provided written informed consent to take part in the interviews, some of which were face to face (in the university, at the participant's house or in a public meeting space) and some via online video platforms. Interviews were conducted by JH or GB, were audio-recorded and these, plus electronic field notes, were stored in UCL's Data Safe Haven (DSH). The UCL DSH meets relevant information security standards for the secure storage of personal data. A log was kept of the changes made to the survey; all changes to the DAP were discussed and agreed with CR. We made changes iteratively and re-tested until we were satisfied that the whole survey was well understood, and no further changes were needed. We conducted 28 cognitive interviews in April-May 2018 with women aged 16-44 from a range of backgrounds and education levels.

The survey was designed and implemented by JH using the RedCAP platform,[12, 13] hosted on the UCL Data Safe Haven.

Sample size

A recent systematic review highlighted the lack of guidance for sample size calculations for psychometric studies.[14] There are two main methods either a minimum number of subjects a subject to item ratio, with recommendations from 2 to 20 subjects per item, but again with a minimum of 100. The sample does not have to be representative for a valid psychometric analysis.[15] We aimed for 1000 participants (considered excellent on either method) as sufficient to

For peer review only

produce stable and accurate model parameters for polytomous IRT models under most data conditions.[16]

We expected that 70% of the cohort would complete follow-up at 12 months[17] and that 10% of them would have a pregnancy in that time[8] giving us 70 pregnancies for our analysis of the predictive validity of the DAP.

A cohort of this size was felt to be feasible to recruit, would meet either criterion for the sample size calculation and would comfortably allow the various tests of reliability and validity, including predictive validity.

Recruitment

In October 2018 posters and leaflets were distributed in one central London university (for staff and students), one north London academy (for students aged 15-18 years), one outreach sexual health service in south London and one termination service in London. We began recruitment on social media (Facebook and Instagram) through a mixture of sharing through networks and paid for advertising on 15th October 2018. Recruitment was paused on 18th October 2018 and further adverts aimed at women aged under 20 years, non-White, non-Asian, without university level education and in non-professional occupations went out on 22nd October. Recruitment closed on 23rd October 2018 when the target of 1000 participants was met. Two weeks later email invites were sent out to a random subset of women asking them to re-complete the DAP; this survey was set to close when 150 responses had been received. Women received a £5 electronic voucher for every completed survey.

Data cleaning and validation

A small number of anomalies in the data, e.g. unfeasible pregnancy histories or impossible changes in sociodemographic variables, suggested some fabricated data in addition to some genuine errors. These were reviewed on a case-by-case basis, and a set of cleaning rules were agreed by the project steering committee. Management of records with multiple inconsistencies was agreed by the committee.

Patient and Public Involvement

We had public involvement in the development of an overall programme of research on pregnancy planning, now known as the P3 Study, of which this study is one aspect. A patient and public

For peer review only

For peer review only - http://bmjopen.bmj.com/site/about/guidelines.xhtml

involvement group has recently been established and will be involved in the discussion of how these results are taken forward.

Analysis

As the DAP was developed using IRT[7] we sought to conduct the validation using the same methodology. In addition, we carried out some analyses using classical test theory and factor analysis, which may be more broadly interpretable than IRT results and may allow future validations to use these methods. We conducted these analyses on the raw average score (range: 0-4) collected at baseline, rather than the model-calculated score, in recognition of how the DAP is likely to be used by most researchers in the field.

Acceptability, endorsement and targeting

We assessed the acceptability of the DAP through the cognitive interviews and by assessing rates of missing data: >95% completion was considered acceptable. We looked at the overall score distribution characteristics and examined the responses across each item to check none had an endorsement of >80%.

We examined the Wright map[1] of the DAP scale to see how item and item threshold locations fell relative to participant pregnancy preferences levels. We reviewed the pattern of responses per item to check the targeting of the scale.

Construct validity (structural validity)

All items were fitted to a partial credit item response model (PCM) and tested for item fit using a weighted mean-squared fit t-statistic of 0.75-1.33 as a guide for good fit.[18] We checked for monotonicity, i.e., that for each item, women endorsing higher response categories also had higher scores, on average, on the scale overall. We plotted item characteristic curves to ensure that the responses to each item's categories were ordered.

We also used factor analysis to assess construct validity. Given our findings on PCM item fit, which exhibited some evidence of multidimensionality, we split the data in half randomly and conducted exploratory factor analysis on half the sample, followed by confirmatory factor analysis on the other

[1] A Wright map, or item-person map, displays the item difficulties and person abilities on the same logit scale and it thus a way of visualising the targeting of the test to the sample and the targeting of individual items to persons

For peer review only - http://bmjopen.bmj.com/site/about/guidelines.xhtml

half, as is considered best practice.[19] In both samples we checked the data were suitable for factor analysis with a polychoric matrix, Bartlett test of sphericity and Kaiser-Meyer-Olkin Measure of Sampling Adequacy.[20] Exploratory item factor analysis using principal factor extraction was conducted on the correlation matrix. Unrotated and oblique rotations were performed, looking for factors with an Eigenvalue greater than one and items with loading of >0.2. Confirmatory factor analysis (CFA) with a generalised structural equation model (gSEM) was carried out to compare the one factor model, a two-factor model of positive vs negative items and a three-factor model based on the underlying domains of the DAP scale. As estimates of model fit are not available in STATA for gSEM models, we compared model fit with the AIC and BIC where lower AIC/BIC values indicate better fit.[21]

Construct validity (hypothesis testing)

To test construct validity by hypothesis testing, we hypothesised that women with a higher DAP score would be more likely to use contraception using baseline data. We created a logistic regression model with self-reported use of any contraception (including all modern contraceptive methods, withdrawal and fertility awareness) in the last 30 days as a binary outcome variable (among women who reported sex with a male partner in the last 30 days) and DAP score as the exposure variable.

Criterion validity

We assessed the criterion validity of the DAP via its predictive validity with regards to pregnancy occurring within one year of completion of the measure at baseline. We generated a binary yes/no variable for whether a woman had had any pregnancies during the 12month follow up period, as reported at each of the quarterly surveys, regardless of pregnancy outcome (eg miscarriage, termination, ectopic, ongoing or other) or timing. This was used as the binary outcome and DAP score as the predictor in a logistic regression model.

Reliability (internal consistency)

Internal consistency reliability was assessed with the separation reliability coefficient (Person Separation Index) calculated from fitting the item responses to the PCM),[18] the Cronbach's α (>0.7) and by calculating item-rest correlations (to check they were all positive and >0.2).[22, 23]

For peer review only - http://bmjopen.bmj.com/site/about/guidelines.xhtml

Reliability (test-retest)

We assessed test-retest reliability with a two-way mixed effects intraclass correlation coefficient (ICC) on women who completed a two-week retest, looking for an ICC >0.8.[24]

Differential item functioning

We evaluated differential item functioning (DIF) by age (15-24/25-34/35+), ethnicity (white/non-white), relationship group (no relationship/in a relationship not married/married), and number of children (0/1/2/3+) by introducing item-by-trait terms into the IRT measurement model and examining the estimates to see how much the location differed by trait. We used a total logit difference between groups of 0.6 logits as a meaningful difference.[25] Based on findings in the US we hypothesised that there would be DIF on item 5 (Becoming pregnant in the next 3 months would bring me closer to my main partner) by age, and number of children, but not for ethnicity, and for there to be no DIF on any other items.

Exploratory analysis of factors associated with DAP score

We developed explanatory IRT models for ethnicity, relationship status, age and number of children. We expected no difference in DAP scores for ethnicity and for it to be easier to endorse items (i.e. have a higher desire to avoid pregnancy) if women were not in a relationship, were under 24 or had no children. We checked this by fitting bivariable and multivariable linear regression models.

Analyses were conducted in ACER ConQuest version 5.9.0 and Stata version 17.

Ethical approval and funding

We received ethical approval from the UCL Research Ethics Committee for both the development and testing of the survey (ref 3974.002) and for the online survey with 12month follow up (ref 3974.003). The study was funded by an NIHR Advanced Fellowship held by JH (PDF-2017-10-021).

For peer review only

For peer review only - http://bmjopen.bmj.com/site/about/guidelines.xhtml

RESULTS

Participant characteristics

Figure 1 shows how the cohort of 994 was reached from a total of 1337 records that were created in the online survey software. The dataset is available in the UCL Research Data Repository [26].

<<<<<<<<<<< Figure 1 around here >>>>>>>>>>>>>>>>>>

Women were aged 15-50 years (median 31, IQR 23-36, mean 29.7), 83.9% were white, 81.6% described themselves as heterosexual and most were married (48.2%) or in a relationship (34.0%) (Table 1). A quarter of the women in the cohort had completed school education (i.e. to age 18), 39.1% were educated to undergraduate level and 31.1% had postgraduate or professional qualifications. More than half (56.7%) had at least one child. The success of the social media recruitment is shown in the distribution of women recruited from across the UK.

		Freq.	Percent			Freq.	Percent
Age group	15-19	139	14	UK region of residence	Channel Islands	1	0.1
	20-24	143	14.4		East England	8	0.8
	25-29	139	14		East Midlands	80	8.05
	30-34	224	22.5		East of England	78	7.85
	35-39	209	21		Greater London	254	25.55
	40-44	78	7.9		North East	66	6.64
	45+	23	2.3		North West	81	8.15
	Missing	39	3.9		Northern Ireland	16	1.61
Relationship	Not in a relationship	152	15.3		Scotland	83	8.35
	In a relationship, but not married	338	34.0		South East	123	12.37
	Married	479	48.2		South West	94	9.46
	Other	1	0.1		Wales	35	3.52
	Missing	24	2.4		West Midlands	60	6.04
Race/ Ethnicity	White	834	83.9		Missing	15	1.51
	Mixed	26	2.6	Number of	0	430	43.3
	Asian	69	6.9		1	208	20.9

For peer review only - http://bmjopen.bmj.com/site/about/guidelines.xhtml

	Black	24	2.4	**children**	2	233	23.4
	Other	9	0.9		3+	78	7.9
	Prefer not to say/ Missing	32	3.2		Missing	45	4.5
**Completed level of education**	School	247	24.9	**Sexuality**	Heterosexual	811	81.6
	Undergraduate	389	39.1		Not heterosexual	146	14.7
	Postgraduate	309	31.1		Prefer not to say/ Missing	37	3.7
	Other	18	1.8				
	Missing	31	3.1				

Table 1 Sociodemographic details of the whole sample at baseline

The women who completed the two-week re-test (n=152) and those who completed follow up at 12 months (over 90% of eligible women) were not significantly different by age, relationship status, ethnicity or number of children than those who were not randomly selected into the re-test sample than the baseline sample (data not shown).

Cognitive Interviews

The cognitive interviews demonstrated that the DAP was easily understood by women in England. We made minor changes to the syntax that made the flow better when read by women in the UK. The main changes we made were to the wording of the definition of a ‘partner’. First, we added a sentence to say ‘The next question asks about your partner’, tested several iterations of wording for describing what could be considered as a ‘partner’ and added wording to help women without a current partner to decide how to answer this question. We rephrased ‘sexual relations’, which women understood to be penetrative sex only, as ‘physically intimate’. The wording of the UK DAP is in supplementary file S1.

Acceptability, endorsement and targeting

The full range of DAP scores (0-4) was captured; there was a negative skew, as shown in Figure 2, with more women having higher levels of desire to avoid pregnancy. The mean score was 2.53, standard deviation 1.05, median 2.71 and inter-quartile range 1.86-3.36. Although all questions were optional in our online survey, there was over 95% completion for every DAP item and very little missing data. No item category had >80% endorsement (Supplementary file S2).

<<<<<<<<<<< Figure 2 around here >>>>>>>>>>>>>>>>>>

For peer review only

The Wright map (Supplementary File S3) showed the full range of participant locations with item thresholds falling at all levels of participants' pregnancy preferences, demonstrating good targeting and that the items captured the full range of the construct.

Construct validity (structural validity)

Item fit to model

Most items fit the partial credit model, based on the weighted mean square statistic being between 0.76-1.33, but five items did not (Supplementary file S4). It was noted that the two items with misfit indicating greater deviance than expected due to higher discrimination than the model predicted (items with a weighted mean fit of <0.75) were both positively worded items in domain one, cognitive preferences. Conversely, the three items with poorer fit due to lower discrimination (items with a weighted mean fit of >1.33) were in domain three; anticipated practical consequences, and two of them were negatively worded. Item 5, about the partner, had the poorest fit to model, consistent with the original development.[7] Repeating the item-fit analysis with either a two factor (positive v negative items) or three factor (domains) model reduced the item misfit.

Monotonicity was confirmed i.e. that average DAP scores increased among those endorsing each increasing response category on each individual item, indicating excellent internal structure. Item characteristic curves confirmed that the responses to each item's categories were ordered.

Factor Analysis

Tests confirmed the split datasets (n=497 in each) were suitable for factor analysis (Bartlett test of sphericity (p<0.001) and Kaiser-Meyer-Olkin Measure of Sampling Adequacy (0.960)). EFA using principal factor extraction with no rotation conducted on the polychoric factor matrix suggested a one factor model with an Eigenvalue of 9.84 (LR test <0.001) and where this factor explained 91% of the variability in responses. All items strongly loaded on to this factor (loadings >0.7 for all except item 5 which was 0.51). Oblique rotation did not improve model fit.

Confirmatory factor analysis was conducted the one factor model proposed by the EFA and the two and three factor models suggested by the IRT analysis. There was very little difference in the AIC or BIC between the models; the two-factor (positive v negative) was the best fit and three-factor model

For peer review only

For peer review only - http://bmjopen.bmj.com/site/about/guidelines.xhtml

(domains) was almost identical, followed by the one factor model, however, the differences were small.

Construct validity (hypothesis testing)

As per our hypothesis, among women who had had sex with a male partner in the last 30 days, those with higher DAP scores (greater desire to avoid pregnancy) were significantly more likely to report contraception use in the last 30 days (OR 3.20 95%CI 2.58, 3.98). A predicted 29.5% of women with a DAP score of zero were using contraception (95%CI 20.6%, 38.5%), compared to 98% (95%CI 96.6%, 98.9%) for women with a DAP score of four.

Criterion validity

Over 90% of women eligible for follow up at 12months completed the survey and 139 women experienced at least one pregnancy during the year of the cohort. For every one-point increase in DAP score there were 0.22 the odds of becoming pregnant within 12months (95%CI 0.17, 0.28). As shown in Figure 3, women with a DAP score of zero had an 80% (95%CI 72.1%, 86.7%) probability of becoming pregnant within a year, compared to 0.89% (95%CI 0.36%, 1.44%) with a score of four.

<<<<<<<<<<< Figure 3 around here >>>>>>>>>>>>>>>>>

Reliability (Internal consistency)

The Person Separation Reliability coefficient was 0.91 and Cronbach's α was 0.96, both extremely high. All inter-item correlations were positive and all item-total correlations were positive and greater than 0.2. Item 5, "Becoming pregnant in the next 3 months would bring me closer to my (main) partner" had the lowest item-test and item-rest correlations (0.506 and 0.441 respectively) and the scale's internal consistency was not reduced (i.e., α remained 0.96) when excluding it.

Reliability (test-retest)

The intraclass correlation coefficient was 0.95 (95%CI 0.92, 0.97) indicating excellent stability over the two-week test-retest period.

Differential Item Functioning

As expected, we found evidence of DIF on item 5 (Becoming pregnant in the next 3 months would bring me closer to my main partner) by age, number of children, and relationship group. For example, compared to participants aged 25-34 years with otherwise similar DAP scores, those aged 15-24 were more likely to agree to the statement. There was also evidence of DIF on item 13 (If I had

For peer review only - http://bmjopen.bmj.com/site/about/guidelines.xhtml

a baby in the next year, it would be hard for me to manage raising the child) by age, number of children and relationship group, with younger women more likely to agree to the statement than older women with otherwise similar DAP scores and those with more children or in a relationship less likely to agree with the statement than those with fewer children or not in a relationship.

Exploratory analysis of factors associated with DAP score

Both the eIRM and linear regressions showed that there was no significant difference in overall DAP score by ethnicity (β 0.18 95%CI -0.02, 0.38) but there were differences by age, relationship status, number of children and education level. A multivariable linear regression model was created; ethnicity remained non-significant and was excluded from the model; all other variables remained statistically significant, as shown in Table 2.

For peer review only

Variable		Coefficient	95% CI		p-value
**Relationship status**	**Baseline - married**				<0.001
	In a relationship, not married	0.48	0.30	0.65	
	Not in a relationship	0.77	0.53	1.00	
**Age**	**Baseline 15-24**				<0.001
	25	-0.63	-0.84	-0.42	
	35	-0.50	-0.75	-0.26	
**Number of children**	**Baseline - 0**				<0.001
	1	-0.22	-0.42	-0.01	
	2	0.63	0.40	0.85	
	3	0.81	0.53	1.09	
**Level of education**	**Baseline - School**				0.043
	Undergraduate	-0.17	-0.35	0.00	
	Postgraduate	-0.04	-0.23	0.16	
	Other	0.32	-0.13	0.77	

Table 2 Multivariable linear regression of characteristics associated with DAP score

DISCUSSION

The DAP scale generally performed well in the UK population. Cognitive interviews and the high completion rates showed good understanding and acceptability to women. The DAP items captured the full range of participants' pregnancy preference levels, and the scale showed good targeting and acceptable levels of endorsement across item response options. In terms of construct (structural)

For peer review only - http://bmjopen.bmj.com/site/about/guidelines.xhtml

validity, mean scores increased by each item's increasing categories and the item categories' peaks were ordered on the item characteristic curves. Our construct validity hypothesis test confirmed that for every one-point increase in DAP score, women were three times as likely to be using contraception. In addition, the DAP score was highly predictive of pregnancy, with 80% of those with a DAP score of zero likely to become pregnant within a year, compared to <1% of those with a DAP score of four. Reliability, both in terms of internal consistency and test-retest, was excellent.

However, there were a few areas where our expectations were not met. Based on the item-fit estimates to the PCM, there was some suggestion that the DAP scale may not be unidimensional. While EFA suggested a single factor model, CFA found that a two-factor model (i.e. treating positively and negatively worded items as separate factors) fitted marginally better than a three-factor model (i.e. treating each of the DAP's conceptual domains as separate factors), which fitted marginally better than one factor. From a psychometric perspective this suggests that there may be different dimensions within the DAP scale, rather than it being unidimensional. Nevertheless, the differences in AIC/BIC were very small and we are limited by the lack of other goodness of fit statistics available in STATA, so we cannot confidently recommend an improved structure. Notably, when fitting the DAP items to a multidimensional PCM treating positively and negatively worded items as different dimensions, all items other than item 5 met criteria for item fit. The DAP is a new measure; this is the first evaluation outside the USA to be published though we are aware of other studies underway. We suggest that these studies conduct further analysis of the factor structure and dimensionality of the DAP based on our findings. Regardless the DAP appears to be functioning well as a measure, predicting pregnancy and passing the standard CTT-based tests.

There were similarities between the UK and USA in that the item that was hardest to endorse was the same in both settings (Item 9: "It would be the end of the world for me to have a baby in the next year")[7]. In the UK sample however, women found it easier to agree with the items in the conceptual domain of 'anticipated practical consequences' (items 5, 12, 13 and 14) meaning they felt that having a baby in the next year would have a greater impact on their lives, in terms of practical consequences, than the women in the US sample. For example, on item 12 (I would feel a loss of freedom if I had a baby in the next year) 35% of women in the US sample agreed or strongly agreed, compared to 64% in the UK sample. Comparing these items between the two samples suggests a real difference in how different groups of women think about the effects that childbearing would have on their lives under their current circumstances. This may be explained in part by important differences between the populations studied in the UK and USA. The US sample was recruited from reproductive and primary health facilities and targeted women 'at risk' of pregnancy. Women in the US study were younger (41% <25 compared to 28%), less likely to be white (16% v 84%), married

For peer review only - http://bmjopen.bmj.com/site/about/guidelines.xhtml

(26% vs 48%), or to have completed college-level education (10% vs 70%) and were slightly more likely to already have a child (61% vs 57%).[7] These are all factors which are known to influence pregnancy preferences and translated to a different distribution of the DAP score, with women in the UK sample expressing a higher desire to avoid pregnancy (averaged raw scores: USA mean 2.2, SD 1.1 vs UK mean 2.5, SD 1.1).

Our analyses pointed to relatively poor psychometric performance of item 5 (pregnancy would bring me closer to my main partner) in several ways, consistent with the USA study.[7] In our cognitive interviews, respondents without a partner found the item difficult to respond to; some opted for the neutral 'neither agree nor disagree' response while others selected 'strongly disagree' as they had no partner to become closer to. Item 5 was the item we made the most changes to in terms of the wording, much of which was aimed at how women without a partner should think about this question. Psychometrically, the item did not fit the PCM, and illustrated notable DIF by several sociodemographic variables. Although partnership factors are a key component shaping an individual's pregnancy preferences, it is likely that people's perceptions of how a pregnancy would affect a relationship could be reflected in their responses to other DAP items. Removal of this item should be considered, and as our analysis showed, would not reduce the high internal consistency of the scale.

Multivariable analysis has shown that women's desire to avoid pregnancy is associated with age, relationship status and number of children, which is consistent with the wider literature on the determinants of pregnancy intention[8, 27-32]. Future analyses could consider how DAP changes over time and as external factors, such as relationship status and education level, change.

Strengths and Limitations

This is a large study with data collected from women across the UK in the community, rather than from healthcare settings. We captured the full spectrum of desire to avoid pregnancy and are the first to assess the properties of the DAP scale using IRT and CTT methods outside the USA. We conducted a test-retest analysis and, uniquely, examined the predictive validity of the DAP by looking at pregnancy over the subsequent year.

Our recruitment strategy may have contributed to the sample being more educated than average, as we used a college and university as recruitment sites. Furthermore, social media recruitment can result in an over-representation of young white women and disadvantages those without internet access.[33, 34] While the proportion of women from a black or ethnic minority background was commensurate with the general population, in absolute terms this was only 160 women, just 15 of

For peer review only - http://bmjopen.bmj.com/site/about/guidelines.xhtml

whom experienced a pregnancy during this study. Further work should be conducted in non-white women. In this study participants self-reported that they were female, and we did not ask about gender; it will be important to understand how the DAP works for people of all genders.

CONCLUSION

The UK DAP is a valid and reliable measure of desire to avoid pregnancy and a useful tool for research. CTT tests showed that the measure was highly valid and reliable based on established standards. While not all pre-set criteria were met for the IRT tests, the criteria were met when considering the possibility of multidimensionality. Future evaluations of the DAP should consider removal of item 5 and examine different factor structures in light of our findings. We are the first to show that the DAP score is highly predictive of pregnancy. This is of particular use to those attempting to recruit a preconception cohort or, conversely, women who are unlikely to become pregnant, for example for a pharmacological study. The DAP may also have a potential clinical use, to guide preconception or contraception care. In order to be practical, consideration should be given to a shortened version to make it more amenable for use in busy, time-constrained clinical settings.

For peer review only

For peer review only - http://bmjopen.bmj.com/site/about/guidelines.xhtml

Acknowledgements

We would like to thank Dr. Mark Wilson for his support around item response theory-based analyses and Ms Anasztazia Gubijev who supported the follow up of the cohort. We would also like to thank the project steering committee (Prof Judith Stephenson, Prof Anna David, Dr Sue Mann, Dr Sarah Crozier, Dr Natalie Edelman, Dr Anita Coutinho and Mr James Harris) for their input on the data cleaning decisions and overall guidance.

Funding statement

The study was funded by an NIHR Advanced Fellowship held by JH (PDF-2017-10-021).

Competing interests

The authors declare that they have no conflicts of interest.

Author contributions

Jennifer Hall: Conceptualisation, Funding acquisition, Methodology, Investigation, Data Curation, Formal analysis, Writing – Original Draft, Writing – Review and Editing, Project administration.
Geraldine Barrett: Conceptualisation, Methodology, Investigation, Writing – Review and Editing.
Corinne Rocca: Conceptualisation, Methodology, Formal analysis, Writing – Review and Editing.

For peer review only

For peer review only - http://bmjopen.bmj.com/site/about/guidelines.xhtml

References

1. Aiken, A.R., et al., *Rethinking the Pregnancy Planning Paradigm: Unintended Conceptions or Unrepresentative Concepts?* Perspect Sex Reprod Health, 2016. **48**(3): p. 147-51.
2. Bachrach CA and Newcomer S, *Intended pregnancies and unintended pregnancies: distinct categories or opposite ends of a continuum?* Fam Plann Perspect, 1999. **31**(5): p. 251-2.
3. Barrett G and Wellings K, *Understanding Pregnancy Intentions: A Problem in Evidence Everywhere.* Family Planning Perspectives, 2000. **32**(4): p. 194.
4. Mumford, S.L., et al., *Pregnancy intentions-a complex construct and call for new measures.* Fertil Steril, 2016. **106**(6): p. 1453-1462.
5. Santelli, J., et al., *The measurement and meaning of unintended pregnancy.* Perspect Sex Reprod Health, 2003. **35**(2): p. 94-101.
6. Santelli, J.S., et al., *Toward a multidimensional measure of pregnancy intentions: evidence from the United States.* Stud Fam Plann, 2009. **40**(2): p. 87-100.
7. Rocca, C.H., et al., *Psychometric Evaluation of an Instrument to Measure Prospective Pregnancy Preferences: The Desire to Avoid Pregnancy Scale.* Med Care, 2019. **57**(2): p. 152-158.
8. Wellings, K., et al., *The prevalence of unplanned pregnancy and associated factors in Britain: findings from the third National Survey of Sexual Attitudes and Lifestyles (Natsal-3).* Lancet, 2013. **382**(9907): p. 1807-16.
9. Upadhyay, U.D., et al., *Development and validation of a reproductive autonomy scale.* Stud Fam Plann, 2014. **45**(1): p. 19-41.
10. Government Statistical Service, *Harmonisation Strategy*, O.f.N. Statistics, Editor.: UK.
11. Natsal, *Natsal 3 Questionnaire*. 2012.
12. Harris, P.A., et al., *The REDCap consortium: Building an international community of software platform partners.* J Biomed Inform, 2019. **95**: p. 103208.
13. Harris, P.A., et al., *Research electronic data capture (REDCap)--a metadata-driven methodology and workflow process for providing translational research informatics support.* J Biomed Inform, 2009. **42**(2): p. 377-81.
14. Anthoine, E., et al., *Sample size used to validate a scale: a review of publications on newly-developed patient reported outcomes measures.* Health Qual Life Outcomes, 2014. **12**: p. 176.
15. Kline PA, *Handbook of Test Construction: Introduction to Psychometric Design*. 1986, London, UK: Methuen.
16. Kutscher, T., M. Eid, and C. Crayen, *Sample Size Requirements for Applying Mixed Polytomous Item Response Models: Results of a Monte Carlo Simulation Study.* Front Psychol, 2019. **10**: p. 2494.
17. Hall, J.A., et al., *Reassessing pregnancy intention and its relation to maternal, perinatal and neonatal outcomes in a low-income setting: A cohort study.* PLoS One, 2018. **13**(10): p. e0205487.
18. Wright BD and Masters GN, *Rating Scale Analysis (Rasch Measurement Series)*. 1981, Chicago, IL: MESA Press.
19. Smith, W., et al., *Exploring the factor structure of the Health of the Nation Outcomes Scale (HoNOS) in a sample of patients with schizophrenia, schizotypal and delusional disorders.* Health Qual Life Outcomes, 2017. **15**(1): p. 141.
20. Dziuban CD, *When is a correlation matrix appropriate for factor analysis? Some decision rules.* Psychological Bulletin, 1974. **81**(6): p. 358-361.
21. Raftery AE, *Bayesian model selection in social research.* Sociological Methodology, 1995. **25**: p. 111-163.
22. Cronbach LJ, *Coefficient alpha and the internal structure of tests.* Psychometrika 1951. **16**: p. 297-334.

For peer review only - http://bmjopen.bmj.com/site/about/guidelines.xhtml

23. Scientific Advisory Committee of the Medical Outcomes Trust, *Assessing health status and quality-of-life instruments: attributes and review criteria.* Qual Life Res, 2002(11): p. 193-205.

24. Qin, S., et al., *Assessing test-retest reliability of patient-reported outcome measures using intraclass correlation coefficients: recommendations for selecting and documenting the analytical formula.* Qual Life Res, 2019. **28**(4): p. 1029-1033.

25. Paek I, *Investigations of Differential Item Functioning: Comparisons Among Approaches, and Extension to a Multidimensional Context*. 2002, Berkeley: University of California.

[DATASET] 26. Hall, J., *Evaluation of the UK version of the Desire to Avoid Pregnancy scale*. 2022: UCL Research Data Repository. DOI 10.5522/04/20195081

27. Hall, J.A., et al., *Prevalence and Determinants of Unintended Pregnancy in Mchinji District, Malawi; Using a Conceptual Hierarchy to Inform Analysis.* PLoS One, 2016. **11**(10): p. e0165621.

28. Hayford, S.R. and K.B. Guzzo, *Age, relationship status, and the planning status of births.* Demographic Research, 2010. **23**: p. 365-398.

29. Huynh, S.T., et al., *Prevalence of and factors associated with unplanned pregnancy among women in Koshu, Japan: cross-sectional evidence from Project Koshu, 2011-2016.* BMC Pregnancy Childbirth, 2020. **20**(1): p. 397.

30. Kost, K. and L. Lindberg, *Pregnancy Intentions, Maternal Behaviors, and Infant Health: Investigating Relationships With New Measures and Propensity Score Analysis.* Demography, 2015. **52**(1): p. 83-111.

31. Lang, A.Y., et al., *Opportunities for enhancing pregnancy planning and preconception health behaviours of Australian women.* Women Birth, 2021. **34**(2): p. e153-e161.

32. Lindberg, L., et al., *Pregnancy Intentions and Maternal and Child Health: An Analysis of Longitudinal Data in Oklahoma.* Maternal and Child Health Journal, 2015. **19**(5): p. 1087-1096.

33. Benedict, C., et al., *Recruitment via social media: advantages and potential biases.* Digit Health, 2019. **5**: p. 2055207619867223.

34. Whitaker, C., S. Stevelink, and N. Fear, *The Use of Facebook in Recruiting Participants for Health Research Purposes: A Systematic Review.* J Med Internet Res, 2017. **19**(8): p. e290.

For peer review only

For peer review only - http://bmjopen.bmj.com/site/about/guidelines.xhtml

Figure Captions

Figure 1 Flowchart of recruitment and inclusion of participants

Figure 2 Frequency distribution of DAP score at baseline

Figure 3 Probability of pregnancy within one year as predicted by DAP score

For peer review only

For peer review only - http://bmjopen.bmj.com/site/about/guidelines.xhtml

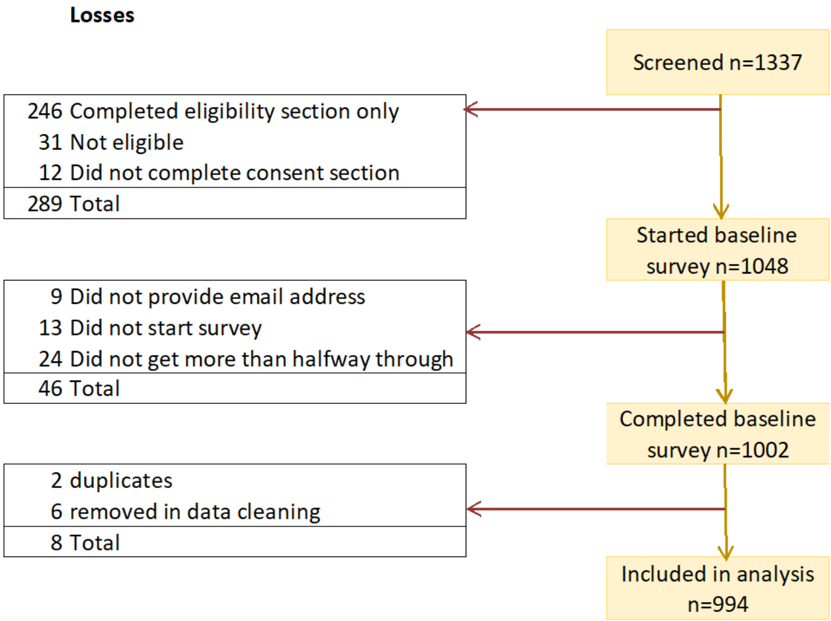

Flow chart of recruitment and inclusion of participants

203x126mm (144 x 144 DPI)

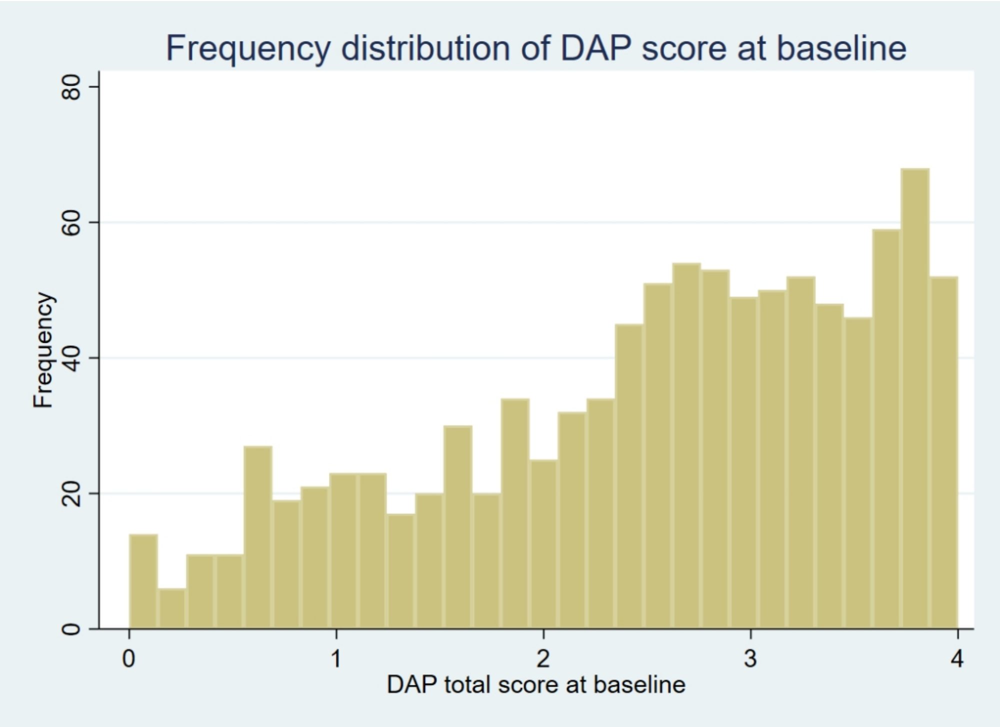

Figure 2 Frequency distribution of DAP score at baseline

411x299mm (72 x 72 DPI)

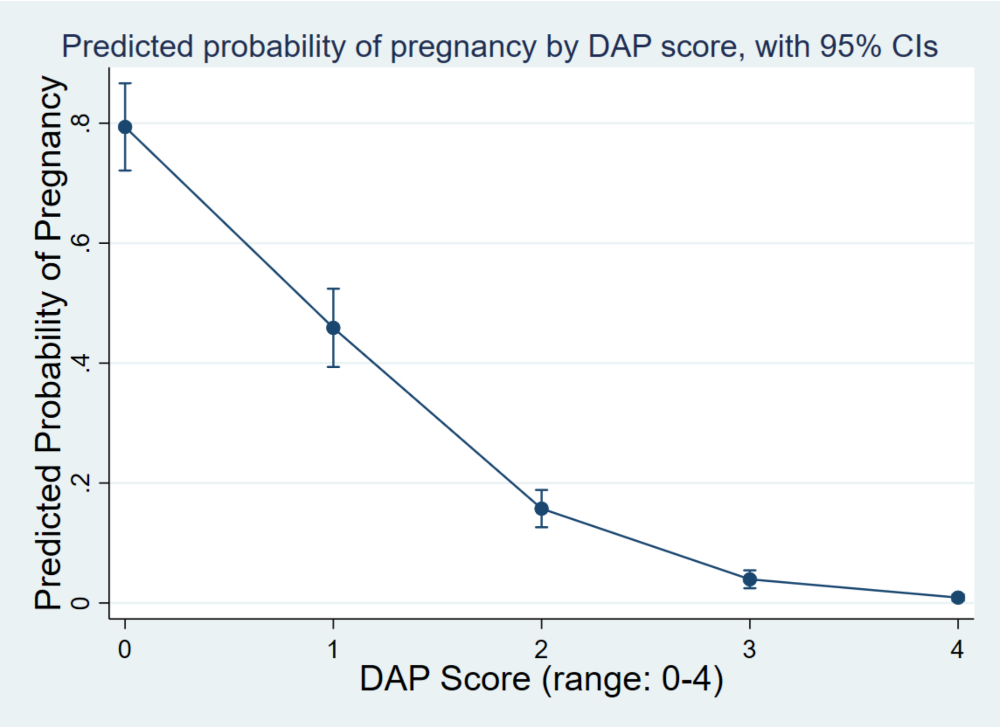

Probability of pregnancy within one year as predicted by DAP score

403x293mm (72 x 72 DPI)

Supplementary file 1

Desire to Avoid Pregnancy Scale – wording of UK version

We would like to ask you about your current thoughts and feelings about the idea of becoming pregnant in the next three months and having a baby in the next year.

We know that women have very different thoughts and feelings about pregnancy, and having a baby, that can change over time.

Please remember that there are no right or wrong answers. For each question, choose one answer that seems right at this point in your life.

The following questions ask about your thoughts and feelings about the idea of becoming PREGNANT IN THE NEXT THREE MONTHS.

Even if you do not think you can become pregnant for partner or physical reasons, please imagine how you feel about BECOMING PREGNANT in the next 3 months.

(5 point Likert scale from Strongly agree to Strongly disagree)

I wouldn't mind if I became pregnant in the next 3 months.

It would be a good thing for me if I became pregnant in the next 3 months.

Thinking about becoming pregnant in the next 3 months makes me feel unhappy.

Thinking about becoming pregnant in the next 3 months makes me feel excited.

The next questions ask you to think about your main partner. By main partner, we mean the romantic partner that is the most serious to you.

If you don't have a romantic partner, please think about the last person with whom you were physically intimate (anything from kissing and cuddling to sex) or think of a person you know who you would consider being physically intimate with.

Becoming pregnant in the next 3 months would bring me closer to my main partner.

The following questions ask about your thoughts and feelings about the idea of having a BABY IN THE NEXT YEAR.

Even if you do not think you can have a baby for partner or physical reasons, please imagine how you feel about HAVING A BABY in the next year.

(5 point Likert scale from Strongly agree to Strongly disagree)

I want to have a baby within the next year.

If I had a baby in the next year, it would be bad for my life.

It would be a positive addition to my life to have a baby in the next year.

It would be the end of the world for me to have a baby in the next year.

Thinking about having a baby within the next year makes me smile.

Thinking about having a baby within the next year makes me feel stressed out.

For peer review only

I would feel a loss of freedom if I had a baby in the next year.

If I had a baby in the next year, it would be hard for me to manage raising the child.

I would worry that having a baby in the next year would make it harder for me to achieve other things in my life.

For peer review only

Supplementary file 2

Table of responses to DAP questions showing endorsement and missing data

Item	Wording	Strongly agree	Agree	Neither agree nor disagree	Disagree	Strongly disagree	Missing
1	I wouldn't mind if I became pregnant in the next 3 months	11.3	12.5	6	23.4	46.8	0
2	It would be a good thing for me if I became pregnant in the next 3 months	8.6	5.7	9.2	23.8	52.6	0.1
3	Thinking about becoming pregnant in the next 3 months makes me feel unhappy	12.8	17.4	19.3	23.6	26.8	0.1
4	Thinking about becoming pregnant in the next 3 months makes me feel excited	9.8	12.1	13.3	26.5	38.3	0.1
5	Becoming pregnant in the next 3 months would bring me closer to my main partner	7.4	20.2	26.4	26.4	19.4	0.2
6	I want to have a baby within the next year	11.3	8.4	8.7	26.4	45.3	0.1
7	If I had a baby in the next year, it would be bad for my life	11	21.3	17.8	23	26.8	0.1
8	It would be a positive addition to my life to have a baby in the next year	10.2	18.8	20.4	26.1	24.5	0
9	It would be the end of the world for me to have a baby in the next year	31.7	28.8	15.3	11	13.2	0.1
10	Thinking about having a baby within the next year makes me smile	13.3	18.8	13.5	31.7	22.7	0
11	Thinking about having a baby within the next year makes me feel stressed out	5	12.4	12	40.8	29.7	0.1
12	I would feel a loss of freedom if I had a baby in the next year	7.3	18.7	10.1	31.6	32.2	0.1
13	If I had a baby in the next year, it would be hard for me to manage raising the child	6.3	23.2	12.1	30.3	28.1	0
14	I would worry that having a baby in the next year would make it harder for me to achieve other things in my life	3.8	13.2	8.1	32.6	42.2	0.1

For peer review only

Supplementary file 3

Wright map

```
                                              Generalised-Item Thresholds
--------------------------------------------------------------------------------------
   6                                          |
                            XXXXXXXXXXXXXXXXXX|
                                              |
   5                                          |
                                   XXXXXXXXXXX|
                                              |
   4                                          |
                            XXXXXXXXXXXXXXXXXX|
                                              |
                           XXXXXXXXXXXXXXXXXXX|
   3                                         X|9.4
                              XXXXXXXXXXXXXXXX|
                             XXXXXXXXXXXXXXXXX|5.4
                              XXXXXXXXXXXXXXXX|10.4
   2                     XXXXXXXXXXXXXXXXXXXXX|3.4 7.4 8.4 9.3
                              XXXXXXXXXXXXXXXX|11.4 13.4
                  XXXXXXXXXXXXXXXXXXXXXXXXXXXX|12.4
                  XXXXXXXXXXXXXXXXXXXXXXXXXXXX|4.4 9.2
                   XXXXXXXXXXXXXXXXXXXXXXXXXXX|14.4
   1             XXXXXXXXXXXXXXXXXXXXXXXXXXXXX|1.4 5.3 6.4
               XXXXXXXXXXXXXXXXXXXXXXXXXXXXXXX|3.3 7.3 8.3
      XXXXXXXXXXXXXXXXXXXXXXXXXXXXXXXXXXXXXXXX|2.4 10.3 13.3
              XXXXXXXXXXXXXXXXXXXXXXXXXXXXXXXX|
   0          XXXXXXXXXXXXXXXXXXXXXXXXXXXXXXXX|4.3 7.2 10.2 12.3
                    XXXXXXXXXXXXXXXXXXXXXXXXXX|1.3 3.2 6.3 8.2 9.1 11.3 13.2
                         XXXXXXXXXXXXXXXXXXXXX|1.2 5.2 12.2 14.3
                              XXXXXXXXXXXXXXXX|2.3 4.2 6.2
  -1                         XXXXXXXXXXXXXXXXX|11.2 14.2
                                 XXXXXXXXXXXXX|2.2
                                  XXXXXXXXXXXX|1.1 3.1 6.1 10.1
                                   XXXXXXXXXXX|2.1 4.1 7.1
                               XXXXXXXXXXXXXXX|8.1
  -2                                        XX|
                                          XXXX|5.1 12.1
                                           XXX|11.1 13.1
                                           XXX|
  -3                                          |14.1
                                             X|
                                              |
                                            XX|
                                              |
  -4                                          |
                                           XXX|
                                              |
  -5                                          |
                                         XXXXX|
  -6                                          |
======================================================================================
Each 'X' represents 1.8 cases
The labels for thresholds show the levels of item, and category, respectively
======================================================================================
```

For peer review only

Supplementary file 4

DAP items by domain with item fit and location estimate

			**Item fit**	**Item location**	
**Item**	**Cognitive Desires and Preferences**	**Wording**	**Weighted MNSQ**	**UK Estimate**	**USA Estimate**
1	I wouldn't mind if I became pregnant in the next 3 months	positive	0.63	-0.304	-0.60
2	It would be a good thing for me if I became pregnant in the next 3 months	positive	0.7	-0.679	-0.92
6	I want to have a baby within the next year	positive	0.71	-0.349	-0.61
7	If I had a baby in the next year, it would be bad for my life	negative	0.76	0.269	0.69
8	It would be a positive addition to my life to have a baby in the next year	positive	0.65	0.238	0
9	It would be the end of the world for me to have a baby in the next year	negative	1.07	**1.593**	**0.93**
	**Affective Feelings and Attitudes**				
3	Thinking about becoming pregnant in the next 3 months makes me feel unhappy	negative	0.89	0.303	0.28
4	Thinking about becoming pregnant in the next 3 months makes me feel excited	positive	0.82	-0.219	-0.36
10	Thinking about having a baby within the next year makes me smile	positive	0.68	0.377	0.24
11	Thinking about having a baby within the next year makes me feel stressed out	negative	1.11	-0.453	-0.16
	**Anticipated Practical Consequences**				
5	Becoming pregnant in the next 3 months would bring me closer to my main partner	positive	2.21	0.261	0
12	I would feel a loss of freedom if I had a baby in the next year	negative	1.48	-0.177	0.42
13	If I had a baby in the next year, it would be hard for me to manage raising the child	negative	1.28	-0.071	0.33
14	I would worry that having a baby in the next year would make it harder for me to achieve other things in my life	negative	1.47	**-0.792***	-0.23

For peer review only

Hall et al. Evaluation of the Desire to Avoid Pregnancy Scale in the United Kingdom: a psychometric analysis including predictive validity. CHECKLIST compatibility

In the absence of the availability of a self-complete checklist document, we list here the how we have met the *COSMIN reporting guidelines for studies on measurement properties of patient reported outcome measures*. Our responses below should be read in conjunction with the pdf of the checklist: https://www.cosmin.nl/wp-content/uploads/COSMIN-reporting-guideline_1.pdf

T1	Yes
T2	Yes
T3	Yes

A1	Yes
A2	Yes
A3	Yes
A4	Yes
A5	Yes
A6	Yes
A7	Yes

I1	Yes
I2	Yes
I3	Yes
I4	Yes
I5	Yes
I6	Yes

GM1	Yes
GM1	Yes
GM3	Yes
GM4	Yes
GM5	Yes
GM6	Yes

For peer review only - http://bmjopen.bmj.com/site/about/guidelines.xhtml

GM7	NA
GM8	NA
GR1	Yes
GR2	Yes
GR3	Yes
D1	Yes
D2	Yes
D3	Yes
D4	Yes
D5	Yes
D6	Yes
C1	Yes
O1	Yes
CV1	NA
CV2	NA
CV3	Yes
CV4	NA
CV5	Yes
CV6	NA
CV7	Yes
SV1	Yes
SV2	Yes
IC1	Yes

For peer review only

For peer review only - http://bmjopen.bmj.com/site/about/guidelines.xhtml

IC2	Yes
IC3	NA
CCV1	NA
CCV2	NA
CCV3	NA
R1	Yes
R2	Yes
R3	NA
ME1	NA
ME2	NA
CriV1	Yes
CriV2	Yes
CriV3	NA
ConV1	NA
ConV2	Yes
ConV3	Yes
ConV4	Yes
ConV5	Yes
Resp1	NA
Resp2	NA
Resp3	NA
Resp4	NA
Resp5	NA
Resp6	NA
Resp7	NA

For peer review only - http://bmjopen.bmj.com/site/about/guidelines.xhtml

Resp8	NA
Resp9	NA

For peer review only

For peer review only - http://bmjopen.bmj.com/site/about/guidelines.xhtml

For peer review only

COSMIN Reporting guideline for studies on measurement properties of patient reported outcome measures

Version August 2021

Joel J Gagnier[1-2]
Jianyu Lai[1]
Lidwine B. Mokkink[3]
Caroline B. Terwee[3]

1. Department of Orthopaedic Surgery, University of Michigan, Ann Arbor, MI, USA
2. Department of Epidemiology, School of Public Health, University of Michigan, Ann Arbor, MI, USA
3. Amsterdam UMC, Vrije Universiteit Amsterdam, Department of Epidemiology and Data Science, Amsterdam Public Health research institute, Amsterdam, Netherlands

Contact
Dr. Joel J. Gagnier, jgagnier@umich.edu
Website: www.cosmin.nl

Table of Content

List of abbreviations	2
Cite & Funding	3
Introduction	4
General reporting recommendations relevant for all studies on measurement properties	5
Specific reporting recommendations for studies on Content Validity	8
Specific reporting recommendations for studies on Structural Validity	8
Specific reporting recommendations for studies on Internal Consistency	8
Specific reporting recommendations for studies on Cross-cultural Validity\Measurement Invariance	9
Specific reporting recommendations for studies on Reliability	10
Specific reporting recommendations for studies on Measurement error	10
Specific reporting recommendations for studies on Criterion Validity	11
Specific reporting recommendations for studies on Hypotheses Testing for Construct Validity	11
Specific reporting recommendations for studies on Responsiveness	11
References	13

List of abbreviations

CTT:	classical test theory
IRT/Rasch:	Item Response Theory and Rasch analyses
NA:	not applicable
Original CC:	original COSMIN checklist [1]
PROM:	patient-reported outcome measure
RoB:	Risk of Bias; it refers to the COSMIN Risk of Bias checklist [2]

For peer review only - http://bmjopen.bmj.com/site/about/guidelines.xhtml

Cite:

Joel J Gagnier, Jianyu Lai, Lidwine B Mokkink, Caroline B Terwee. COSMIN reporting guideline for studies on measurement properties of patient-reported outcome measures. Qual Life Res. 2021 Aug; 30(8):2197-2218. doi: 10.1007/s11136-021-02822-4.

Funding:
This study was funded by a Methods Grant from the Patient-Centered Outcomes Research Institute.

For peer review only

For peer review only - http://bmjopen.bmj.com/site/about/guidelines.xhtml

Introduction

The COSMIN Reporting Guideline is recommended for reporting on studies that evaluate the measurement properties of existing patient-reported outcome measures (PROMs). Adequate reporting of scientific research will increase the applicability of and contributions to scientific knowledge[3]. Studies examining measurement properties are often missing key information that may allow a reader of these studies (e.g., clinician, scientist, funder) to determine what methods were used, what the results are and ultimately what the research means for the evidence of the quality of a particular PROM. This reporting guideline can improve and direct the reporting of studies investigating any measurement properties of PROMs. Improving the reporting of these studies increases their transparency and therefore makes obvious their risk of bias as well as their import to scientific knowledge. This allows accurate methodological assessment of these papers, reliable application of their findings (e.g., to clinical research) and also allows researchers to build on or improve future investigations in the area.

The COSMIN Reporting Guideline was developed as a detailed and specific reporting guideline, for all sections of a manuscript and for all measurement properties that can be investigated within studies exploring the measurement properties of PROMs. More information on this study can be found here[4].

The guideline is a set of 71 items for inclusion in a reporting guideline for studies on measurement properties of PROMs. It contains 35 common recommendations to be used for all studies on any of the measurement properties, and 36 specific recommendations divided into reporting of the specific measurement properties.
The common recommendations are divided into items for reporting the title (n = 3 items), abstract (n = 7), introduction (n = 6), methods (n = 8), results (n = 3), discussion (n = 6), conclusions (n = 1), and other information (n = 1); the specific recommendations are divided into reporting items concerning content validity (n = 7), structural validity (n = 2), internal consistency (n = 3), cross-cultural validity\ measurement invariance (n = 5), reliability (n = 3), measurement error (n = 2), criterion validity (n = 3), hypotheses testing for construct validity (n = 5), responsiveness (n = 6).

We hope all relevant international scientific groups adopt these recommendations and that relevant peer-reviewed journals endorse and enforce them as they have for other reporting recommendations (e.g., CON SORT).

For peer review only - http://bmjopen.bmj.com/site/about/guidelines.xhtml

General Reporting recommendations relevant for all studies on measurement properties		
**Item Number**	**Item Name**	**Item Description**
**Report section: Title**		
T1	Patient Reported Outcome Measure (PROM)	The name of the PROM instrument(s) (and version if relevant) being studied
T2	Measurement Property (MP)	What MPs are being studied or more generally, that MPs are being studied (if there are many properties being investigated, for example)
T3	Study sample	General description of relevant study sample characteristics (e.g., condition of interest, language) and also any intervention or exposure (e.g., treatments) if applicable.
**Report section: Abstract**		
A1	PROM	The name of the PROM instrument(s) (and version if relevant) being studied (i.e. the SF-36 or SF-12; language version) or if it concerns an item bank (e.g., PROMIS instruments). The type of instrument (e.g. a self reported questionnaire or interview).
A2	Measurement Property	What MPs are being studied or more generally, that MPs are being studied (if there are many properties being investigated, for example)
A3	Design	The type of study being used to test the properties (e.g., test-retest design, longitudinal study, cohort, cross sectional, case series, randomized etc.). Other details of the study design if relevant (intervention/exposure, description of comparison instruments, outcomes other than PROMs).
A4	Sample	Inclusion / exclusion criteria. General description of relevant study sample characteristics (e.g., condition of interest, geographic location, language, other relevant demographic and baseline characteristics)
A5	Methods	A brief description of the methods for investigating each MP including statistical analyses
A6	Results	The main results for all MPs investigated reporting statistics for each result with measures of precision where appropriate.
A7	Discussion/Conclusions	A brief description of the results in the context of existing evidence, main strengths and drawbacks and the need for future research on the PROM(s) investigated.
**Report section: Introduction**		
I1	Name and describe the PROM of interest	Specify the name, type, language, and version of the PROM being investigated and how it was developed. Describe the construct the PROM aims to measure and its subscales; describe the structure of the PROM (e.g., the number of factors, the number of items, scoring algorithm); describe relevant instructions (like time period), and number or type of response categories. State whether the PROM is based on a reflective or formative model. Note: This information may also appear in the methods section in greater detail.
I2	Target population	Describe the specific target population that the PROM was designed for. The authors need to provide the appropriate and necessary characteristics of this population.
I3	Citation for the original development of the PROM	The citation for the original development paper(s) should be provided and other highly relevant citations related to the quality of the specific PROM under investigation.
I4	State of Knowledge & Rationale	A description of the current scientific knowledge (what is known) regarding the MPs of? the PROM under investigation. The authors should provide a literature review or refer to a recent review of all existing evidence of the specific version (e.g., language, short

For peer review only - http://bmjopen.bmj.com/site/about/guidelines.xhtml

		form) of the PROM and explain why the new study is necessary and important. The rational for the current proposed study should be given.
I5	Definitions	Specialized terms should be defined or explained.
I6	Objectives and Hypotheses	State the specific objective(s) of the research and hypotheses related to the specific PROM under investigation.
**Report section: General Methods**		
GM1	Study Design	State the key elements of the study design
GM2	Participants	State how the participants were chosen; the inclusion and exclusion criteria. (e.g., if a PROM for a specific condition, then the eligibility and selection criteria should reflect this).
GM3	PROM administration	An explicit description of how and when the PROM(s) were administered (e.g., in what setting) including data collection devices/system used (e.g. paper based, electronic administration / ePRO) should be provided.
GM4	Data collection procedures	Provide information about other data collection, exposure methods (e.g., allocation to interventions) and time points / follow-up points.
GM5	Power/sample size calculation	Provide a power calculation for all MP analyses. Alternatively, if a rule of thumb is used, state it and the source/citation.
GM6	Statistical analyses	Statistical analyses and tests corresponding to all hypotheses or objectives for all MPs should be reported. Where appropriate, a cut-off for statistical significance should be reported (e.g., p-value less than 0.05). A description of all statistics to be used to estimate the magnitude and direction of effect should also be reported, together with measures of variability or precision. Report statistical package used.
GM7	Missing data	State approaches or plan for dealing with missing data.
GM8	Post hoc analysis	The report should specify analyses that used data after the data collection period concluded (i.e., if the analyses were post hoc; secondary data analyses) and describe the rationale for any post hoc analyses.
**Report section: General Results**		
GR1	Missing data	The amount and reasons for missing data should be explained for all analyses for all PROMs (or other outcome measurement instruments) and relevant groups.
GR2	Participant/patient Characteristics	The study patients' characteristics should be described, including baseline PROM scores.
GR3	Sample size	If one study contained analyses using different sample sizes, the authors should report the sample size for each analysis.
**Report section: Discussion**		
D1	MP evidence	Per measurement property the authors should compare the result to the criteria for good measurement properties (e.g., COSMIN criteria)[27], and determine if the specific MP is sufficient or not. Note: This information may also appear in the results section in greater detail in a table for example.
D2	Practical relevance	The authors need to discuss the practical relevance of the findings.
D3	Strengths and limitations	Strengths and limitations of the study should be discussed. For example, discuss if there were any significant potential biases in the study that could have impacted the results.
D4	Generalizability	Generalizability issues related to the PROM results should be discussed. For example, discuss if the results could be generalized to other populations given the sample studied.
D5	Instrument changes	Discuss the need for modifications to the existing PROM or new

For peer review only - http://bmjopen.bmj.com/site/about/guidelines.xhtml

		PROM development. If you conclude that one of the measurement properties is insufficient, you could suggest some modification, or if it is really poor, you could suggest stopping use of the PROM (in the specific population or in general).
D6	Future Research	Report specifically the type of research needed to answer new questions arising out of these findings for the particular MP and PROM investigated.
**Report section: Conclusions**		
C1	Conclusions	State the overall conclusions for each MP and of the use PROM investigated.
**Report section: Other information**		
O1	Conflict of Interest	State any relevant conflict of interest related to the PROM under investigation (e.g., an author being the PROM developer, funding body etc).

For peer review only

For peer review only - http://bmjopen.bmj.com/site/about/guidelines.xhtml

Specific Reporting recommendations for studies on Content Validity		
**Item Number**	**Item Name**	**Item Description**
CV1	Relevance	Report if and how patients and/or professionals were asked whether each item is relevant for their experience with the condition
CV2	Comprehensiveness	Report if and how patients and/or professionals were asked whether all key concepts are included
CV3	Comprehensibility	Report if and how the comprehensibility of the PROM instructions, items, response options, and recall period was assessed
CV4	Relevance results	Report if all items were considered relevant for the construct, population, and context of use of interest by patients and/or professionals
CV5	Response options and recall period	Report whether the response options and recall period were considered appropriate by patients and/or professionals
CV6	Comprehensiveness results	Report whether patients and/or professionals considered all key concepts to be included in the PROM
CV7	Comprehensibility results	Report whether patients understood the PROM instructions, items, and response options as intended

Specific Reporting recommendations for studies on Structural Validity		
**Item Number**	**Item Name**	**Item Description**
SV1	Factor Analyses: Classical Test Theory (CTT) PROMs	Report details of the methods and results for any exploratory or confirmatory factor analyses. State the rational for any explorative factor analyses (e.g., no clear a priori hypotheses). For CFA, describe and justify the factor structure of tested models. Methods and results for checking of the assumptions should be described, the method of estimation, goodness-of-fit statistics and cut-off points for good model fit, including factor loadings of best-fitting model.
SV2	Item Response Theory (IRT) analyses	Type of IRT/Rasch model should be reported. Also report the method of estimation, methods and results for checking of the assumptions (unidimensionality (see factor analysis), local dependency (e.g., residual correlations), monotonicity; (e.g. Mokken scaling), goodness-of-fit statistics, and cut-off points for goodness of item/model fit, and all item parameters.

Specific Reporting recommendations for studies on Internal Consistency		
**Item Number**	**Item Name**	**Item Description**
IC1	Unit of measurement	Report internal consistency methods and results for each unidimensional scale or subscale. Report all evidence or assumptions associated with unidimensionality.
IC2	Continuous scores	Report Cronbach's alpha or omega statistics. Report other statistics calculated for internal consistency of continuous scores.
IC3	Dichotomous scores	Report Cronbach's alpha or Kuder-Richardson coefficient. Report other statistics calculated for internal consistency of dichotomous scores.

For peer review only - http://bmjopen.bmj.com/site/about/guidelines.xhtml

Specific Reporting recommendations for studies on Cross-Cultural Validity\Measurement Invariance		
**Item Number**	**Item Name**	**Item Description**
CCV1	Comparator Group(s)	Report characteristics of (sub)groups being compared. Include sample sizes in each group.
CCV2	Factor Analyses: Classical Test Theory (CTT) PROMs	Report details of the methods and results for multiple-group confirmatory factor analyses, logistic regression analyses, or other analyses performed. Describe and justify the series of tested models, including constraints of factor loadings, intercepts and variances in CFA. Methods and results for checking of the assumptions should be described. criteria to define invariance. Describe the method of estimation, goodness-of-fit statistics and criteria used to flag items for measurement invariance.
CCV3	Item Response Theory (IRT) analyses	Type of IRT/Rasch model should be reported. Also report the methods and results for checking of the assumptions (unidimensionality (see factor analysis), local dependency (e.g., residual correlations), monotonicity; (e.g. Mokken scaling).. Describe statistical packages, method of estimation, criteria used to flag items for DIF, and methods and results of all model comparisons.

For peer review only

Specific Reporting recommendations for studies on Reliability		
**Item Number**	**Item Name**	**Item Description**
R1	PROM Administrations	Report the total number of measurements made and if the measurements were applied to the same samples using the same PROM. The process of administrating the measurements to the patients should be described, including who administered it (i.e., did the patient complete it or was there a proxy), when, how and any time intervals between administrations should be reported. This should include: time interval between repeated measurements (e.g., was the patient stable or not), the test type (e.g. a self-administered questionnaire, an interview-based PROM), the setting in which the instrument was administered (e.g., at the hospital, or at home), and the instructions given for completing it. If relevant, other instruments or measurements accompanying the repeated PROM measurement. Also, if relevant, the independence (whether the PROM was completed without knowledge of the previous scores) of the administrations.
R2	Statistical analyses	All statistical analyses and results specific to the reliability assessment(s) should be described and their use justified (e.g., the intraclass correlation coefficient (ICC) model or type of Kappa coefficient used). Also, describe the variance components, and the weighting scheme used for ordinal scores (e.g., linear or quadratic weights).
R3	Methods to improve reliability	Report any methods used to improve reliability such as restriction of the sample, training of researchers and standardization of methods, and averaging of repeated measurements.

Specific Reporting recommendations for studies on Measurement Error		
**Item Number**	**Item Name**	**Item Description**
ME1	PROM administrations	Report the total number of measurements made and if the measurements were applied to the same samples using the same PROM. The process of administrating the measurements to the patients should be described, including who administered it (i.e., did the patient complete it or was there a proxy), when, how and any time intervals between administrations should be reported. This should include: time interval between repeated measurements (e.g., was the patient stable or not), the test type (e.g. a self-administered questionnaire, an interview-based PROM), the setting in which the instrument was administered (e.g., at the hospital, or at home), and the instructions given for completing it. If relevant, other instruments or measurements accompanying the repeated PROM measurement. Also, if relevant, the independence (whether the PROM was completed without knowledge of the previous completion) of the administrations.
ME2	Statistical analyses	All statistical analyses and results specific to measurement error assessment(s) should be described and their use justified. Specifically, for continuous scores report the Standard Error of Measurement (SEM; Specify the exact model used to calculate the SEM (i.e., SEM consistency or SEM agreement)), Smallest Detectable Change (SDC; specify formula used, included the model of the SEM when based on the SEM) or Limits of Agreement (LoA). For dichotomous/nominal/ordinal scores report marginals (raw data) and the percentage specific (e.g. positive and negative) agreement.

For peer review only - http://bmjopen.bmj.com/site/about/guidelines.xhtml

Specific Reporting recommendations for studies on Criterion Validity		
**Item Number**	**Item Name**	**Item Description**
CriV1	Criterion	Report the details of the criterion used and why it was used. Justification of the gold standard must be reported. Also, describe (if applicable) how and why the criterion was dichotomized or classified. Also, how and when the criterion was administered (e.g., if independent from the PROM).
CriV2	Continuous scores	Report correlations (when criterion has continuous scores) or the area under the receiver operating characteristic (ROC) curve (when criterion is dichotomous).
CriV3	Categorical scores	Described how (and why) the PROM was dichotomized or made into multiple categories. Report sensitivity and specificity statistics.

Specific Reporting recommendations for studies on Hypotheses Testing for Construct Validity		
**Item Number**	**Item Name**	**Item Description**
ConV1	Comparator instrument(s)	The comparator instruments should be appropriately described in terms of the construct(s) they intend to measure. Report the measurement properties of the comparator instruments and related citations or data.
ConV2	Comparator Group(s)	Report characteristics of groups being compared. Include sample sizes in each group.
ConV3	Hypotheses	Report all hypotheses including the direction and magnitude of the expected correlations between the PROM of interest and another measurement instrument, or the direction and magnitude of differences in scores of the PROM between groups.
ConV4	Statistical analyses	Report all statistical methods and results used to test each hypothesis.
ConV5	Results	Report which specific results are in accordance with its hypothesis.

Specific Reporting recommendations for studies on Responsiveness		
**Item Number**	**Item Name**	**Item Description**
Resp1	Comparison Instrument(s)	The comparator instruments should be appropriately described in terms of the construct(s) they intend to measure. Report the measurement properties of the comparator instruments and related citations or data.
Resp2	Comparator Group(s)	Report characteristics of groups being compared. Include sample sizes in each group.
Resp3	Hypotheses	Report all hypotheses including the direction and magnitude of the expected correlations between changes in the PROM of interest and change in another measurement instrument, or the direction and magnitude of differences in change scores of the PROM between groups.
Resp4	Measurement procedures	Report if measurements were applied to the same sample using the same instruments. Describe the measurement procedures, including time intervals between different measurement instruments.
Resp5	Interim period	The interim period between time points should be described.
Resp6	Intervention/Exposure	Describe the intervention given or exposure in the interim period if relevant.
Resp7	Patients changed	Report the proportion of patients that improved or deteriorated (and the details of any anchor used) on the construct measured on all PROMs. Report any changes in

For peer review only

		scores of the PROM in the target population for the research application relative to the predefined hypotheses
Resp8	Statistical analyses	Report all statistical methods and results used to test each hypothesis.
Resp9	Results	Report which specific results are in accordance with its hypothesis.

For peer review only

For peer review only - http://bmjopen.bmj.com/site/about/guidelines.xhtml

References

1. Mokkink LB, Terwee CB, Patrick DL, et al. The COSMIN checklist for assessing the methodological quality of studies on measurement properties of health status measurement instruments: an international Delphi study. *Qual Life Res* 2010;19(4):539-49. doi: 10.1007/s11136-010-9606-8 [doi]
2. Mokkink LB, de Vet HCW, Prinsen CAC, et al. COSMIN Risk of Bias checklist for systematic reviews of Patient-Reported Outcome Measures. *Qual Life Res* 2018;27(5):1171-79. doi: 10.1007/s11136-017-1765-4
3. Ioannidis JP. How to make more published research true. *PLoS Med* 2014;11(10):e1001747. doi: 10.1371/journal.pmed.1001747 [published Online First: 2014/10/22]
4. Gagnier JJ, Lai J, Mokkink LB, et al. COSMIN reporting guideline for studies on measurement properties of patient-reported outcome measures. *Qual Life Res* 2021 doi: 10.1007/s11136-021-02822-4 [published Online First: 2021/04/06]

For peer review only

For peer review only - http://bmjopen.bmj.com/site/about/guidelines.xhtml

Evaluation of the Desire to Avoid Pregnancy Scale in the United Kingdom: a psychometric analysis including predictive validity

Journal:	*BMJ Open*
Manuscript ID	bmjopen-2021-060287.R2
Article Type:	Original research
Date Submitted by the Author:	08-Jul-2022
Complete List of Authors:	Hall, Jennifer; University College London, Reproductive Health Research<br>Barrett, Geraldine; University College London, Reproductive Health Research<br>Rocca, Corinne; University of California San Francisco, Advancing New Standards in Reproductive Health
**Primary Subject Heading**:	Reproductive medicine
Secondary Subject Heading:	Public health, Epidemiology, Obstetrics and gynaecology
Keywords:	EPIDEMIOLOGY, PUBLIC HEALTH, Reproductive medicine < GYNAECOLOGY

I, the Submitting Author has the right to grant and does grant on behalf of all authors of the Work (as defined in the below author licence), an exclusive licence and/or a non-exclusive licence for contributions from authors who are: i) UK Crown employees; ii) where BMJ has agreed a CC-BY licence shall apply, and/or iii) in accordance with the terms applicable for US Federal Government officers or employees acting as part of their official duties; on a worldwide, perpetual, irrevocable, royalty-free basis to BMJ Publishing Group Ltd ("BMJ") its licensees and where the relevant Journal is co-owned by BMJ to the co-owners of the Journal, to publish the Work in this journal and any other BMJ products and to exploit all rights, as set out in our licence.

The Submitting Author accepts and understands that any supply made under these terms is made by BMJ to the Submitting Author unless you are acting as an employee on behalf of your employer or a postgraduate student of an affiliated institution which is paying any applicable article publishing charge ("APC") for Open Access articles. Where the Submitting Author wishes to make the Work available on an Open Access basis (and intends to pay the relevant APC), the terms of reuse of such Open Access shall be governed by a Creative Commons licence – details of these licences and which Creative Commons licence will apply to this Work are set out in our licence referred to above.

Other than as permitted in any relevant BMJ Author's Self Archiving Policies, I confirm this Work has not been accepted for publication elsewhere, is not being considered for publication elsewhere and does not duplicate material already published. I confirm all authors consent to publication of this Work and authorise the granting of this licence.

For peer review only

Title: Evaluation of the Desire to Avoid Pregnancy Scale in the United Kingdom: a psychometric analysis including predictive validity

Authors: Jennifer Anne Hall [1], Geraldine Barrett[1], Corinne H. Rocca[2].

1 – Reproductive Health Research Department, UCL Elizabeth Garrett Anderson Institute for Women's Health, London, UK

2 – Advancing New Standards in Reproductive Health (ANSIRH), Department of Obstetrics, Gynecology and Reproductive Sciences, University of California, San Francisco (UCSF) School of Medicine, Oakland, CA 94612, USA

Corresponding author: Jennifer Anne Hall, UCL Elizabeth Garrett Anderson Institute for Women's Health, Reproductive Health Research Department Room 237B, 74 Huntley Street, London, WC1E 6AU. Jennifer.hall@ucl.ac.uk

Word count: 4646

For peer review only

Abstract:

Objectives

To evaluate the psychometric performance, including predictive validity, of a UK version of the Desire to Avoid Pregnancy (DAP) scale.

Design

Prospective cohort study for psychometric evaluation

Setting

United Kingdom

Participants

Women in the United Kingdom aged 15 years to menopause, who were not pregnant at the time of recruitment in October 2018, were eligible. 994 women completed the baseline survey and 90.2% of women eligible for the 12-month survey participated.

Primary and Secondary outcome measures

The DAP scale was assessed according to key measurement properties of validity (construct (structural and hypothesis testing) and criterion (predictive)), reliability (internal consistency using Cronbach's alpha and test-retest using intraclass correlation coefficients) and differential item functioning. Item response and classical test theory methods were used.

Results

The scale was acceptable, understandable and showed good targeting with the full range of scores captured. Construct validity was demonstrated on hypothesis testing, with odds of contraceptive use increasing threefold with each increasing DAP point (range: 0-4). Eighty percent of women with the lowest DAP score became pregnant within 12 months, compared to <1% of those with the highest DAP score. Reliability, both in terms of internal consistency (Cronbach's α 0.96) and test-retest (ICC 0.95), was excellent. Some tests of structural validity, in relation to model fit with the item-response model, were not met, and investigations suggest further exploration of the factor structure of the DAP is required in other samples. Item 5, regarding relationship with a partner, showed differential item functioning by age, number of children and relationship group.

Conclusions

For peer review only

For peer review only - http://bmjopen.bmj.com/site/about/guidelines.xhtml

The UK DAP is a valid and reliable measure of women's desire to avoid pregnancy and is highly predictive of pregnancy within the next 12 months. Further evaluations should continue the assessment of the factor structure and the performance of the item relating to partner.

Article summary

Strengths and Limitations of this study

- The study was conducted on a large, community-based sample.
- The analysis used both item-response and classical test theory methods.
- This is the first evaluation of the DAP to look at its predictive validity in terms of pregnancy occurring within 12 months.
- The women in the sample were relatively highly educated, possibly due to recruitment methods using educational establishments and social media.
- We did not ask about gender identity; further work could explore the DAP in relation to gender identity.

Key words: public health, reproductive medicine, epidemiology

For peer review only

For peer review only - http://bmjopen.bmj.com/site/about/guidelines.xhtml

INTRODUCTION

Despite increasing recognition of the complexity of the construct of pregnancy preferences and ongoing calls for improved measurement[1-6], there has not, until recently, been a psychometrically validated measure to prospectively assess people's preferences about a potential future pregnancy.[7] This absence has hampered researchers' ability to investigate the underlying causes of unintended pregnancy, including how pregnancy preferences affect contraceptive use, and the consequences of unintended pregnancy for people's health and lives. Practically, it has also meant that there has not been a reliable way to identify who would benefit from contraceptive care and/or preconception advice. This gap has limited our ability to help individuals prevent unintended pregnancies; consequently the proportion of unplanned pregnancies in the UK remains around 45%.[8]

The Desire to Avoid Pregnancy Scale (DAP) was created and evaluated in the USA.[7] Developed using construct modelling and item response theory (IRT) methods, the DAP is a 14-item tool covering three conceptual domains (1: cognitive preferences, 2: affective feelings and 3: practical consequences) of the construct of desire to avoid pregnancy. Each item has a five-point Likert-scale response from strongly agree to strongly disagree, and averaged scores range from zero (no desire to avoid pregnancy) to four (high desire to avoid pregnancy). Benefits of assessing desire to avoid pregnancy using this measure include that the scale does not assume that people want to or do plan pregnancies, nor that people hold fully formed intentions as it allows the expression of ambiguity and uncertainty.

The rigor of the development and evaluation of the DAP, and the fact that the tool is designed for prospective use (i.e. to identify women who do or do not want a pregnancy in the near future) gives hope that the DAP could be a useful tool to support women to avoid the pregnancies that they do not desire and to plan and prepare for those that they do. To use this tool in the UK, it is first necessary to evaluate it in this setting, which is the aim of this study. In addition, we aimed to calculate the predictive validity of the DAP in relation to pregnancy over one year.

METHODS

Study Setting

The Pregnancy Planning Preparation and Prevention (P3 Study) was set up in late-2018 to collect data from non-pregnant women to evaluate the DAP and other questions about pregnancy

For peer review only - http://bmjopen.bmj.com/site/about/guidelines.xhtml

preferences. Self-reporting females aged 15 and over, living in the UK, who had not gone through the menopause or been sterilised, and who were willing to complete surveys over a one-year period were eligible to take part. After consenting, women completed a baseline survey online, and they were all invited by email to complete the survey again every three months for one year unless they had an ongoing pregnancy at two consecutive time points. Data collection ceased in December 2019.

Survey Development

In addition to the DAP scale, the questionnaire included obstetric history, contraception use, reproductive autonomy[9] and socio-demographics. Wherever possible we used existing measures or questions, such as socio-demographic questions recommended by the Office for National Statistics' Harmonisation Strategy[10] and fertility questions from Natsal.[11] The same questionnaire was repeated every three months, prefaced with questions asking if the woman was currently pregnant or had been pregnant since the last follow up.

We conducted cognitive interviews to check women's understanding of the DAP questions and of the whole survey. For this, women were recruited using posters in one central London university (for staff and students), one north London academy (for students aged 15-18 years) and through word of mouth. Women provided written informed consent to take part in the interviews, some of which were face to face (in the university, at the participant's house or in a public meeting space) and some via online video platforms. Interviews were conducted by JH or GB, were audio-recorded and these, plus electronic field notes, were stored in UCL's Data Safe Haven (DSH). The UCL DSH meets relevant information security standards for the secure storage of personal data. A log was kept of the changes made to the survey; all changes to the DAP were discussed and agreed with CR. We made changes iteratively and re-tested until we were satisfied that the whole survey was well understood, and no further changes were needed. We conducted 28 cognitive interviews in April-May 2018 with women aged 16-44 from a range of backgrounds and education levels.

The survey was designed and implemented by JH using the RedCAP platform,[12, 13] hosted on the UCL Data Safe Haven.

Sample size

A recent systematic review highlighted the lack of guidance for sample size calculations for psychometric studies.[14] There are two main methods either a minimum number of subjects a subject to item ratio, with recommendations from 2 to 20 subjects per item, but again with a minimum of 100. The sample does not have to be representative for a valid psychometric analysis.[15] We aimed for 1000 participants (considered excellent on either method) as sufficient to

For peer review only

For peer review only - http://bmjopen.bmj.com/site/about/guidelines.xhtml

produce stable and accurate model parameters for polytomous IRT models under most data conditions.[16]

We expected that 70% of the cohort would complete follow-up at 12 months[17] and that 10% of them would have a pregnancy in that time[8] giving us 70 pregnancies for our analysis of the predictive validity of the DAP.

A cohort of this size was felt to be feasible to recruit, would meet either criterion for the sample size calculation and would comfortably allow the various tests of reliability and validity, including predictive validity.

Recruitment

In October 2018 posters and leaflets were distributed in one central London university (for staff and students), one north London academy (for students aged 15-18 years), one outreach sexual health service in south London and one termination service in London. We began recruitment on social media (Facebook and Instagram) through a mixture of sharing through networks and paid for advertising on 15th October 2018. Recruitment was paused on 18th October 2018 and further adverts aimed at women aged under 20 years, non-White, non-Asian, without university level education and in non-professional occupations went out on 22nd October. Recruitment closed on 23rd October 2018 when the target of 1000 participants was met. Two weeks later email invites were sent out to a random subset of women asking them to re-complete the DAP; this survey was set to close when 150 responses had been received. Women received a £5 electronic voucher for every completed survey.

Data cleaning and validation

A small number of anomalies in the data, e.g. unfeasible pregnancy histories or impossible changes in sociodemographic variables, suggested some fabricated data in addition to some genuine errors. These were reviewed on a case-by-case basis, and a set of cleaning rules were agreed by the project steering committee. Management of records with multiple inconsistencies was agreed by the committee.

Patient and Public Involvement

We had public involvement in the development of an overall programme of research on pregnancy planning, now known as the P3 Study, of which this study is one aspect. A patient and public

For peer review only - http://bmjopen.bmj.com/site/about/guidelines.xhtml

involvement group has recently been established and will be involved in the discussion of how these results are taken forward.

Analysis

As the DAP was developed using IRT[7] we sought to conduct the validation using the same methodology. In addition, we carried out some analyses using classical test theory and factor analysis, which may be more broadly interpretable than IRT results and may allow future validations to use these methods. We conducted these analyses on the raw average score (range: 0-4) collected at baseline, rather than the model-calculated score, in recognition of how the DAP is likely to be used by most researchers in the field.

Acceptability, endorsement and targeting

We assessed the acceptability of the DAP through the cognitive interviews and by assessing rates of missing data: >95% completion was considered acceptable. We looked at the overall score distribution characteristics and examined the responses across each item to check none had an endorsement of >80%.

We examined the Wright map[1] of the DAP scale to see how item and item threshold locations fell relative to participant pregnancy preferences levels. We reviewed the pattern of responses per item to check the targeting of the scale.

Construct validity (structural validity)

All items were fitted to a partial credit item response model (PCM) and tested for item fit using a weighted mean-squared fit t-statistic of 0.75-1.33 as a guide for good fit.[18] We checked for monotonicity, i.e., that for each item, women endorsing higher response categories also had higher scores, on average, on the scale overall. We plotted item characteristic curves to ensure that the responses to each item's categories were ordered.

We also used factor analysis to assess construct validity. Given our findings on PCM item fit, which exhibited some evidence of multidimensionality, we split the data in half randomly and conducted exploratory factor analysis on half the sample, followed by confirmatory factor analysis on the other

[1] A Wright map, or item-person map, displays the item difficulties and person abilities on the same logit scale and it thus a way of visualising the targeting of the test to the sample and the targeting of individual items to persons

For peer review only - http://bmjopen.bmj.com/site/about/guidelines.xhtml

For peer review only

half, as is considered best practice.[19] In both samples we checked the data were suitable for factor analysis with a polychoric matrix, Bartlett test of sphericity and Kaiser-Meyer-Olkin Measure of Sampling Adequacy.[20] Exploratory item factor analysis using principal factor extraction was conducted on the correlation matrix. Unrotated and oblique rotations were performed, looking for factors with an Eigenvalue greater than one and items with loading of >0.2. Confirmatory factor analysis (CFA) with a generalised structural equation model (gSEM) was carried out to compare the one factor model, a two-factor model of positive vs negative items and a three-factor model based on the underlying domains of the DAP scale. As estimates of model fit are not available in STATA for gSEM models, we compared model fit with the AIC and BIC where lower AIC/BIC values indicate better fit.[21]

Construct validity (hypothesis testing)

To test construct validity by hypothesis testing, we hypothesised that women with a higher DAP score would be more likely to use contraception using baseline data. We created a logistic regression model with self-reported use of any contraception (including all modern contraceptive methods, withdrawal and fertility awareness) in the last 30 days as a binary outcome variable (among women who reported sex with a male partner in the last 30 days) and DAP score as the exposure variable.

Criterion validity

We assessed the criterion validity of the DAP via its predictive validity with regards to pregnancy occurring within one year of completion of the measure at baseline. We generated a binary yes/no variable for whether a woman had had any pregnancies during the 12month follow up period, as reported at each of the quarterly surveys, regardless of pregnancy outcome (eg miscarriage, termination, ectopic, ongoing or other) or timing. This was used as the binary outcome and DAP score as the predictor in a logistic regression model.

Reliability (internal consistency)

Internal consistency reliability was assessed with the separation reliability coefficient (Person Separation Index) calculated from fitting the item responses to the PCM),[18] the Cronbach's α (>0.7) and by calculating item-rest correlations (to check they were all positive and >0.2).[22, 23]

For peer review only - http://bmjopen.bmj.com/site/about/guidelines.xhtml

Reliability (test-retest)

We assessed test-retest reliability with a two-way mixed effects intraclass correlation coefficient (ICC) on women who completed a two-week retest, looking for an ICC >0.8.[24]

Differential item functioning

We evaluated differential item functioning (DIF) by age (15-24/25-34/35+), ethnicity (white/non-white), relationship group (no relationship/in a relationship not married/married), and number of children (0/1/2/3+) by introducing item-by-trait terms into the IRT measurement model and examining the estimates to see how much the location differed by trait. We used a total logit difference between groups of 0.6 logits as a meaningful difference.[25] Based on findings in the US we hypothesised that there would be DIF on item 5 (Becoming pregnant in the next 3 months would bring me closer to my main partner) by age, and number of children, but not for ethnicity, and for there to be no DIF on any other items.

Exploratory analysis of factors associated with DAP score

We developed explanatory IRT models for ethnicity, relationship status, age and number of children. We expected no difference in DAP scores for ethnicity and for it to be easier to endorse items (i.e. have a higher desire to avoid pregnancy) if women were not in a relationship, were under 24 or had no children. We checked this by fitting bivariable and multivariable linear regression models.

Analyses were conducted in ACER ConQuest version 5.9.0 and Stata version 17.

Ethical approval and funding

We received ethical approval from the UCL Research Ethics Committee for both the development and testing of the survey (ref 3974.002) and for the online survey with 12month follow up (ref 3974.003). The study was funded by an NIHR Advanced Fellowship held by JH (PDF-2017-10-021).

Data availability statement

The dataset is available in the UCL Research Data Repository [26].

For peer review only

RESULTS

Participant characteristics

Figure 1 shows how the cohort of 994 was reached from a total of 1337 records that were created in the online survey software.

<<<<<<<<<<< Figure 1 around here >>>>>>>>>>>>>>>>>>

Women were aged 15-50 years (median 31, IQR 23-36, mean 29.7), 83.9% were white, 81.6% described themselves as heterosexual and most were married (48.2%) or in a relationship (34.0%) (Table 1). A quarter of the women in the cohort had completed school education (i.e. to age 18), 39.1% were educated to undergraduate level and 31.1% had postgraduate or professional qualifications. More than half (56.7%) had at least one child. The success of the social media recruitment is shown in the distribution of women recruited from across the UK.

		Freq.	Percent			Freq.	Percent
Age group	15-19	139	14	UK region of residence	Channel Islands	1	0.1
	20-24	143	14.4		East England	8	0.8
	25-29	139	14		East Midlands	80	8.05
	30-34	224	22.5		East of England	78	7.85
	35-39	209	21		Greater London	254	25.55
	40-44	78	7.9		North East	66	6.64
	45+	23	2.3		North West	81	8.15
	Missing	39	3.9		Northern Ireland	16	1.61
Relationship	Not in a relationship	152	15.3		Scotland	83	8.35
	In a relationship, but not married	338	34.0		South East	123	12.37
	Married	479	48.2		South West	94	9.46
	Other	1	0.1		Wales	35	3.52
	Missing	24	2.4		West Midlands	60	6.04
Race/ Ethnicity	White	834	83.9		Missing	15	1.51
	Mixed	26	2.6	Number of	0	430	43.3
	Asian	69	6.9		1	208	20.9

For peer review only - http://bmjopen.bmj.com/site/about/guidelines.xhtml

	Black	24	2.4	**children**	2	233	23.4
	Other	9	0.9		3+	78	7.9
	Prefer not to say/ Missing	32	3.2		Missing	45	4.5
**Completed level of education**	School	247	24.9	**Sexuality**	Heterosexual	811	81.6
	Undergraduate	389	39.1		Not heterosexual	146	14.7
	Postgraduate	309	31.1		Prefer not to say/ Missing	37	3.7
	Other	18	1.8				
	Missing	31	3.1				

Table 1 Sociodemographic details of the whole sample at baseline

The women who completed the two-week re-test (n=152) and those who completed follow up at 12 months (over 90% of eligible women) were not significantly different by age, relationship status, ethnicity or number of children than those who were not randomly selected into the re-test sample than the baseline sample (data not shown).

Cognitive Interviews

The cognitive interviews demonstrated that the DAP was easily understood by women in England. We made minor changes to the syntax that made the flow better when read by women in the UK. The main changes we made were to the wording of the definition of a 'partner'. First, we added a sentence to say 'The next question asks about your partner', tested several iterations of wording for describing what could be considered as a 'partner' and added wording to help women without a current partner to decide how to answer this question. We rephrased 'sexual relations', which women understood to be penetrative sex only, as 'physically intimate'. The wording of the UK DAP is in supplementary file S1.

Acceptability, endorsement and targeting

The full range of DAP scores (0-4) was captured; there was a negative skew, as shown in Figure 2, with more women having higher levels of desire to avoid pregnancy. The mean score was 2.53, standard deviation 1.05, median 2.71 and inter-quartile range 1.86-3.36. Although all questions were optional in our online survey, there was over 95% completion for every DAP item and very little missing data. No item category had >80% endorsement (Supplementary file S2).

<<<<<<<<<<< Figure 2 around here >>>>>>>>>>>>>>>>>>

For peer review only

For peer review only - http://bmjopen.bmj.com/site/about/guidelines.xhtml

The Wright map (Supplementary File S3) showed the full range of participant locations with item thresholds falling at all levels of participants' pregnancy preferences, demonstrating good targeting and that the items captured the full range of the construct.

Construct validity (structural validity)

Item fit to model

Most items fit the partial credit model, based on the weighted mean square statistic being between 0.76-1.33, but five items did not (Supplementary file S4). It was noted that the two items with misfit indicating greater deviance than expected due to higher discrimination than the model predicted (items with a weighted mean fit of <0.75) were both positively worded items in domain one, cognitive preferences. Conversely, the three items with poorer fit due to lower discrimination (items with a weighted mean fit of >1.33) were in domain three; anticipated practical consequences, and two of them were negatively worded. Item 5, about the partner, had the poorest fit to model, consistent with the original development.[7] Repeating the item-fit analysis with either a two factor (positive v negative items) or three factor (domains) model reduced the item misfit.

Monotonicity was confirmed i.e. that average DAP scores increased among those endorsing each increasing response category on each individual item, indicating excellent internal structure. Item characteristic curves confirmed that the responses to each item's categories were ordered.

Factor Analysis

Tests confirmed the split datasets (n=497 in each) were suitable for factor analysis (Bartlett test of sphericity (p<0.001) and Kaiser-Meyer-Olkin Measure of Sampling Adequacy (0.960)). EFA using principal factor extraction with no rotation conducted on the polychoric factor matrix suggested a one factor model with an Eigenvalue of 9.84 (LR test <0.001) and where this factor explained 91% of the variability in responses. All items strongly loaded on to this factor (loadings >0.7 for all except item 5 which was 0.51). Oblique rotation did not improve model fit.

Confirmatory factor analysis was conducted the one factor model proposed by the EFA and the two and three factor models suggested by the IRT analysis. There was very little difference in the AIC or BIC between the models; the two-factor (positive v negative) was the best fit and three-factor model

For peer review only - http://bmjopen.bmj.com/site/about/guidelines.xhtml

(domains) was almost identical, followed by the one factor model, however, the differences were small.

Construct validity (hypothesis testing)

As per our hypothesis, among women who had had sex with a male partner in the last 30 days, those with higher DAP scores (greater desire to avoid pregnancy) were significantly more likely to report contraception use in the last 30 days (OR 3.20 95%CI 2.58, 3.98). A predicted 29.5% of women with a DAP score of zero were using contraception (95%CI 20.6%, 38.5%), compared to 98% (95%CI 96.6%, 98.9%) for women with a DAP score of four.

Criterion validity

Over 90% of women eligible for follow up at 12months completed the survey and 139 women experienced at least one pregnancy during the year of the cohort. For every one-point increase in DAP score there were 0.22 the odds of becoming pregnant within 12months (95%CI 0.17, 0.28). As shown in Figure 3, women with a DAP score of zero had an 80% (95%CI 72.1%, 86.7%) probability of becoming pregnant within a year, compared to 0.89% (95%CI 0.36%, 1.44%) with a score of four.

<<<<<<<<<<< Figure 3 around here >>>>>>>>>>>>>>>>>>

Reliability (Internal consistency)

The Person Separation Reliability coefficient was 0.91 and Cronbach's α was 0.96, both extremely high. All inter-item correlations were positive and all item-total correlations were positive and greater than 0.2. Item 5, "Becoming pregnant in the next 3 months would bring me closer to my (main) partner" had the lowest item-test and item-rest correlations (0.506 and 0.441 respectively) and the scale's internal consistency was not reduced (i.e., α remained 0.96) when excluding it.

Reliability (test-retest)

The intraclass correlation coefficient was 0.95 (95%CI 0.92, 0.97) indicating excellent stability over the two-week test-retest period.

Differential Item Functioning

As expected, we found evidence of DIF on item 5 (Becoming pregnant in the next 3 months would bring me closer to my main partner) by age, number of children, and relationship group. For example, compared to participants aged 25-34 years with otherwise similar DAP scores, those aged 15-24 were more likely to agree to the statement. There was also evidence of DIF on item 13 (If I had

For peer review only

For peer review only - http://bmjopen.bmj.com/site/about/guidelines.xhtml

a baby in the next year, it would be hard for me to manage raising the child) by age, number of children and relationship group, with younger women more likely to agree to the statement than older women with otherwise similar DAP scores and those with more children or in a relationship less likely to agree with the statement than those with fewer children or not in a relationship.

Exploratory analysis of factors associated with DAP score

Both the eIRM and linear regressions showed that there was no significant difference in overall DAP score by ethnicity (β 0.18 95%CI -0.02, 0.38) but there were differences by age, relationship status, number of children and education level. A multivariable linear regression model was created; ethnicity remained non-significant and was excluded from the model; all other variables remained statistically significant, as shown in Table 2.

For peer review only

Variable		Coefficient	95% CI		p-value
**Relationship status**	**Baseline - married**				
	In a relationship, not married	0.48	0.30	0.65	<0.001
	Not in a relationship	0.77	0.53	1.00	
**Age**	**Baseline 15-24**				
	25	-0.63	-0.84	-0.42	<0.001
	35	-0.50	-0.75	-0.26	
**Number of children**	**Baseline - 0**				
	1	-0.22	-0.42	-0.01	<0.001
	2	0.63	0.40	0.85	
	3	0.81	0.53	1.09	
**Level of education**	**Baseline - School**				
	Undergraduate	-0.17	-0.35	0.00	0.043
	Postgraduate	-0.04	-0.23	0.16	
	Other	0.32	-0.13	0.77	

Table 2 Multivariable linear regression of characteristics associated with DAP score

DISCUSSION

The DAP scale generally performed well in the UK population. Cognitive interviews and the high completion rates showed good understanding and acceptability to women. The DAP items captured the full range of participants' pregnancy preference levels, and the scale showed good targeting and acceptable levels of endorsement across item response options. In terms of construct (structural)

For peer review only - http://bmjopen.bmj.com/site/about/guidelines.xhtml

validity, mean scores increased by each item's increasing categories and the item categories' peaks were ordered on the item characteristic curves. Our construct validity hypothesis test confirmed that for every one-point increase in DAP score, women were three times as likely to be using contraception. In addition, the DAP score was highly predictive of pregnancy, with 80% of those with a DAP score of zero likely to become pregnant within a year, compared to <1% of those with a DAP score of four. Reliability, both in terms of internal consistency and test-retest, was excellent.

However, there were a few areas where our expectations were not met. Based on the item-fit estimates to the PCM, there was some suggestion that the DAP scale may not be unidimensional. While EFA suggested a single factor model, CFA found that a two-factor model (i.e. treating positively and negatively worded items as separate factors) fitted marginally better than a three-factor model (i.e. treating each of the DAP's conceptual domains as separate factors), which fitted marginally better than one factor. From a psychometric perspective this suggests that there may be different dimensions within the DAP scale, rather than it being unidimensional. Nevertheless, the differences in AIC/BIC were very small and we are limited by the lack of other goodness of fit statistics available in STATA, so we cannot confidently recommend an improved structure. Notably, when fitting the DAP items to a multidimensional PCM treating positively and negatively worded items as different dimensions, all items other than item 5 met criteria for item fit. The DAP is a new measure; this is the first evaluation outside the USA to be published though we are aware of other studies underway. We suggest that these studies conduct further analysis of the factor structure and dimensionality of the DAP based on our findings. Regardless the DAP appears to be functioning well as a measure, predicting pregnancy and passing the standard CTT-based tests.

There were similarities between the UK and USA in that the item that was hardest to endorse was the same in both settings (Item 9: "It would be the end of the world for me to have a baby in the next year")[7]. In the UK sample however, women found it easier to agree with the items in the conceptual domain of 'anticipated practical consequences' (items 5, 12, 13 and 14) meaning they felt that having a baby in the next year would have a greater impact on their lives, in terms of practical consequences, than the women in the US sample. For example, on item 12 (I would feel a loss of freedom if I had a baby in the next year) 35% of women in the US sample agreed or strongly agreed, compared to 64% in the UK sample. Comparing these items between the two samples suggests a real difference in how different groups of women think about the effects that childbearing would have on their lives under their current circumstances. This may be explained in part by important differences between the populations studied in the UK and USA. The US sample was recruited from reproductive and primary health facilities and targeted women 'at risk' of pregnancy. Women in the US study were younger (41% <25 compared to 28%), less likely to be white (16% v 84%), married

For peer review only - http://bmjopen.bmj.com/site/about/guidelines.xhtml

(26% vs 48%), or to have completed college-level education (10% vs 70%) and were slightly more likely to already have a child (61% vs 57%).[7] These are all factors which are known to influence pregnancy preferences and translated to a different distribution of the DAP score, with women in the UK sample expressing a higher desire to avoid pregnancy (averaged raw scores: USA mean 2.2, SD 1.1 vs UK mean 2.5, SD 1.1).

Our analyses pointed to relatively poor psychometric performance of item 5 (pregnancy would bring me closer to my main partner) in several ways, consistent with the USA study.[7] In our cognitive interviews, respondents without a partner found the item difficult to respond to; some opted for the neutral 'neither agree nor disagree' response while others selected 'strongly disagree' as they had no partner to become closer to. Item 5 was the item we made the most changes to in terms of the wording, much of which was aimed at how women without a partner should think about this question. Psychometrically, the item did not fit the PCM, and illustrated notable DIF by several sociodemographic variables. Although partnership factors are a key component shaping an individual's pregnancy preferences, it is likely that people's perceptions of how a pregnancy would affect a relationship could be reflected in their responses to other DAP items. Removal of this item should be considered, and as our analysis showed, would not reduce the high internal consistency of the scale.

Multivariable analysis has shown that women's desire to avoid pregnancy is associated with age, relationship status and number of children, which is consistent with the wider literature on the determinants of pregnancy intention[8, 27-32]. Future analyses could consider how DAP changes over time and as external factors, such as relationship status and education level, change.

Strengths and Limitations

This is a large study with data collected from women across the UK in the community, rather than from healthcare settings. We captured the full spectrum of desire to avoid pregnancy and are the first to assess the properties of the DAP scale using IRT and CTT methods outside the USA. We conducted a test-retest analysis and, uniquely, examined the predictive validity of the DAP by looking at pregnancy over the subsequent year.

Our recruitment strategy may have contributed to the sample being more educated than average, as we used a college and university as recruitment sites. Furthermore, social media recruitment can result in an over-representation of young white women and disadvantages those without internet access.[33, 34] While the proportion of women from a black or ethnic minority background was commensurate with the general population, in absolute terms this was only 160 women, just 15 of

For peer review only - http://bmjopen.bmj.com/site/about/guidelines.xhtml

whom experienced a pregnancy during this study. Further work should be conducted in non-white women. In this study participants self-reported that they were female, and we did not ask about gender identity; it will be important to understand how the DAP works for people of all genders.

CONCLUSION

The UK DAP is a valid and reliable measure of desire to avoid pregnancy and a useful tool for research. CTT tests showed that the measure was highly valid and reliable based on established standards. While not all pre-set criteria were met for the IRT tests, the criteria were met when considering the possibility of multidimensionality. Future evaluations of the DAP should consider removal of item 5 and examine different factor structures in light of our findings. We are the first to show that the DAP score is highly predictive of pregnancy. This is of particular use to those attempting to recruit a preconception cohort or, conversely, women who are unlikely to become pregnant, for example for a pharmacological study. The DAP may also have a potential clinical use, to guide preconception or contraception care. In order to be practical, consideration should be given to a shortened version to make it more amenable for use in busy, time-constrained clinical settings.

For peer review only

For peer review only - http://bmjopen.bmj.com/site/about/guidelines.xhtml

415 **Acknowledgements**

416 We would like to thank Dr. Mark Wilson for his support around item response theory-based analyses
417 and Ms Anasztazia Gubijev who supported the follow up of the cohort. We would also like to thank
418 the project steering committee (Prof Judith Stephenson, Prof Anna David, Dr Sue Mann, Dr Sarah
419 Crozier, Dr Natalie Edelman, Dr Anita Coutinho and Mr James Harris) for their input on the data
420 cleaning decisions and overall guidance.

421 **Funding statement**

422 The study was funded by an NIHR Advanced Fellowship held by JH (PDF-2017-10-021).

423 **Competing interests**

424 The authors declare that they have no conflicts of interest.

425 **Author contributions**

426 Jennifer Hall: Conceptualisation, Funding acquisition, Methodology, Investigation, Data Curation,
427 Formal analysis, Writing – Original Draft, Writing – Review and Editing, Project administration.
428 Geraldine Barrett: Conceptualisation, Methodology, Investigation, Writing – Review and Editing.
429 Corinne Rocca: Conceptualisation, Methodology, Formal analysis, Writing – Review and Editing.

430

For peer review only

For peer review only - http://bmjopen.bmj.com/site/about/guidelines.xhtml

References

1. Aiken, A.R., et al., *Rethinking the Pregnancy Planning Paradigm: Unintended Conceptions or Unrepresentative Concepts?* Perspect Sex Reprod Health, 2016. **48**(3): p. 147-51.
2. Bachrach CA and Newcomer S, *Intended pregnancies and unintended pregnancies: distinct categories or opposite ends of a continuum?* Fam Plann Perspect, 1999. **31**(5): p. 251-2.
3. Barrett G and Wellings K, *Understanding Pregnancy Intentions: A Problem in Evidence Everywhere.* Family Planning Perspectives, 2000. **32**(4): p. 194.
4. Mumford, S.L., et al., *Pregnancy intentions-a complex construct and call for new measures.* Fertil Steril, 2016. **106**(6): p. 1453-1462.
5. Santelli, J., et al., *The measurement and meaning of unintended pregnancy.* Perspect Sex Reprod Health, 2003. **35**(2): p. 94-101.
6. Santelli, J.S., et al., *Toward a multidimensional measure of pregnancy intentions: evidence from the United States.* Stud Fam Plann, 2009. **40**(2): p. 87-100.
7. Rocca, C.H., et al., *Psychometric Evaluation of an Instrument to Measure Prospective Pregnancy Preferences: The Desire to Avoid Pregnancy Scale.* Med Care, 2019. **57**(2): p. 152-158.
8. Wellings, K., et al., *The prevalence of unplanned pregnancy and associated factors in Britain: findings from the third National Survey of Sexual Attitudes and Lifestyles (Natsal-3).* Lancet, 2013. **382**(9907): p. 1807-16.
9. Upadhyay, U.D., et al., *Development and validation of a reproductive autonomy scale.* Stud Fam Plann, 2014. **45**(1): p. 19-41.
10. Government Statistical Service, *Harmonisation Strategy*, O.f.N. Statistics, Editor.: UK.
11. Natsal, *Natsal 3 Questionnaire*. 2012.
12. Harris, P.A., et al., *The REDCap consortium: Building an international community of software platform partners.* J Biomed Inform, 2019. **95**: p. 103208.
13. Harris, P.A., et al., *Research electronic data capture (REDCap)--a metadata-driven methodology and workflow process for providing translational research informatics support.* J Biomed Inform, 2009. **42**(2): p. 377-81.
14. Anthoine, E., et al., *Sample size used to validate a scale: a review of publications on newly-developed patient reported outcomes measures.* Health Qual Life Outcomes, 2014. **12**: p. 176.
15. Kline PA, *Handbook of Test Construction: Introduction to Psychometric Design*. 1986, London, UK: Methuen.
16. Kutscher, T., M. Eid, and C. Crayen, *Sample Size Requirements for Applying Mixed Polytomous Item Response Models: Results of a Monte Carlo Simulation Study.* Front Psychol, 2019. **10**: p. 2494.
17. Hall, J.A., et al., *Reassessing pregnancy intention and its relation to maternal, perinatal and neonatal outcomes in a low-income setting: A cohort study.* PLoS One, 2018. **13**(10): p. e0205487.
18. Wright BD and Masters GN, *Rating Scale Analysis (Rasch Measurement Series)*. 1981, Chicago, IL: MESA Press.
19. Smith, W., et al., *Exploring the factor structure of the Health of the Nation Outcomes Scale (HoNOS) in a sample of patients with schizophrenia, schizotypal and delusional disorders.* Health Qual Life Outcomes, 2017. **15**(1): p. 141.
20. Dziuban CD, *When is a correlation matrix appropriate for factor analysis? Some decision rules.* Psychological Bulletin, 1974. **81**(6): p. 358-361.
21. Raftery AE, *Bayesian model selection in social research.* Sociological Methodology, 1995. **25**: p. 111-163.
22. Cronbach LJ, *Coefficient alpha and the internal structure of tests.* Psychometrika 1951. **16**: p. 297-334.

For peer review only

For peer review only - http://bmjopen.bmj.com/site/about/guidelines.xhtml

23. Scientific Advisory Committee of the Medical Outcomes Trust, *Assessing health status and quality-of-life instruments: attributes and review criteria.* Qual Life Res, 2002(11): p. 193-205.
24. Qin, S., et al., *Assessing test-retest reliability of patient-reported outcome measures using intraclass correlation coefficients: recommendations for selecting and documenting the analytical formula.* Qual Life Res, 2019. **28**(4): p. 1029-1033.
25. Paek I, *Investigations of Differential Item Functioning: Comparisons Among Approaches, and Extension to a Multidimensional Context*. 2002, Berkeley: University of California.
[dataset] 26. Hall, J., *Evaluation of the UK version of the Desire to Avoid Pregnancy scale*. 2022: UCL Research Data Repository. https://rdr.ucl.ac.uk/articles/dataset/Evaluation_of_the_UK_version_of_the_Desire_to_Avoid_Pregnancy_scale/20195081
27. Hall, J.A., et al., *Prevalence and Determinants of Unintended Pregnancy in Mchinji District, Malawi; Using a Conceptual Hierarchy to Inform Analysis.* PLoS One, 2016. **11**(10): p. e0165621.
28. Hayford, S.R. and K.B. Guzzo, *Age, relationship status, and the planning status of births.* Demographic Research, 2010. **23**: p. 365-398.
29. Huynh, S.T., et al., *Prevalence of and factors associated with unplanned pregnancy among women in Koshu, Japan: cross-sectional evidence from Project Koshu, 2011-2016.* BMC Pregnancy Childbirth, 2020. **20**(1): p. 397.
30. Kost, K. and L. Lindberg, *Pregnancy Intentions, Maternal Behaviors, and Infant Health: Investigating Relationships With New Measures and Propensity Score Analysis.* Demography, 2015. **52**(1): p. 83-111.
31. Lang, A.Y., et al., *Opportunities for enhancing pregnancy planning and preconception health behaviours of Australian women.* Women Birth, 2021. **34**(2): p. e153-e161.
32. Lindberg, L., et al., *Pregnancy Intentions and Maternal and Child Health: An Analysis of Longitudinal Data in Oklahoma.* Maternal and Child Health Journal, 2015. **19**(5): p. 1087-1096.
33. Benedict, C., et al., *Recruitment via social media: advantages and potential biases.* Digit Health, 2019. **5**: p. 2055207619867223.
34. Whitaker, C., S. Stevelink, and N. Fear, *The Use of Facebook in Recruiting Participants for Health Research Purposes: A Systematic Review.* J Med Internet Res, 2017. **19**(8): p. e290.

For peer review only

For peer review only - http://bmjopen.bmj.com/site/about/guidelines.xhtml

Figure Captions

Figure 1 Flowchart of recruitment and inclusion of participants

Figure 2 Frequency distribution of DAP score at baseline

Figure 3 Probability of pregnancy within one year as predicted by DAP score

For peer review only

For peer review only - http://bmjopen.bmj.com/site/about/guidelines.xhtml

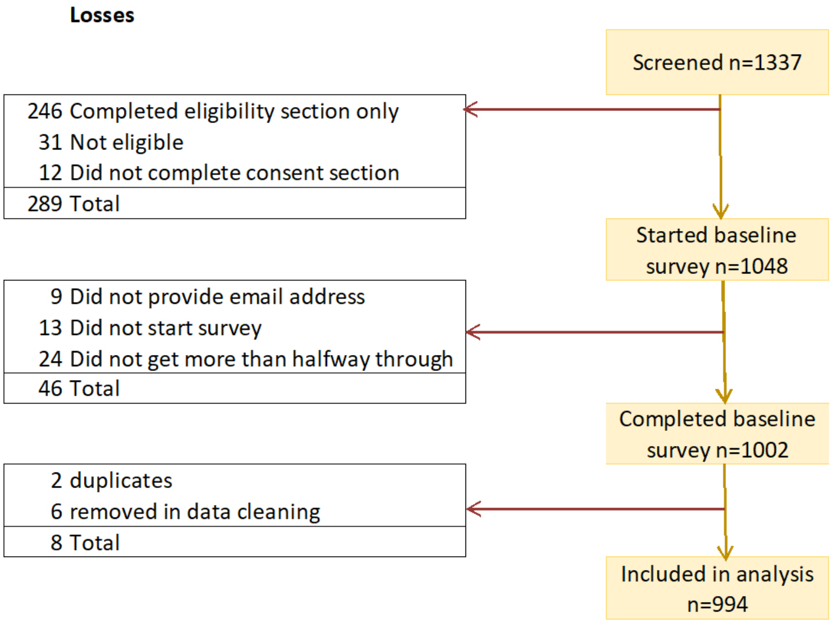

Flow chart of recruitment and inclusion of participants

203x126mm (144 x 144 DPI)

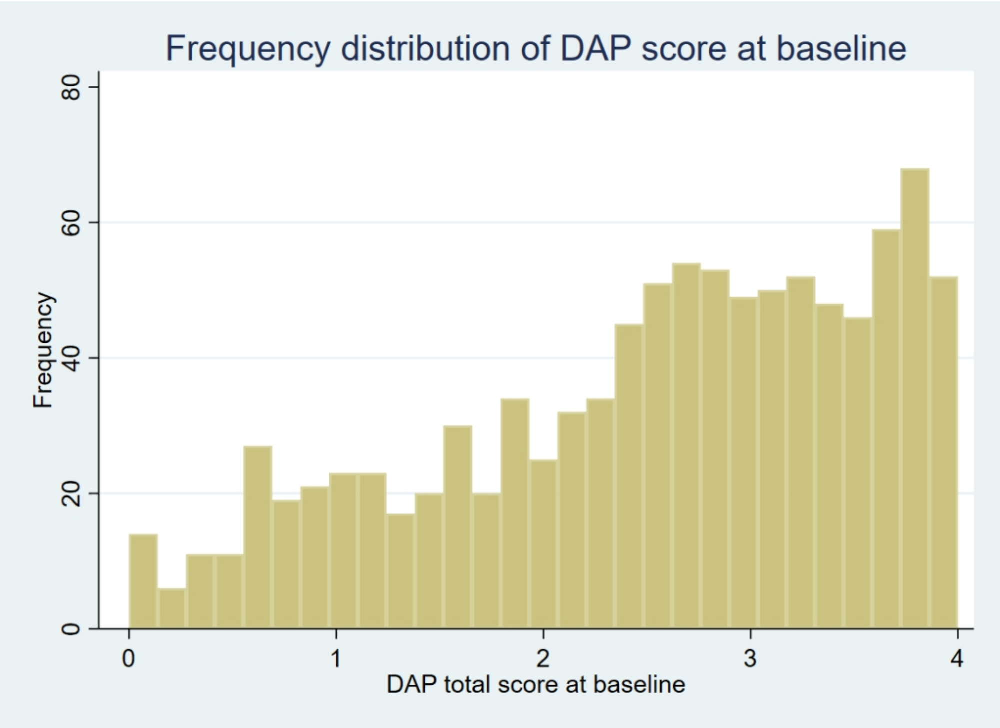

Figure 2 Frequency distribution of DAP score at baseline

411x299mm (72 x 72 DPI)

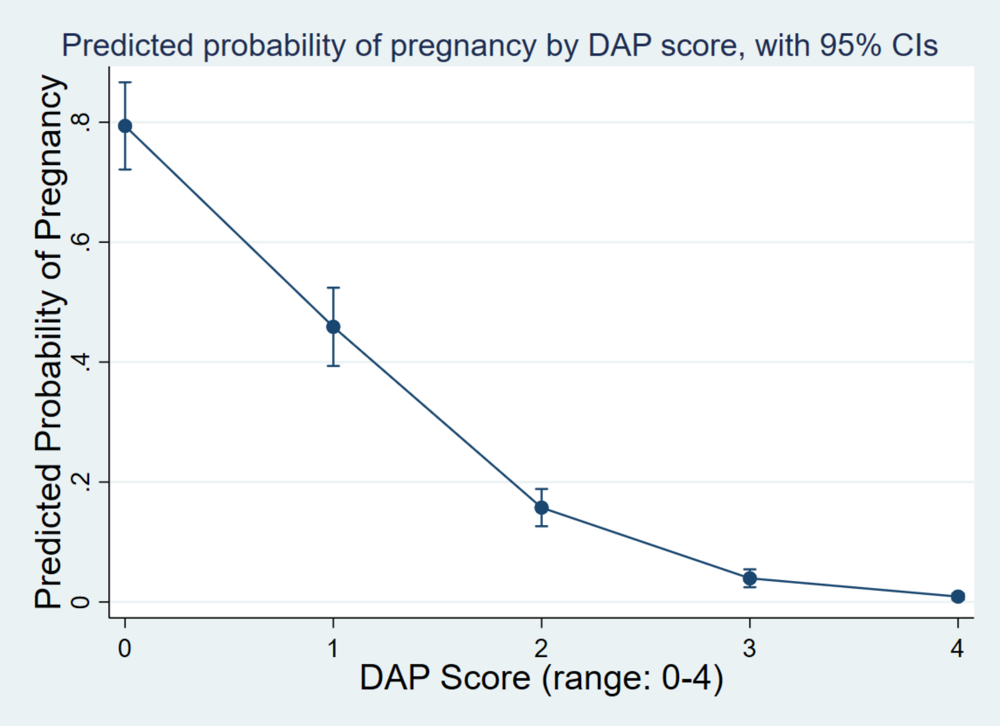

Probability of pregnancy within one year as predicted by DAP score

403x293mm (72 x 72 DPI)

Desire to Avoid Pregnancy Scale – wording of UK version

We would like to ask you about your current thoughts and feelings about the idea of becoming pregnant in the next three months and having a baby in the next year.

We know that women have very different thoughts and feelings about pregnancy, and having a baby, that can change over time.

Please remember that there are no right or wrong answers. For each question, choose one answer that seems right at this point in your life.

The following questions ask about your thoughts and feelings about the idea of becoming PREGNANT IN THE NEXT THREE MONTHS.

Even if you do not think you can become pregnant for partner or physical reasons, please imagine how you feel about BECOMING PREGNANT in the next 3 months.

(5 point Likert scale from Strongly agree to Strongly disagree)

I wouldn't mind if I became pregnant in the next 3 months. (Item 1)

It would be a good thing for me if I became pregnant in the next 3 months. (Item 2)

Thinking about becoming pregnant in the next 3 months makes me feel unhappy. (Item 3)

Thinking about becoming pregnant in the next 3 months makes me feel excited. (Item 4)

The next questions ask you to think about your main partner. By main partner, we mean the romantic partner that is the most serious to you.

If you don't have a romantic partner, please think about the last person with whom you were physically intimate (anything from kissing and cuddling to sex) or think of a person you know who you would consider being physically intimate with.

Becoming pregnant in the next 3 months would bring me closer to my main partner. (Item 5)

The following questions ask about your thoughts and feelings about the idea of having a BABY IN THE NEXT YEAR.

Even if you do not think you can have a baby for partner or physical reasons, please imagine how you feel about HAVING A BABY in the next year.

(5 point Likert scale from Strongly agree to Strongly disagree)

I want to have a baby within the next year. (Item 6)

If I had a baby in the next year, it would be bad for my life. (Item 7)

It would be a positive addition to my life to have a baby in the next year. (Item 8)

It would be the end of the world for me to have a baby in the next year. (Item 9)

Thinking about having a baby within the next year makes me smile. (Item 10)

Thinking about having a baby within the next year makes me feel stressed out. (Item 11)

I would feel a loss of freedom if I had a baby in the next year. (Item 12)

For peer review only

If I had a baby in the next year, it would be hard for me to manage raising the child. (Item 13)

I would worry that having a baby in the next year would make it harder for me to achieve other things in my life. (Item 14)

For peer review only

Supplementary file 2

Table of responses to DAP questions showing endorsement and missing data

Item	Wording	Strongly agree	Agree	Neither agree nor disagree	Disagree	Strongly disagree	Missing
1	I wouldn't mind if I became pregnant in the next 3 months	11.3	12.5	6	23.4	46.8	0
2	It would be a good thing for me if I became pregnant in the next 3 months	8.6	5.7	9.2	23.8	52.6	0.1
3	Thinking about becoming pregnant in the next 3 months makes me feel unhappy	12.8	17.4	19.3	23.6	26.8	0.1
4	Thinking about becoming pregnant in the next 3 months makes me feel excited	9.8	12.1	13.3	26.5	38.3	0.1
5	Becoming pregnant in the next 3 months would bring me closer to my main partner	7.4	20.2	26.4	26.4	19.4	0.2
6	I want to have a baby within the next year	11.3	8.4	8.7	26.4	45.3	0.1
7	If I had a baby in the next year, it would be bad for my life	11	21.3	17.8	23	26.8	0.1
8	It would be a positive addition to my life to have a baby in the next year	10.2	18.8	20.4	26.1	24.5	0
9	It would be the end of the world for me to have a baby in the next year	31.7	28.8	15.3	11	13.2	0.1
10	Thinking about having a baby within the next year makes me smile	13.3	18.8	13.5	31.7	22.7	0
11	Thinking about having a baby within the next year makes me feel stressed out	5	12.4	12	40.8	29.7	0.1
12	I would feel a loss of freedom if I had a baby in the next year	7.3	18.7	10.1	31.6	32.2	0.1
13	If I had a baby in the next year, it would be hard for me to manage raising the child	6.3	23.2	12.1	30.3	28.1	0
14	I would worry that having a baby in the next year would make it harder for me to achieve other things in my life	3.8	13.2	8.1	32.6	42.2	0.1

For peer review only

```
                                        Generalised-Item Thresholds
------------------------------------------------------------------------------------
  6                                         |
                          XXXXXXXXXXXXXXXXXX|
                                            |
  5                                         |
                                 XXXXXXXXXXX|
                                            |
  4                                         |
                          XXXXXXXXXXXXXXXXXX|
                                            |
                         XXXXXXXXXXXXXXXXXXX|
  3                                        X|9.4
                            XXXXXXXXXXXXXXXX|
                           XXXXXXXXXXXXXXXXX|5.4
                            XXXXXXXXXXXXXXXX|10.4
  2                    XXXXXXXXXXXXXXXXXXXXX|3.4 7.4 8.4 9.3
                            XXXXXXXXXXXXXXXX|11.4 13.4
                XXXXXXXXXXXXXXXXXXXXXXXXXXXX|12.4
                XXXXXXXXXXXXXXXXXXXXXXXXXXXX|4.4 9.2
                 XXXXXXXXXXXXXXXXXXXXXXXXXXX|14.4
  1            XXXXXXXXXXXXXXXXXXXXXXXXXXXXX|1.4 5.3 6.4
             XXXXXXXXXXXXXXXXXXXXXXXXXXXXXXX|3.3 7.3 8.3
    XXXXXXXXXXXXXXXXXXXXXXXXXXXXXXXXXXXXXXXX|2.4 10.3 13.3
            XXXXXXXXXXXXXXXXXXXXXXXXXXXXXXXX|
  0         XXXXXXXXXXXXXXXXXXXXXXXXXXXXXXXX|4.3 7.2 10.2 12.3
                  XXXXXXXXXXXXXXXXXXXXXXXXXX|1.3 3.2 6.3 8.2 9.1 11.3 13.2
                       XXXXXXXXXXXXXXXXXXXXX|1.2 5.2 12.2 14.3
                            XXXXXXXXXXXXXXXX|2.3 4.2 6.2
 -1                        XXXXXXXXXXXXXXXXX|11.2 14.2
                               XXXXXXXXXXXXX|2.2
                                XXXXXXXXXXXX|1.1 3.1 6.1 10.1
                                 XXXXXXXXXXX|2.1 4.1 7.1
                             XXXXXXXXXXXXXXX|8.1
 -2                                       XX|
                                        XXXX|5.1 12.1
                                         XXX|11.1 13.1
                                         XXX|
 -3                                         |14.1
                                           X|
                                            |
                                          XX|
                                            |
 -4                                         |
                                         XXX|
                                            |
 -5                                         |
                                       XXXXX|
 -6                                         |
====================================================================================
Each 'X' represents 1.8 cases
The labels for thresholds show the levels of item, and category, respectively
====================================================================================
```

Supplementary file S4

DAP items by domain with item fit and location estimate

			Item fit	Item location	
**Item**	**Cognitive Desires and Preferences**	**Wording**	**Weighted MNSQ**	**UK Estimate**	**USA Estimate**
1	I wouldn't mind if I became pregnant in the next 3 months	positive	0.63	-0.304	-0.60
2	It would be a good thing for me if I became pregnant in the next 3 months	positive	0.7	-0.679	-0.92
6	I want to have a baby within the next year	positive	0.71	-0.349	-0.61
7	If I had a baby in the next year, it would be bad for my life	negative	0.76	0.269	0.69
8	It would be a positive addition to my life to have a baby in the next year	positive	0.65	0.238	0
9	It would be the end of the world for me to have a baby in the next year	negative	1.07	**1.593**	**0.93**
	**Affective Feelings and Attitudes**				
3	Thinking about becoming pregnant in the next 3 months makes me feel unhappy	negative	0.89	0.303	0.28
4	Thinking about becoming pregnant in the next 3 months makes me feel excited	positive	0.82	-0.219	-0.36
10	Thinking about having a baby within the next year makes me smile	positive	0.68	0.377	0.24
11	Thinking about having a baby within the next year makes me feel stressed out	negative	1.11	-0.453	-0.16
	**Anticipated Practical Consequences**				
5	Becoming pregnant in the next 3 months would bring me closer to my main partner	positive	2.21	0.261	0
12	I would feel a loss of freedom if I had a baby in the next year	negative	1.48	-0.177	0.42
13	If I had a baby in the next year, it would be hard for me to manage raising the child	negative	1.28	-0.071	0.33
14	I would worry that having a baby in the next year would make it harder for me to achieve other things in my life	negative	1.47	**-0.792***	-0.23

Hall et al. Evaluation of the Desire to Avoid Pregnancy Scale in the United Kingdom: a psychometric analysis including predictive validity. CHECKLIST compatibility

In the absence of the availability of a self-complete checklist document, we list here the how we have met the *COSMIN reporting guidelines for studies on measurement properties of patient reported outcome measures*. Our responses below should be read in conjunction with the pdf of the checklist: https://www.cosmin.nl/wp-content/uploads/COSMIN-reporting-guideline_1.pdf

T1 Yes

T2 Yes

T3 Yes

A1 Yes

A2 Yes

A3 Yes

A4 Yes

A5 Yes

A6 Yes

A7 Yes

I1 Yes

I2 Yes

I3 Yes

I4 Yes

I5 Yes

I6 Yes

GM1 Yes

GM1 Yes

GM3 Yes

GM4 Yes

GM5 Yes

GM6 Yes

For peer review only

For peer review only - http://bmjopen.bmj.com/site/about/guidelines.xhtml

GM7	NA
GM8	NA
GR1	Yes
GR2	Yes
GR3	Yes
D1	Yes
D2	Yes
D3	Yes
D4	Yes
D5	Yes
D6	Yes
C1	Yes
O1	Yes
CV1	NA
CV2	NA
CV3	Yes
CV4	NA
CV5	Yes
CV6	NA
CV7	Yes
SV1	Yes
SV2	Yes
IC1	Yes

For peer review only

For peer review only - http://bmjopen.bmj.com/site/about/guidelines.xhtml

IC2	Yes
IC3	NA
CCV1	NA
CCV2	NA
CCV3	NA
R1	Yes
R2	Yes
R3	NA
ME1	NA
ME2	NA
CriV1	Yes
CriV2	Yes
CriV3	NA
ConV1	NA
ConV2	Yes
ConV3	Yes
ConV4	Yes
ConV5	Yes
Resp1	NA
Resp2	NA
Resp3	NA
Resp4	NA
Resp5	NA
Resp6	NA
Resp7	NA

For peer review only - http://bmjopen.bmj.com/site/about/guidelines.xhtml

Resp8	NA
Resp9	NA

For peer review only

For peer review only - http://bmjopen.bmj.com/site/about/guidelines.xhtml

COSMIN Reporting guideline for studies on measurement properties of patient reported outcome measures

Version August 2021

Joel J Gagnier[1-2]
Jianyu Lai[1]
Lidwine B. Mokkink[3]
Caroline B. Terwee[3]

1. Department of Orthopaedic Surgery, University of Michigan, Ann Arbor, MI, USA
2. Department of Epidemiology, School of Public Health, University of Michigan, Ann Arbor, MI, USA
3. Amsterdam UMC, Vrije Universiteit Amsterdam, Department of Epidemiology and Data Science, Amsterdam Public Health research institute, Amsterdam, Netherlands

Contact
Dr. Joel J. Gagnier, jgagnier@umich.edu
Website: www.cosmin.nl

For peer review only

Table of Content

List of abbreviations	2
Cite & Funding	3
Introduction	4
General reporting recommendations relevant for all studies on measurement properties	5
Specific reporting recommendations for studies on Content Validity	8
Specific reporting recommendations for studies on Structural Validity	8
Specific reporting recommendations for studies on Internal Consistency	8
Specific reporting recommendations for studies on Cross-cultural Validity\Measurement Invariance	9
Specific reporting recommendations for studies on Reliability	10
Specific reporting recommendations for studies on Measurement error	10
Specific reporting recommendations for studies on Criterion Validity	11
Specific reporting recommendations for studies on Hypotheses Testing for Construct Validity	11
Specific reporting recommendations for studies on Responsiveness	11
References	13

List of abbreviations

CTT:	classical test theory
IRT/Rasch:	Item Response Theory and Rasch analyses
NA:	not applicable
Original CC:	original COSMIN checklist [1]
PROM:	patient-reported outcome measure
RoB:	Risk of Bias; it refers to the COSMIN Risk of Bias checklist [2]

For peer review only - http://bmjopen.bmj.com/site/about/guidelines.xhtml

Cite:

Joel J Gagnier, Jianyu Lai, Lidwine B Mokkink, Caroline B Terwee. COSMIN reporting guideline for studies on measurement properties of patient-reported outcome measures. Qual Life Res. 2021 Aug; 30(8):2197-2218. doi: 10.1007/s11136-021-02822-4.

Funding:

This study was funded by a Methods Grant from the Patient-Centered Outcomes Research Institute.

For peer review only

For peer review only - http://bmjopen.bmj.com/site/about/guidelines.xhtml

Introduction

The COSMIN Reporting Guideline is recommended for reporting on studies that evaluate the measurement properties of existing patient-reported outcome measures (PROMs). Adequate reporting of scientific research will increase the applicability of and contributions to scientific knowledge[3]. Studies examining measurement properties are often missing key information that may allow a reader of these studies (e.g., clinician, scientist, funder) to determine what methods were used, what the results are and ultimately what the research means for the evidence of the quality of a particular PROM. This reporting guideline can improve and direct the reporting of studies investigating any measurement properties of PROMs. Improving the reporting of these studies increases their transparency and therefore makes obvious their risk of bias as well as their import to scientific knowledge. This allows accurate methodological assessment of these papers, reliable application of their findings (e.g., to clinical research) and also allows researchers to build on or improve future investigations in the area.

The COSMIN Reporting Guideline was developed as a detailed and specific reporting guideline, for all sections of a manuscript and for all measurement properties that can be investigated within studies exploring the measurement properties of PROMs. More information on this study can be found here[4].

The guideline is a set of 71 items for inclusion in a reporting guideline for studies on measurement properties of PROMs. It contains 35 common recommendations to be used for all studies on any of the measurement properties, and 36 specific recommendations divided into reporting of the specific measurement properties.
The common recommendations are divided into items for reporting the title (n = 3 items), abstract (n = 7), introduction (n = 6), methods (n = 8), results (n = 3), discussion (n = 6), conclusions (n = 1), and other information (n = 1); the specific recommendations are divided into reporting items concerning content validity (n = 7), structural validity (n = 2), internal consistency (n = 3), cross-cultural validity\ measurement invariance (n = 5), reliability (n = 3), measurement error (n = 2), criterion validity (n = 3), hypotheses testing for construct validity (n = 5), responsiveness (n = 6).

We hope all relevant international scientific groups adopt these recommendations and that relevant peer-reviewed journals endorse and enforce them as they have for other reporting recommendations (e.g., CON SORT).

For peer review only - http://bmjopen.bmj.com/site/about/guidelines.xhtml

General Reporting recommendations relevant for all studies on measurement properties		
**Item Number**	**Item Name**	**Item Description**
**Report section: Title**		
T1	Patient Reported Outcome Measure (PROM)	The name of the PROM instrument(s) (and version if relevant) being studied
T2	Measurement Property (MP)	What MPs are being studied or more generally, that MPs are being studied (if there are many properties being investigated, for example)
T3	Study sample	General description of relevant study sample characteristics (e.g., condition of interest, language) and also any intervention or exposure (e.g., treatments) if applicable.
**Report section: Abstract**		
A1	PROM	The name of the PROM instrument(s) (and version if relevant) being studied (i.e. the SF-36 or SF-12; language version) or if it concerns an item bank (e.g., PROMIS instruments). The type of instrument (e.g. a self reported questionnaire or interview).
A2	Measurement Property	What MPs are being studied or more generally, that MPs are being studied (if there are many properties being investigated, for example)
A3	Design	The type of study being used to test the properties (e.g., test-retest design, longitudinal study, cohort, cross sectional, case series, randomized etc.). Other details of the study design if relevant (intervention/exposure, description of comparison instruments, outcomes other than PROMs).
A4	Sample	Inclusion / exclusion criteria. General description of relevant study sample characteristics (e.g., condition of interest, geographic location, language, other relevant demographic and baseline characteristics)
A5	Methods	A brief description of the methods for investigating each MP including statistical analyses
A6	Results	The main results for all MPs investigated reporting statistics for each result with measures of precision where appropriate.
A7	Discussion/Conclusions	A brief description of the results in the context of existing evidence, main strengths and drawbacks and the need for future research on the PROM(s) investigated.
**Report section: Introduction**		
I1	Name and describe the PROM of interest	Specify the name, type, language, and version of the PROM being investigated and how it was developed. Describe the construct the PROM aims to measure and its subscales; describe the structure of the PROM (e.g., the number of factors, the number of items, scoring algorithm); describe relevant instructions (like time period), and number or type of response categories. State whether the PROM is based on a reflective or formative model. Note: This information may also appear in the methods section in greater detail.
I2	Target population	Describe the specific target population that the PROM was designed for. The authors need to provide the appropriate and necessary characteristics of this population.
I3	Citation for the original development of the PROM	The citation for the original development paper(s) should be provided and other highly relevant citations related to the quality of the specific PROM under investigation.
I4	State of Knowledge & Rationale	A description of the current scientific knowledge (what is known) regarding the MPs of? the PROM under investigation. The authors should provide a literature review or refer to a recent review of all existing evidence of the specific version (e.g., language, short

For peer review only - http://bmjopen.bmj.com/site/about/guidelines.xhtml

		form) of the PROM and explain why the new study is necessary and important. The rational for the current proposed study should be given.
I5	Definitions	Specialized terms should be defined or explained.
I6	Objectives and Hypotheses	State the specific objective(s) of the research and hypotheses related to the specific PROM under investigation.
**Report section: General Methods**		
GM1	Study Design	State the key elements of the study design
GM2	Participants	State how the participants were chosen; the inclusion and exclusion criteria. (e.g., if a PROM for a specific condition, then the eligibility and selection criteria should reflect this).
GM3	PROM administration	An explicit description of how and when the PROM(s) were administered (e.g., in what setting) including data collection devices/system used (e.g. paper based, electronic administration / ePRO) should be provided.
GM4	Data collection procedures	Provide information about other data collection, exposure methods (e.g., allocation to interventions) and time points / follow-up points.
GM5	Power/sample size calculation	Provide a power calculation for all MP analyses. Alternatively, if a rule of thumb is used, state it and the source/citation.
GM6	Statistical analyses	Statistical analyses and tests corresponding to all hypotheses or objectives for all MPs should be reported. Where appropriate, a cut-off for statistical significance should be reported (e.g., p-value less than 0.05). A description of all statistics to be used to estimate the magnitude and direction of effect should also be reported, together with measures of variability or precision. Report statistical package used.
GM7	Missing data	State approaches or plan for dealing with missing data.
GM8	Post hoc analysis	The report should specify analyses that used data after the data collection period concluded (i.e., if the analyses were post hoc; secondary data analyses) and describe the rationale for any post hoc analyses.
**Report section: General Results**		
GR1	Missing data	The amount and reasons for missing data should be explained for all analyses for all PROMs (or other outcome measurement instruments) and relevant groups.
GR2	Participant/patient Characteristics	The study patients' characteristics should be described, including baseline PROM scores.
GR3	Sample size	If one study contained analyses using different sample sizes, the authors should report the sample size for each analysis.
**Report section: Discussion**		
D1	MP evidence	Per measurement property the authors should compare the result to the criteria for good measurement properties (e.g., COSMIN criteria)[27], and determine if the specific MP is sufficient or not. Note: This information may also appear in the results section in greater detail in a table for example.
D2	Practical relevance	The authors need to discuss the practical relevance of the findings.
D3	Strengths and limitations	Strengths and limitations of the study should be discussed. For example, discuss if there were any significant potential biases in the study that could have impacted the results.
D4	Generalizability	Generalizability issues related to the PROM results should be discussed. For example, discuss if the results could be generalized to other populations given the sample studied.
D5	Instrument changes	Discuss the need for modifications to the existing PROM or new

For peer review only - http://bmjopen.bmj.com/site/about/guidelines.xhtml

		PROM development. If you conclude that one of the measurement properties is insufficient, you could suggest some modification, or if it is really poor, you could suggest stopping use of the PROM (in the specific population or in general).
D6	Future Research	Report specifically the type of research needed to answer new questions arising out of these findings for the particular MP and PROM investigated.
**Report section: Conclusions**		
C1	Conclusions	State the overall conclusions for each MP and of the use PROM investigated.
**Report section: Other information**		
O1	Conflict of Interest	State any relevant conflict of interest related to the PROM under investigation (e.g., an author being the PROM developer, funding body etc).

For peer review only

For peer review only - http://bmjopen.bmj.com/site/about/guidelines.xhtml

Specific Reporting recommendations for studies on Content Validity		
**Item Number**	**Item Name**	**Item Description**
CV1	Relevance	Report if and how patients and/or professionals were asked whether each item is relevant for their experience with the condition
CV2	Comprehensiveness	Report if and how patients and/or professionals were asked whether all key concepts are included
CV3	Comprehensibility	Report if and how the comprehensibility of the PROM instructions, items, response options, and recall period was assessed
CV4	Relevance results	Report if all items were considered relevant for the construct, population, and context of use of interest by patients and/or professionals
CV5	Response options and recall period	Report whether the response options and recall period were considered appropriate by patients and/or professionals
CV6	Comprehensiveness results	Report whether patients and/or professionals considered all key concepts to be included in the PROM
CV7	Comprehensibility results	Report whether patients understood the PROM instructions, items, and response options as intended

Specific Reporting recommendations for studies on Structural Validity		
**Item Number**	**Item Name**	**Item Description**
SV1	Factor Analyses: Classical Test Theory (CTT) PROMs	Report details of the methods and results for any exploratory or confirmatory factor analyses. State the rational for any explorative factor analyses (e.g., no clear a priori hypotheses). For CFA, describe and justify the factor structure of tested models. Methods and results for checking of the assumptions should be described, the method of estimation, goodness-of-fit statistics and cut-off points for good model fit, including factor loadings of best-fitting model.
SV2	Item Response Theory (IRT) analyses	Type of IRT/Rasch model should be reported. Also report the method of estimation, methods and results for checking of the assumptions (unidimensionality (see factor analysis), local dependency (e.g., residual correlations), monotonicity; (e.g. Mokken scaling), goodness-of-fit statistics, and cut-off points for goodness of item/model fit, and all item parameters.

Specific Reporting recommendations for studies on Internal Consistency		
**Item Number**	**Item Name**	**Item Description**
IC1	Unit of measurement	Report internal consistency methods and results for each unidimensional scale or subscale. Report all evidence or assumptions associated with unidimensionality.
IC2	Continuous scores	Report Cronbach's alpha or omega statistics. Report other statistics calculated for internal consistency of continuous scores.
IC3	Dichotomous scores	Report Cronbach's alpha or Kuder-Richardson coefficient. Report other statistics calculated for internal consistency of dichotomous scores.

For peer review only - http://bmjopen.bmj.com/site/about/guidelines.xhtml

Specific Reporting recommendations for studies on Cross-Cultural Validity\Measurement Invariance		
**Item Number**	**Item Name**	**Item Description**
CCV1	Comparator Group(s)	Report characteristics of (sub)groups being compared. Include sample sizes in each group.
CCV2	Factor Analyses: Classical Test Theory (CTT) PROMs	Report details of the methods and results for multiple-group confirmatory factor analyses, logistic regression analyses, or other analyses performed. Describe and justify the series of tested models, including constraints of factor loadings, intercepts and variances in CFA. Methods and results for checking of the assumptions should be described. criteria to define invariance. Describe the method of estimation, goodness-of-fit statistics and criteria used to flag items for measurement invariance.
CCV3	Item Response Theory (IRT) analyses	Type of IRT/Rasch model should be reported. Also report the methods and results for checking of the assumptions (unidimensionality (see factor analysis), local dependency (e.g., residual correlations), monotonicity; (e.g. Mokken scaling).. Describe statistical packages, method of estimation, criteria used to flag items for DIF, and methods and results of all model comparisons.

For peer review only - http://bmjopen.bmj.com/site/about/guidelines.xhtml

Specific Reporting recommendations for studies on Reliability		
**Item Number**	**Item Name**	**Item Description**
R1	PROM Administrations	Report the total number of measurements made and if the measurements were applied to the same samples using the same PROM. The process of administrating the measurements to the patients should be described, including who administered it (i.e., did the patient complete it or was there a proxy), when, how and any time intervals between administrations should be reported. This should include: time interval between repeated measurements (e.g., was the patient stable or not), the test type (e.g. a self-administered questionnaire, an interview-based PROM), the setting in which the instrument was administered (e.g., at the hospital, or at home), and the instructions given for completing it. If relevant, other instruments or measurements accompanying the repeated PROM measurement. Also, if relevant, the independence (whether the PROM was completed without knowledge of the previous scores) of the administrations.
R2	Statistical analyses	All statistical analyses and results specific to the reliability assessment(s) should be described and their use justified (e.g., the intraclass correlation coefficient (ICC) model or type of Kappa coefficient used). Also, describe the variance components, and the weighting scheme used for ordinal scores (e.g., linear or quadratic weights).
R3	Methods to improve reliability	Report any methods used to improve reliability such as restriction of the sample, training of researchers and standardization of methods, and averaging of repeated measurements.

Specific Reporting recommendations for studies on Measurement Error		
**Item Number**	**Item Name**	**Item Description**
ME1	PROM administrations	Report the total number of measurements made and if the measurements were applied to the same samples using the same PROM. The process of administrating the measurements to the patients should be described, including who administered it (i.e., did the patient complete it or was there a proxy), when, how and any time intervals between administrations should be reported. This should include: time interval between repeated measurements (e.g., was the patient stable or not), the test type (e.g. a self-administered questionnaire, an interview-based PROM), the setting in which the instrument was administered (e.g., at the hospital, or at home), and the instructions given for completing it. If relevant, other instruments or measurements accompanying the repeated PROM measurement. Also, if relevant, the independence (whether the PROM was completed without knowledge of the previous completion) of the administrations.
ME2	Statistical analyses	All statistical analyses and results specific to measurement error assessment(s) should be described and their use justified. Specifically, for continuous scores report the Standard Error of Measurement (SEM; Specify the exact model used to calculate the SEM (i.e., SEM consistency or SEM agreement)), Smallest Detectable Change (SDC; specify formula used, included the model of the SEM when based on the SEM) or Limits of Agreement (LoA). For dichotomous/nominal/ordinal scores report marginals (raw data) and the percentage specific (e.g. positive and negative) agreement.

For peer review only - http://bmjopen.bmj.com/site/about/guidelines.xhtml

Specific Reporting recommendations for studies on Criterion Validity		
**Item Number**	**Item Name**	**Item Description**
CriV1	Criterion	Report the details of the criterion used and why it was used. Justification of the gold standard must be reported. Also, describe (if applicable) how and why the criterion was dichotomized or classified. Also, how and when the criterion was administered (e.g., if independent from the PROM).
CriV2	Continuous scores	Report correlations (when criterion has continuous scores) or the area under the receiver operating characteristic (ROC) curve (when criterion is dichotomous).
CriV3	Categorical scores	Described how (and why) the PROM was dichotomized or made into multiple categories. Report sensitivity and specificity statistics.

Specific Reporting recommendations for studies on Hypotheses Testing for Construct Validity		
**Item Number**	**Item Name**	**Item Description**
ConV1	Comparator instrument(s)	The comparator instruments should be appropriately described in terms of the construct(s) they intend to measure. Report the measurement properties of the comparator instruments and related citations or data.
ConV2	Comparator Group(s)	Report characteristics of groups being compared. Include sample sizes in each group.
ConV3	Hypotheses	Report all hypotheses including the direction and magnitude of the expected correlations between the PROM of interest and another measurement instrument, or the direction and magnitude of differences in scores of the PROM between groups.
ConV4	Statistical analyses	Report all statistical methods and results used to test each hypothesis.
ConV5	Results	Report which specific results are in accordance with its hypothesis.

Specific Reporting recommendations for studies on Responsiveness		
**Item Number**	**Item Name**	**Item Description**
Resp1	Comparison Instrument(s)	The comparator instruments should be appropriately described in terms of the construct(s) they intend to measure. Report the measurement properties of the comparator instruments and related citations or data.
Resp2	Comparator Group(s)	Report characteristics of groups being compared. Include sample sizes in each group.
Resp3	Hypotheses	Report all hypotheses including the direction and magnitude of the expected correlations between changes in the PROM of interest and change in another measurement instrument, or the direction and magnitude of differences in change scores of the PROM between groups.
Resp4	Measurement procedures	Report if measurements were applied to the same sample using the same instruments. Describe the measurement procedures, including time intervals between different measurement instruments.
Resp5	Interim period	The interim period between time points should be described.
Resp6	Intervention/Exposure	Describe the intervention given or exposure in the interim period if relevant.
Resp7	Patients changed	Report the proportion of patients that improved or deteriorated (and the details of any anchor used) on the construct measured on all PROMs. Report any changes in

For peer review only

		scores of the PROM in the target population for the research application relative to the predefined hypotheses
Resp8	Statistical analyses	Report all statistical methods and results used to test each hypothesis.
Resp9	Results	Report which specific results are in accordance with its hypothesis.

For peer review only

References

1. Mokkink LB, Terwee CB, Patrick DL, et al. The COSMIN checklist for assessing the methodological quality of studies on measurement properties of health status measurement instruments: an international Delphi study. *Qual Life Res* 2010;19(4):539-49. doi: 10.1007/s11136-010-9606-8 [doi]
2. Mokkink LB, de Vet HCW, Prinsen CAC, et al. COSMIN Risk of Bias checklist for systematic reviews of Patient-Reported Outcome Measures. *Qual Life Res* 2018;27(5):1171-79. doi: 10.1007/s11136-017-1765-4
3. Ioannidis JP. How to make more published research true. *PLoS Med* 2014;11(10):e1001747. doi: 10.1371/journal.pmed.1001747 [published Online First: 2014/10/22]
4. Gagnier JJ, Lai J, Mokkink LB, et al. COSMIN reporting guideline for studies on measurement properties of patient-reported outcome measures. *Qual Life Res* 2021 doi: 10.1007/s11136-021-02822-4 [published Online First: 2021/04/06]

For peer review only
